# Minimized sample consumption for time-resolved serial crystallography applied to the redox cycle of human NQO1

**DOI:** 10.1038/s42004-026-01908-9

**Published:** 2026-01-29

**Authors:** Diandra Doppler, Alice Grieco, Domin Koh, Abhik Manna, Adil Ansari, Roberto Alvarez, Konstantinos Karpos, Hung Le, Mukul Sonker, Gihan K. Ketawala, Samira Mahmud, Isabel Quereda-Moraleda, Sayantee Sen, Angel L. Pey, Romain Letrun, Katerina Dörner, Jayanath C. P. Koliyadu, Raphael de Wijn, Johan Bielecki, Huijong Han, Chan Kim, Faisal H. M. Koua, Adam Round, Abhisakh Sarma, Tokushi Sato, Christina Schmidt, Mohammad Vakili, Dmitrii Zabelskii, Richard Bean, Adrian P. Mancuso, Joachim Schulz, Raimund Fromme, Milagros Medina, Thomas D. Grant, Petra Fromme, Richard A. Kirian, Sabine Botha, Jose Manuel Martin-Garcia, Alexandra Ros

**Affiliations:** 1 School of Molecular Sciences, Arizona State University, Tempe, AZ USA; 2 Center for Applied Structural Discovery, The Biodesign Institute, Arizona State University, Tempe, AZ USA; 3 Department of Crystallography and Structural Biology, Institute of Physical Chemistry Blas Cabrera, Spanish National Research Council (CSIC), Madrid, Spain; 4 Department of Physics, Arizona State University, Tempe, AZ USA; 5 Departamento de Química Física, Unidad de Excelencia en Química Aplicada a Biomedicina y Medioambiente e Instituto de Biotecnología, Universidad de Granada, Granada, Spain; 6 European XFEL GmbH, Schenefeld, Germany; 7 Center for Free-Electron Laser Science CFEL, Deutsches Elektronen-Synchrotron DESY, Hamburg, Germany; 8 Department of Chemistry and Physics, La Trobe Institute for Molecular Science, La Trobe University, Melbourne, VIC Australia; 9 Department of Biochemistry and Molecular and Cellular Biology, Faculty of Sciences and, Institute for Biocomputation and Physics of Complex Systems (BIFI), University of Zaragoza, Zaragoza, Spain; 10 Department of Structural Biology, Jacobs School of Medicine and Biomedical Sciences, SUNY University at Buffalo, Buffalo, NY USA; 11 Present Address: Diamond Light Source Ltd, Harwell Science and Innovation Campus, Didcot, UK

**Keywords:** X-ray crystallography, Techniques and instrumentation, Proteins

## Abstract

Sample consumption for serial femtosecond crystallography with X-ray free electron lasers remains a major limitation preventing broader use in macromolecular crystallography. This drawback is exacerbated in time-resolved (TR) experiments, where the amount of sample required per reaction time point is multiplied by the number of time points investigated. To reduce this limitation, we demonstrate a segmented droplet generation strategy coupled to a mix-and-inject approach for TR studies at the European XFEL. The injector produces synchronized droplet trains that enable stable and reproducible injection of protein crystal slurries at significantly reduced flow rates. Using the human flavoenzyme NAD(P)H:quinone oxidoreductase 1 (NQO1) as a test system, we collected diffraction data after mixing with NADH at 0.3 s and 1.2 s delays. The segmented injection approach achieved up to 97% reduction in sample consumption compared with continuous-flow injection while maintaining data quality suitable for TR crystallography. Reproducible electron density features consistent with low-occupancy NADH binding illustrate both the feasibility and the current limits of studying dynamic redox enzymes using this approach. This work establishes segmented droplet generation as a sample-efficient and XFEL-compatible method for future time-resolved serial crystallography experiments.

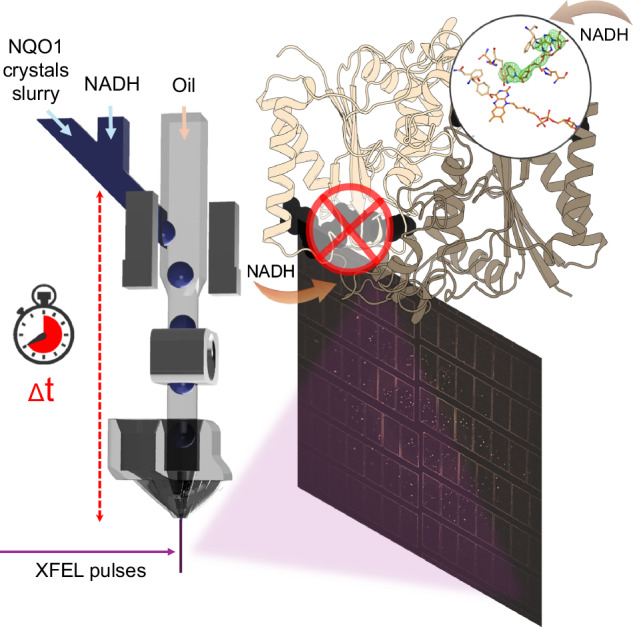

## Abstract (web summary)

Time-resolved serial femtosecond crystallography at X-ray free-electron lasers is a powerful approach for studying macromolecular dynamics, but its widespread use is limited by high sample consumption. Here, the authors introduce a segmented-droplet mix-and-inject strategy at the European XFEL that reduces sample consumption by up to 97% while preserving the data quality required for time-resolved structural studies of the enzyme NQO1.

## Introduction

Serial Crystallography both at X-ray free electron lasers (XFELs) and synchrotrons has developed into a robust tool for protein structural analysis^1–13^. It surpasses limitations in traditional goniometer-based crystallography approaches requiring large crystals, typically in the range of 50–200 μm or larger, where X-ray damage can be prohibitive and cryogenic approaches remain imperative^7,14^. Similarly, crystal defects become problematic in larger crystals required for traditional crystallography approaches, whereas in serial femtosecond crystallography (SFX) with XFELs, such effects remain suppressed as crystals in the order of 1–20 µm are typically employed^15,16^.

Furthermore, serial crystallography has allowed dynamic studies on proteins both for light-induced reactions as well as those induced through a substrate or binding partner^10^. To facilitate the latter approach termed mix-and-inject serial crystallography (MISC), the small crystals need to be mixed with a substrate or binding partner on time scales of milliseconds to initiate the reaction and then be probed by the X-ray beam at a certain time delay representing the time along the reaction coordinate^17^. Time-resolved (TR) experiments with MISC have been successfully used to study enzymatic reactions occurring on milliseconds-to-seconds timescales^18,19^. Some examples, among many, of this dynamic SFX technique include the reaction of the β-lactamase C from *Mycobacterium tuberculosis* with the antibiotic ceftriaxone^20,21^ and the inhibitor sulbactam^1^, the reaction of a riboswitch RNA with the substrate adenine^22^, the cytochrome C oxidase with O_2_^23^ and the isocyanide hydratase enzyme, which catalyzes the hydration of isocyanides^24^. Despite these advances, sample consumption remains a major limitation, particularly for multi–time point experiments, since each additional reation time point requires a new batch of sample, often consuming hundreds of milligrams of protein.

To this end, several approaches that allow the reduction of sample required for an SFX experiment have been described in the past years, which have been reviewed by Cheng et al.^25^. One of these approaches is the fixed-target approach in which a crystal slurry loaded onto a thin support to minimize background, is scanned in front of the X-ray beam^26,27^. Fixed-target devices have been successfully employed, and typically require only a few microliters ( < 1 mg of protein) of a crystal slurry to fill a chip from which a complete data set can be obtained. However, fixed-target devices may suffer from poor vacuum compatibility inducing dehydration, specifically when crystals are loaded between very thin support layers to reduce background or on non-enclosed devices to facilitate loading through wicking^25^. Additionally, fixed target devices are limited in the speed at which the chip can raster in front of the X-ray source, therefore, they are compatible with lower repetition rates. More importantly, since crystals are typically placed in an enclosed environment, fixed-target devices are impractical for TR-SFX experiments that require substrate mixing.

To sustain the advantages of established jet-based injection techniques, two main approaches that facilitate the reduction of sample consumption have been developed. The first approach are the viscous injectors, which allow the injection of crystal samples in a highly-viscous stream, extruding samples at flow rates ranging from just a few nL min^−1^ up to only a few µL min^−1^ ^27,28^. Originally developed for SFX experiments with membrane protein crystals grown in lipidic cubic phase, these injectors have also been successfully tested to inject soluble protein crystals with a variety of viscous media^14,29–33^ both at XFELs and synchrotron radiation sources. An advantage of the viscous jets is that they can be coupled with a laser source to perform TR experiments for light-sensitive proteins, however, they are also limited in the speed of extrusion and are therefore only compatible with lower repetition rates^34^. The second jet-based approach relates to the formation of droplets, ejecting them at a desired frequency to intersect with the X-ray beam. Free-standing droplets can be generated from nozzles with piezoelectric or acoustic actuation^35,36^. The droplets generated with these approaches are in the range of pL-nL, significantly reducing sample requirements. However, injection into a vacuum remains challenging, and clogging effects may hamper crystallography for samples injected in droplets.

Another special class of droplet generators divides sample-laden droplets within an immiscible oil stream. These techniques not only facilitate in situ droplet crystallization before X-ray diffraction but also enable crystal-containing droplets to mix with a substrate through droplet coalescence post-crystal formation, thereby enabling TR diffraction experiments^37^. Moreover, they are applicable for certain light-induced TR studies and experiments requiring crystal injection into a low background environment (vacuum). They are particularly useful when crystals cannot grow to the appropriate size and quality required in viscous injection media or when other experimental parameters hinder the use of fixed-target approaches. Additionally, these techniques are particularly suited for experiments where liquid injection with a gas dynamic virtual nozzle (GDVN) is required^27,28^. An intrinsic advantage of segmented flow droplet injection is that principles of continuous injection with a GDVN can directly be applied, such as established crystallization parameters (e.g., maintaining growth media and established crystal size), but also the characteristics of the created jet (such as jet velocity and jet thickness). Additionally, with small modifications, these devices can generate droplets that are compatible with both high and low-repetition rate XFELs. GDVN injection is a robust and continuously evolving technique, accounting for approximately 30% of the structures solved using SFX reported in the Protein Data Bank (PDB). This highlights its central role in advancing serial crystallography techniques and underscores the importance of further development to propel TR structural biology^12^.

SFX with the segmented droplet generation approach has indeed been demonstrated for several proteins so far, both at the Macromolecular Crystallography Instrument (MFX) at the Linac Coherent Light Source (LCLS) at SLAC National Accelerator Laboratory, and the European XFEL (EuXFEL), where sample savings between 60% and 75% have been demonstrated compared to continuous GDVN injection, respectively^2,38,39^. However, a TR crystallography experiment with segmented droplet injection has not been demonstrated to date. The capability to synchronize droplet generation with the 10 Hz pulse-train structure of the EuXFEL, noting its MHz intra-train pulse rate, offers the potential to reduce sample consumption by up to two orders of magnitude compared to continuous flow. Segmented droplet injection would thus be ideally suited for TR experiments based on the mix-and-inject principle at the EuXFEL^40,41^. Thus, it is our goal to establish a droplet injector with minimal sample consumption that would be available for routine user operation in TR-SFX experiments.

As a proof-of-concept, we used the human flavoenzyme NAD(P)H: quinone oxidoreductase 1 (NQO1) mixed with its coenzyme NADH to test the feasibility of this method. In a recent study, we have determined the first room-temperature structure of NQO1 in complex with NADH using serial synchrotron crystallography, where the structure was evaluated in combination with molecular dynamics simulations^42^. Although relevant information was discovered on NQO1 dynamics and mechanism, the solved structure was a static picture of the reaction with NADH. Thus, to fully unravel the NQO1 function, exploration beyond static structures is required. To this end, diffraction data were collected at the EuXFEL SPB/SFX instrument at two time points, 0.3 and 1.2 s after mixing with NADH, using a mix-and-inject segmented droplet injector. The previously established segmented droplet injection principle was extended to include a mixer element just upstream of the droplet generation region in one completely 3D-printed device. While only electron density consistent with partial NADH occupancy was observed, these experiments demonstrate that segmented droplet generation can be successfully applied to TR serial crystallography with up to 97% reduction in sample use compared to continuous injection. Our efforts are motivated by providing a sample conservation approach for SFX at the EuXFEL to take full advantage of the capabilities of this XFEL operating with MHz pulse trains repeating at 10 Hz frequency.

## Results and discussion

Previously, we successfully demonstrated the delivery of aqueous protein-crystal-containing droplets into the path of an XFEL both at the SLAC LCLS^2,38^ and the EuXFEL^39^. While synchronization was not optimized in both cases, a sample consumption reduction of up to 75% at the LCLS and up to 60% at the EuXFEL were obtained^2,38,39^. Here, we further improve droplet synchronization and apply the segmented droplet injection approach for TR-SFX at the EuXFEL. For this purpose, we combined the microfluidic droplet generator with an upstream mixer to facilitate TR crystallography following the mix-and-inject principle and applied it to study the reaction of NQO1 and its coenzyme NADH.

### Droplet generation with capillary coupled device at SPB/SFX

**Fig. 1:**
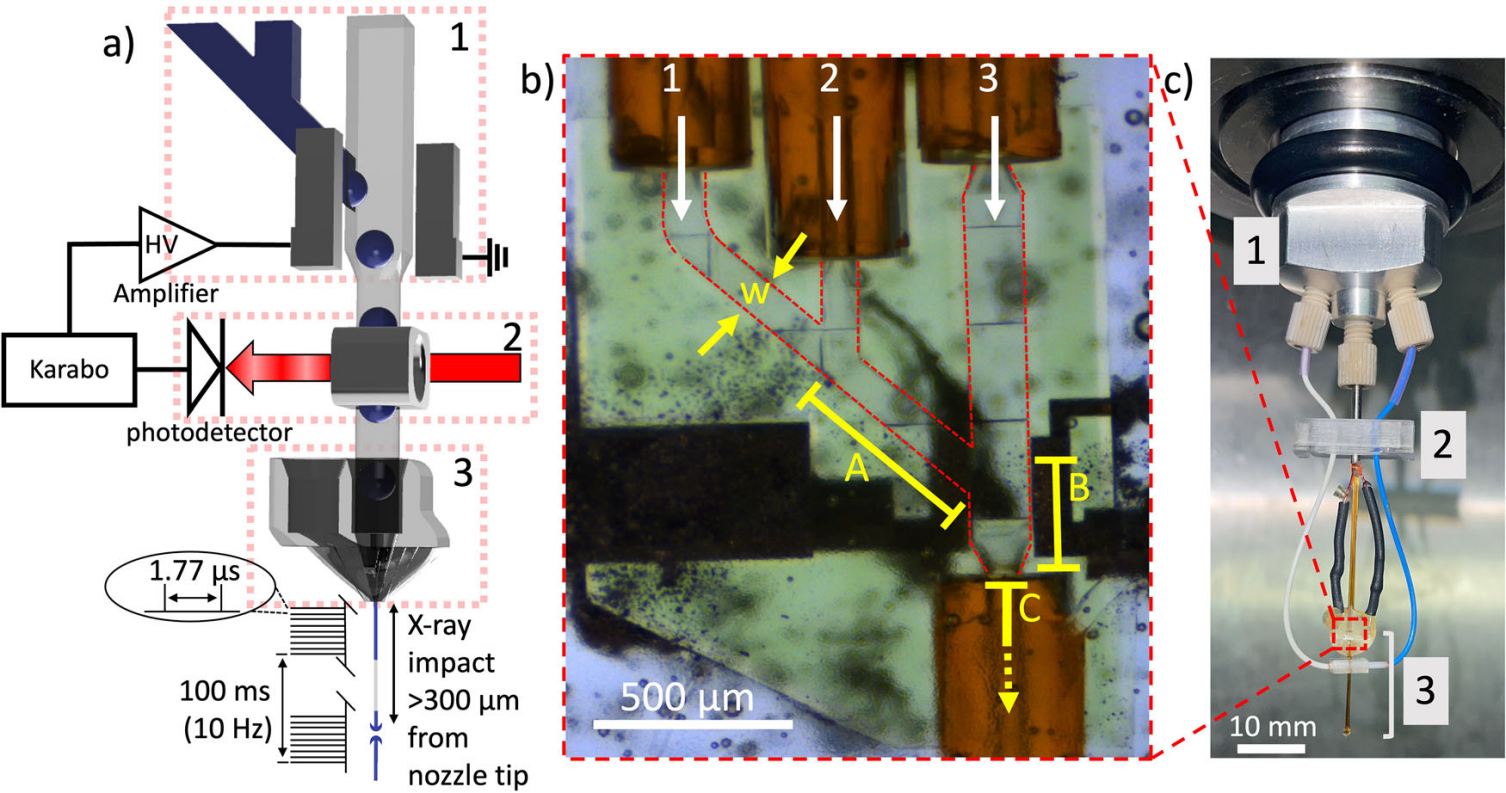
Droplet generation device and setup. **a** Schematic representation of the droplet generator setup with control hardware and X-ray interaction region in the SPB/SFX instrument at the EuXFEL highlighting the following components: (1) Y-Mixer droplet generator with integrated electrodes, (2) optical fiber droplet detector, and 3) 3D printed gas dynamic virtual nozzle. **b** Microscopy image of fully assembled DG250-Y-Mixer, illustrating the delivery capillaries of substrate (1), protein crystals (2), oil (3), and the exit point for mixed droplets (4). Critical dimensions are highlighted in yellow: w = 100 µm (width of the aqueous channel), A = 528 µm (length of channel section A within the mixer where substrate and crystal streams first mix), B = 285 µm (length of the channel section B where aqueous streams form a droplet segmented by the oil phase), and C = 15.4 mm (length of the remaining device, capillary, and region in the nozzle to the orifice of the GDVN in section C (not shown in full in the image)). **c** Injector mounted on the EuXFEL nozzle rod, featuring a custom adapter (1) and fiber bracket (2) securing the device and fibers (3) during insertion and experimentation.

A schematic representation of the droplet injector combined with the mixing unit is shown in Fig. 1a accompanied by the major components required for injection and synchronization as employed at the SPB/SFX instrument at the EuXFEL. Both the hardware and software elements allow the implementation of the feedback mechanism crucial for regulating droplet synchronization with X-ray pulses, a methodology previously employed at the LCLS^38^. In contrast to the 120 Hz XFEL repetition rate at LCLS, the EuXFEL operates on a distinctive pulse structure characterized by the delivery of a train of X-ray pulses at MHz repetition rate generated every 100 ms. Within the train, 202 X-ray pulses are separated by roughly 1.8 µs in the present experiments. Given this pulse structure, our approach diverges from introducing a droplet for each pulse; instead, we aim to introduce one droplet for every pulse train. This strategy ensures that the sample droplet spans the ~358 µs long train duration considering the apparent jet velocities and droplet volumes. Previously, characterization of the 3D-printed GDVNs with a 100 µm orifice demonstrated that jet velocities of ~25 m s^−1^ arise with the aqueous flow rates employed in this work (18–22 µL min^−1^) corresponding to gas mass flow rates of ~20 mg min^−1^ ^43,44^. Therefore, we calculated the distance traveled by a given sample volume in 358 µs to amount to 5 mm. A typical GDVN jet produced by a 3D printed nozzle is 3-10 µm in diameter^44–46^. Further assuming a rod-like droplet shape during jetting with a radius of 5 µm (the droplet is reduced in width from the 100 µm from the GDVN liquid orifice to ~5 µm in the jet), a minimum of 700 pL droplet volume will span the time the pulses are fired within a train. This principle is illustrated in Fig. 1a. As we further outline below, the droplet volumes created with the droplet generator span the entire pulse train.

To employ the droplet generators during the P3083 beam time at the SPB/SFX instrument in a mix-and-inject experiment, they were coupled to upstream microfluidic mixers to enable mixing of the NQO1 crystals with the substrate NADH (see Fig. 1a–b). The injector assembly features a Y-Mixer, bringing together the protein crystals and the substrate solution just before droplet generation. This configuration enables the execution of TR serial crystallography while also conserving the sample through droplet generation.

The reaction time points explored in these two experiments are primarily influenced by the flow rates used to propel the solutions into the XFEL path. To assess the reaction time point for a specific run, the average velocity within the channel was estimated from the volumetric flow rates employed during the experiment and the length and cross-section of the fluid channels. To obtain accurate estimates, three discrete sections (A-C) were defined, where changes in flow rate and channel cross-section may occur. These sections, as illustrated in Fig. 1b, encompass: *section A*, the length of the channel where the substrate initially encounters the protein crystal suspension with length A = 528 µm, *section B*, the length of the channel where the droplet is formed and accelerated by the oil phase with length B = 285 µm, and *section C*, the length of the capillary and nozzle before the droplet undergoes acceleration by the focusing gas within the GDVN with length C = 2 cm.

The first set of TR experiments was carried out with a mixer/droplet injector exhibiting a width, *w*, of 100 µm in *section A* and *w* of 150 µm in *section B*. This version was termed the DG250-Y-Mixer (Fig. 1b). While the droplet generator was designed to generate 10 Hz droplets, the operation of the DG250-Y-mixer at this frequency was not stable during the P3083 experiment in the SPB/SFX chamber. Nonetheless, droplet injection was continued at an average crystal flow rate (${Q}_{X}$) of 4.9 µL min^−1^, substrate flow rate (${Q}_{S}$) of 5.0 µL min^−1^, and oil flow rate (${Q}_{O}$) of 18.2 µL min^−1^ for about 40 min with the DG250-Y-Mixer. Without triggering but with droplet generation, these flow rates corresponded to a time point of about 0.3 s. A more detailed discussion quantitatively assessing the mixing times and probed reaction time point is presented below. The sample flow rate during droplet injection was about a factor of 6 lower than the total flow rate (${Q}_{T}$) employed. Compared to continuous sample injection with a GDVN at the same ${Q}_{T}$, approximately 83% less crystal sample was injected, resulting in the NQO1 structure at 0.3 s, which will be further discussed below.

### Droplet generator improvements

**Fig. 2:**
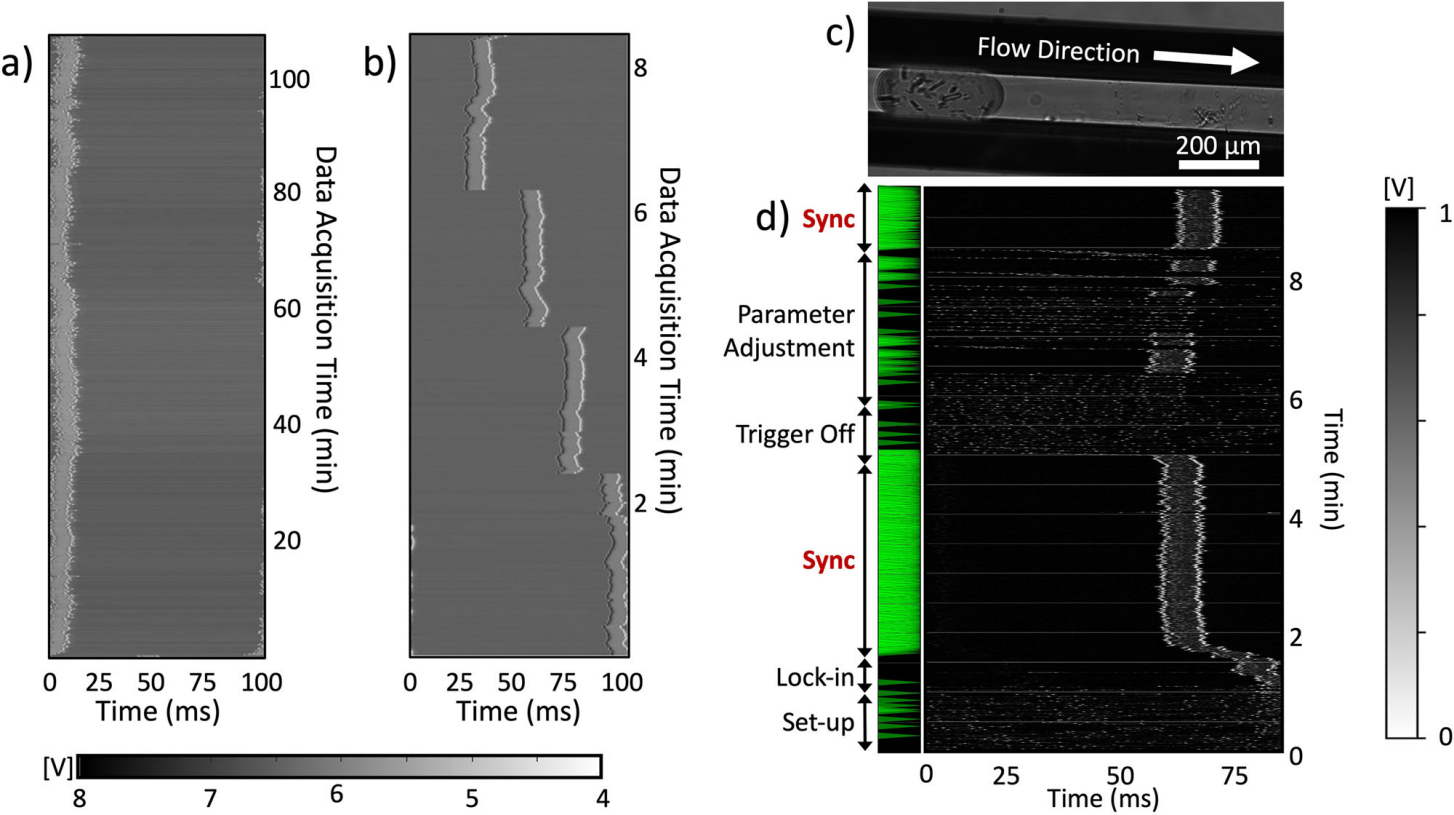
Triggered droplet generation and synchronization at 10 Hz. **a** Resulting waterfall plot in a droplet generation with the DG300-Y geometry and with ${Q}_{O}=18.2$ *µL min*^*-1*^, ${Q}_{B}=1.5$ µL min^−1^, trigger amplitude of 100 V and duration of 10 ms and **b** waterfall plot from a DG300-Y at ${Q}_{O}=18.5$ µL/min, ${Q}_{B}=1$ µL min^−1^, trigger amplitude of 200 V and duration of 10 ms demonstrating the shift of the droplet phase initiated by the triggering feedback system after the improvements in geometry and surface treatment. To establish the waterfall plot in (**a**) and (**b**), the droplet detector signal is divided into 100 ms sections according to the XFEL-reference at 10 Hz, and each trace is aligned in a vertical stack with the gray scale representing the amplitude of the signal. **c** Image of a droplet containing crystals originating from the DG-300-Y device recorded in the SPB/SFX instrument**. d** Waterfall plot of the start-up procedure conducted during P4502. The green triangles on the left indicate crystal hits. In all graphs, the time on the *x*-axis related to the delay time to the XFEL reference and the time on the y-axis to the data acquisition time.

To achieve 10 Hz droplet injection matching the EuXFEL X-ray pulse pattern, critical design optimizations were made to the droplet generator compared to our previous work at the LCLS^2,38^. Scanning Electron Microscopy (SEM) imaging identified a deformation in the wall separating the electrode and fluidic channels, as shown in Figure SI-1a–b. Therefore, the wall thickess was increased from 5 µm in previous realizations to the 10 µm in the devices employed here. Additionally, widening the aqueous and substrate channels to 150 µm improved droplet generation stability at 10 Hz compared to previous realizations of the droplet injector^2,38^. Surface treatment with NOVEC 1720 followed by thermal curing enhanced hydrophobicity and durability, critical for sustained operation. The improved DG300-Y-Mixer droplet generation devices were trigger-responsive, generated 10 Hz droplets with ~2.3 nL volume, and remained locked-in with respect to the phase of the XFEL reference for over 2 h as evidenced in the waterfall plot in Fig. 2a. This is important for long-term stability of the synchronization process during crystallography experiments. These tests were conducted using oil and NQO1 buffer in the EuXFEL SPB/SFX chamber. The good response to triggering was evidenced by the rapid change in droplet phase while maintaining lock-in along the waterfall plot as depicted in Fig. 2b. Further details on the design optimization are provided in the Supporting Information.

### TR-SFX with DG300-Y-mixers

DG300-Y-Mixers were subsequently employed in experiment P4502 at the EuXFEL to conduct TR crystallography on NQO1. The previously established feedback system was utilized to generate droplets at 10 Hz and to synchronize the droplets generated at that frequency with the EuXFEL pulse trains^38^. The droplet generation followed a similar start-up procedure, in which substrate, crystal sample, and oil flow rates were first increased beyond the target to initiate faster delivery of solutions. After a stabilization phase of approx. 10–15 min, the droplet generation frequency reached close to the target 10 Hz, and the electrical triggering system was initiated.

Figure 2d shows a representative waterfall plot for droplet injection at 10 Hz. In this representation, the droplet detector trace is divided into 100 ms sections according to the XFEL-reference at 10 Hz and each trace is aligned in a vertical stack with the gray scale representing the amplitude of the signal. As the flow rates stabilized at ${Q}_{X}=$1.00 µL min^−1^, ${Q}_{S}$ = 0.75 µL min^−1^, and ${Q}_{O}$ = 18.5 µL min^−1^, droplets were created near the 10 Hz target frequency. The external trigger was then activated with a duration of 4 ms, and an amplitude of 180 V. Droplets were stabilized just before 2 min maintaining stability for approx. 3 min without any system disturbance. Green triangles on the left, indicating crystal hits, confirm synchronization with the EuXFEL pulse structure for these 3 min, with 1631 diffraction patterns recorded at a hit-rate of 0.4%.

Following this stack of synchronized droplets, the trigger was turned off after 5 min, causing the droplets to immediately fluctuate in frequency and phase. A minute later, the trigger was re-activated with the same duration and delay, but with a reduced amplitude of 40 V. Although the droplet signal eventually locked-in about 30 s later, it did not remain stable, indicating that the reduced amplitude was not sufficient to stabilize the droplet-generation frequency lock-in. Similarly, at ~7.5 min, the amplitude was increased to 110 V, resulting in more frequent but still unstable lock-ins. Finally, at ~8.5 min, the amplitude was increased back to 180 V causing the droplets to immediately lock-in and synchronize as observed initially. This second instance of synchronization demonstrates the reproducibility of the synchronization process once droplets are generated at 10 Hz and the appropriate triggering conditions are applied. As illustrated in Figure SI-2, 10 Hz droplets could be generated through electrical triggering for durations of up to one hour as indicated through waterfall plots of 12 consecutive runs. These results validate the synchronization of the triggered droplet injection approach at 10 Hz at the SPB/SFX instrument. The long-term stability of the droplet synchronization was also explored.

### Time points for TR-SFX with Y-mixers

With the flow rate parameters described above to achieve synchronized droplet injection at 10 Hz, we will now discuss the time points probed in the reaction of NQO1 with NADH, as well as the mixing times achieved to initiate this reaction. The two DG-Y-Mixer devices can be broken down into three sections as previously outlined: *section A*, where the substrate and crystal stream first meet and mixing is initiated by diffusion in the convective flow, *section B*, where the droplet is formed— additional mixing may occur in the developing droplet from *section A* into *section B*, and *section C*, the path of the droplet through the remaining device prior to injection by the GDVN. The sum of time spent in each of the three sections based on the cross sectional area and the corresponding flow rates determines the average time point of the reaction probed, ${t}_{R}$, illustrated in Eq. 1:

1 $${t}_{R}={t}_{A}+{t}_{B}+{t}_{C}$$

Operation of the DG300-Y-Mixer resulted in residence time, ${t}_{R}$, of 1.2 s before irradiation with the XFEL pulses based on ${Q}_{X}$ = 0.5 µL min^−1^, ${Q}_{S}$ = 0.4 µL min^−1^, and ${Q}_{O}$ = 18.3 µL min^−1^ contributing to ${t}_{A}$, ${t}_{B}$ and ${t}_{C}$ as summarized in Table 1.

**Table 1:** Average residence times for solutions in Droplet generators

Droplet Generator	${t}_{A}$ (s)	${t}_{B}$(s)	${t}_{C}$ (s)	${t}_{R}$ (s)
DG300-Y-Mixer	0.792	0.020	0.378	1.2
DG250-Y-Mixer	0.032	0.014	0.259	0.3

We investigated the primary contribution to the spread of ${t}_{R}$ caused by diffusion and convective mixing in *section A* and *B*. Once incorporated in droplets, crystals are transported at the same velocity, thus the time variation in *section C* is negligible. To quantitatively determine the spread of ${t}_{R}$ due to the different mixing regimes, a numerical model and matching experiments with a fluorescent marker were employed. Since imaging droplet release during a serial crystallography experiment is not possible, we conducted mixing studies in the devices using fluorescence microscopy imaging under ambient laboratory conditions while mimicking fluid pumping and sample lines similar to conditions found at the SPB/SFX instrument.

**Fig. 3:**
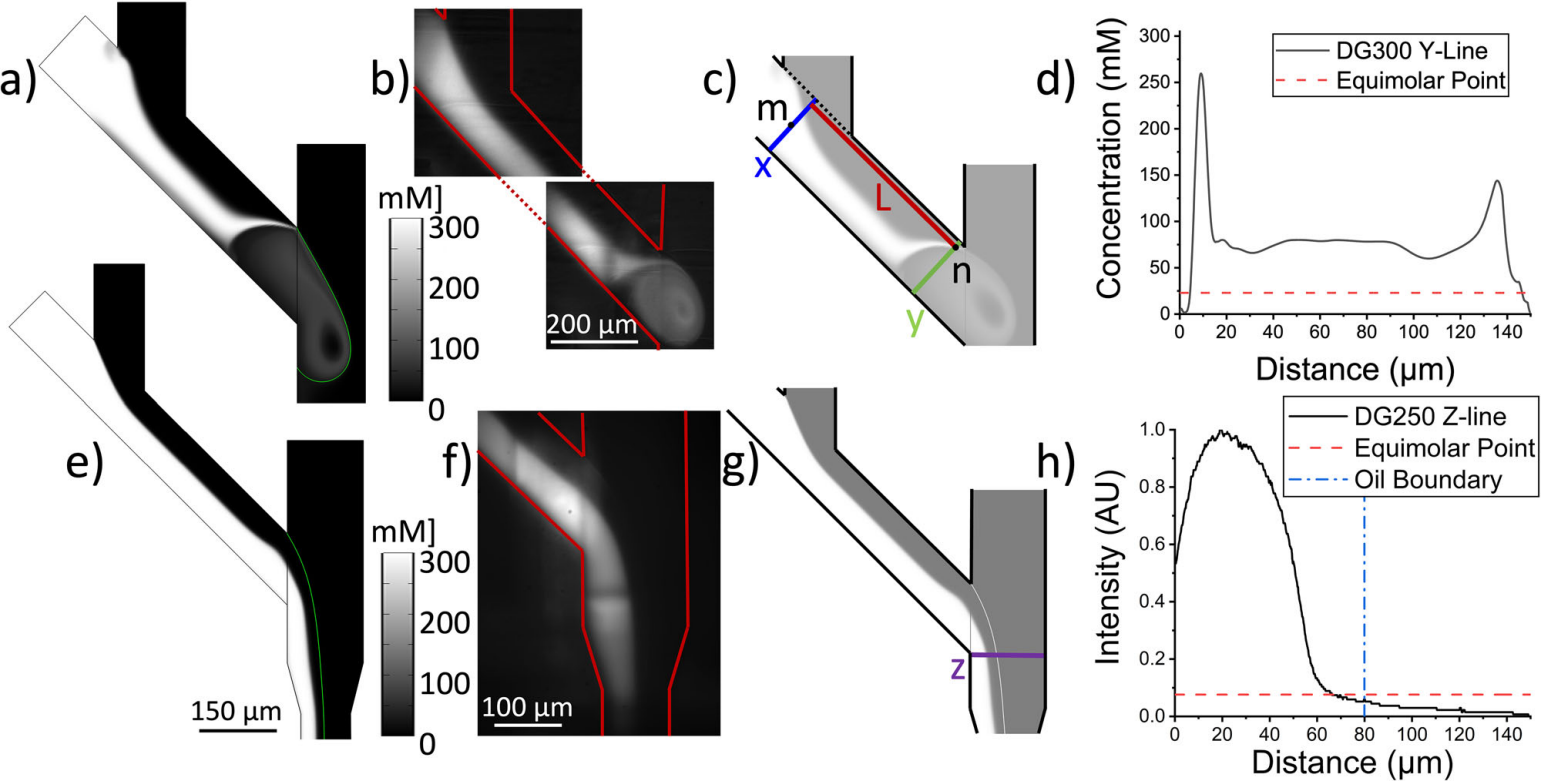
Mixing characteristics of protein crystals with substrate solution in the two droplet injector geometries. **a** The result of the 2D computational model for the substrate concentration within the DG300-Y-Mixer at a starting substrate concentration of 300 mM. **b** Fluorescence microscopy image of the diffusing species (fluorophore Alexa-Flour 532) within the DG300-Y-Mixer as observed under similar flow conditions as in (**a**). **c** Schematic of section A overlaid on the numerical concentration profile. Two lines are represented in color used to analyze the mixing behavior: blue line (partition x): defined as the line where substrate and protein streams first meet. red line (partition y): a boundary 20 µm from the channel edge defined as the nearest location proteins can flow based on their finate size (20 µm). Along partition x, point m at the center marks the earliest mixing point. Point n defines the point along partition y which is 20 µm from the channel wall. L marks the distance along the channel from partition x to y. Partition y marks the position in the channel where the substrate and crystals are mixed, defined as the point at which the substrate concentration equals the protein concentration within the crystals (23 mM in the case of NQO1). **d** concentration profile along partition y in the computational model where the equimolar point is indicated with a red dotted line. **e** The result of the 2D computational model for the substrate concentration within the DG250-Y-Mixer assuming a starting substrate concentration of 300 mM. **f** Fluorescence microscopy image of the diffusing species within the DG250-Y-Mixer carried out under similar flow conditions as (**e**) using the fluorophore Alexa-Flour 532. **g** Schematic of section A and B overlaid on the numerical concentration profile. Partition z marks the position in the channel analyzed for substrate mixing. **h** Normalzed concentration profile along partition z as obtained from experiment, where the equimolar point (where the substrate concentration surrounding the crystal and the protein concentration in the crystal are the same) and the oil boundary are indicated with a red and blue dotted line, respectively.

Figure 3a–b compare the numerical model with an image obtained during a mixing experiment at the same state of droplet formation. Qualitatively, we find an excellent agreement between model and experiment. Both model and experiment display a drift of the fluorescent solution (i.e., substrate) towards the corner of the Y-intersection with a vortex generated underneath from which the droplet is formed and eventually pinched off. The only major difference to the model observed in the fluorescence image (Fig. 3b) appears where darker streaks intersect the fluorophore brightness. Careful microscopy inspection of the devices revealed that these intensity differences result from stiching lines resultant from the 3D printing process where printing blocks merge.

We then investigated the quantitative differences between model and experimental mixing studies. First, we remark that the diffusion coefficient for NADH is reported by Siritanaratkul et al. with a value of $6.7* {10}^{-6}{{cm}}^{2}{s}^{-1}$ in an aqueous solution^47^. The NQO1 crystals (and substrate solution) are suspended within a mother liquor containing 18% PEG 3350, so we therefore corrected for the differences in visocity. According to the Einstein relation^48^ and the experimentally determined mother liquor viscosity of 5.39 m Pa s (unpublished data), we adjusted the NADH diffusion coefficient to $1.2* {10}^{-6}{{cm}}^{2}{s}^{-1}$ in the numerical model. We then compared the concentration profile obtained in the numerical model in *section A* at the same position in both numerical model and experimental mixing study. The position where the comparison was conducted is outlined in Fig. SI-4a. As evidenced by the comparison in Fig. SI-4b, the numerical modelling results are in very good agreement with the experimentally obtained concentration profile with the adjusted diffusion coefficient for NADH.

To estimate the mixing time spread, the concentration distribution arising from convection and diffusion at the experimentally employed flow rates was evaluated at two different positions within *section A*: *partition x* where the two aqueous streams meet (with *m* defined as the center position of this partition) and *partition y* selected as the position along the channel where equimolar concentrations of the substrate and protein (in the crystal) were reached at a distance of 20 µm from the channel wall—reflecting the finite crystal size of ~20 µm. *Point n* was chosen at this location representing the point at which complete mixing is assumed. The condition of equimolarity to determine mixing was previously investigated by Schmidt et al., and refers to the point where the substrate concentration surrounding the crystal and the protein concentration in the crystal are the same^49^. The protein concentration in NQO1 crystals was estimated to amount in 23 mM based on the NQO1 space group, thus equimolarity is defined where the substrate reaches the same value in the protein crystal. The two partitions where we performed the detailed substrate concentration analysis are represented in Fig. 3c.

The position, where the two solutions (crystal and substrate) first meet in the center of the channel in *section A* (along *partition x* at point *m* in Fig. 3c) signifies the earliest instance at which the reaction can initiate. Due to a parabolic flow profile, the mixing times of the substrate with crystals differed such that the crystals that are closer to the channel wall flowed at a lower velocity and, thus, mixed with the substrate later. Therefore, we determined both the slowest and fastest velocities of the aqueous flow and the resulting difference in residence time in *section A* contributing to the range in mixing times. Interestingly, due to the upward concentration drift close to the intersection where the droplet is pinched off, the equimolar concentration between substrate and protein concentration in the crystal is reached prior to the end of *section A*, at *partition y* at point n. This point was extracted from the model as the location where equimolarity was reached along that offset line, thereby determining the distance *L* over which the mixing time range was assessed. The concentration along *partition y* is plotted in Fig. 3d**(**Supplementary Data 1**)**, proving that the concentration of the substrate molecule was above the equimolar point throughout the width of the channel (at *partition y*). The residence times along *L* ranged from 0.161 s at the center line to 0.363 s near the wall, indicating that the reaction initiation was spread over ~200 ms in *section A*. We further conclude that mixing was accomplished prior to forming the droplet, as partition A is prior to the pinch of position of the droplet in *section B*.

In the DG250-Y-Mixer, the flow rates of ${Q}_{X}$ = 4.9 µL min^−1^ and ${Q}_{S}$ = 5.0 µL min^−1^ were used during the mixing experiment. Based on the device geometry and flow rates, the sample spends an average time ${t}_{A}$ = 32 ms in *section A*. Once in section B, with ${Q}_{O}$ of 18.2 µL min^−1^, the total flow rate ${Q}_{T}$ amounted to 28.1 µL/min leading to ${t}_{B}$ and ${t}_{C}$ of 0.014 s and 0.259 s respectively, and ${t}_{R}$ of 0.3 s (see also Table 1). Due to the different geometry and faster flow rates for the injection employed in the DG250-Y-Mixer, a different mixing behavior and droplet pinch-off resulted. The much faster aqueous flow rates did not lead to a droplet pinch off at the intersection, as demonstrated by fluorescence experiments and the numerical model, see Fig. 3e–f, but resulted in the two phases co-flowing^43^. The droplets were then pinched off about 2000 µm downstream in *section C*, as evidenced by bright-field microscopy imaging (see Fig. SI-5). The formation of droplets was confirmed during the TR-SFX experiment with the fiber optical droplet injector, however, as expected, synchronization was less pronounced.

Numerical modeling of the convection diffusion dynamics in the DG250-Y geometry (Fig. 3e) as well as experiments (Fig. 3f) with a dye matching diffusion properties of NADH also allowed us to estimate mixing times for the 300 ms time point. In the case of the DG250-Y geometry. We inspected the concentration distribution in *section B* at *line z*, as outlined in Fig. 3g, resultant from the experimental assessment of mixing through a fluorescent tracer similar to the DG300 case. The experimentally determined concentration distribution was assessed, as the co-flow condition in the model resulted in a smaller thickness of the aqueous stream where co-flow formed. As evidenced in Fig. 3h (Supplementary Data 1), the equimolarity condition was met at 68 µm and the oil/aqueous interphase occurred at 80 µm. This indicates, that the majority of NQO1 crystals (85%) had reached the equimolarity condition. The mixing time spread was assessed similar to the DG-300 geometry through the analysis of the corresponding fastest and slowest flow rates in section A revealing a time spread of 5.6 ms for the DG250-Y mixer.

In conclusion, for the 1.2 s time point in the DG300-Y-Mixer the total time spread, ${\Delta t}_{m}$, of ${t}_{R}$ resulted in ~200 ms and for the 0.3 s time point in the DG250-Y-Mixer ${\Delta t}_{m}$ resulted in ~6 ms. Lastly, we compare the variations in mixing times with the diffusion of the substrate into the protein crystals. For shoe-box shaped crystals with dimension of 10 × 20 × 30 µm^3^, diffusion times of ~15 ms were reported^49^. Taking this into account, we find that the diffusion into the crystals contributes margninally to ${\Delta t}_{m}$ in the DG-300-Y mixer case. In the case of the DG250-Y-mixer, the overall time spread is increased to ~21 ms, which corresponds to only 7% of the reaction time point (0.3 s), and which we expect to not influence the conclusions made on the structural analysis of the NQO1 reaction with NADH (as discussed below).

Based on the above presented analysis it is evident that the length of *section A* poses a limitation when attempting to investigate reactions at faster time points than reported here, specifically when operating the droplet generator at 10 Hz, where substrate and sample flow rates are small. Consequently, future efforts will involve the design and evaluation of mixing devices where the mixing occurs shortly before the droplet generation region. This approach aims to not only minimize overall reaction time but also restrict the time spread introduced by diffusive mixing of the samples. For example, in designs with channel dimensions similar to those for the DG300, but where the length of *section A* would be reduced from 528 µm to 100 µm, the time spread can be reduced to 46 ms.

### Sample consumption

For the 0.3 s and 1.2 s time points explored in our experiments, we further provide an assessment of sample consumption in the droplet injection mode, as shown in Table 2. While droplet generation was sustained throughout the experiment at the EuXFEL for several hours, the data presented in this table only includes runs where droplet generation was optimized for diffraction collection for the two specific time points targeted.

**Table 2:** Time points analyzed and sample consumption during P3083 and P4502

${t}_{R}$ (s)	Data collection time (min)	Indexed patterns	Patterns/µL	Protein concentration (mg mL^-1^)	Protein consumption (mg)	Resolution (Å)
0.3	38	18,794	101	18	3.2	2.7
1.2	85.5	10,992	257	25.5	1.7	2.5

For the 0.3 s time point, 3.2 mg of protein sample was consumed which is notably much less than that required from continuous injection with a GDVN, estimated to be 18.5 mg at the same total flow rate and time. This equates to nearly 6-fold sample savings despite the lack of droplet synchronization. At the ideal 10 Hz frequency with the DG300-Y-Mixer, droplet injection was recorded for about 1.5 h, generating nearly 11,000 patterns, resulting in 257 patterns per µL of sample injected. This was about 2.5 times more efficient than for the 0.3 s time point. Furthermore, for the 10 Hz scenario where the longer time point (1.2 s) in the reaction of NQO1 with NADH was probed, more than 60 mg of protein would have been consumed if a continuous GDVN were employed with a similar flow rate. Thus, droplet injection conserved 97% of the sample compared to a continuous injection demonstrating the advantage of using the droplet injector as a sample delivery device for mix-and-inject TR crystallography.

### Time-resolved structural insights into the binding of NADH to NQO1

Efficient reaction initiation in enzyme crystals requires fast incorporation of ligands to reach their active sites. In a prior *proof-of-principle* experiment, we observed that NQO1 microcrystals were suitable for TR experiments, as confirmed by binding of NADH to NQO1 after incubating crystal slurries with the coenzyme for 1 h before analysis^42^. Here, we used NQO1 as a test system to evaluate the performance of the segmented droplet injector for TR-SFX. Diffraction data were collected after mixing crystal slurries with NADH at time points of 0.3 s and 1.2 s, and compared to reference structures obtained from ligand-free NQO1 crystals. This experiment allowed us to assess both the mixing and injection performance of the new device and its ability to generate structural data with markedly reduced sample consumption.

NQO1 microcrystals belonged to the space group P2_1_2_1_2_1_ and showed two homodimers in the asymmetric unit (ASU) (Fig. SI-6) related by a non-crystallographic two-fold axis of symmetry^50^. All data collection, processing, and refinement parameters and statistics are given in Table SI-2. A high conformational heterogeneity of the residues at the two substrate binding sites, built at the interface of the two protomers of the NQO1 homodimer, were reported by our group in the free form of the protein^2,42^. To see if this is also observed in the free NQO1 structures reported here, we carried out a structural comparison of the structures (P3083 and P4502 experiments) with each other and with those previously determined. As illustrated in Fig. SI-7, and in good agreement with what we have previously reported^2,42^; the two active sites within each NQO1 homodimer displayed high conformational heterogeneity, particularly among residues forming the cofactor binding pocket (Asn64, Gln66, Tyr127, Arg201 and Phe233). The alternative conformations of Tyr127 and Tyr129, which gate the NADH pocket, again indicate substantial local flexibility even in the absence of ligand (Fig. SI-7). In fact, Tyr127 and Tyr129 are strictly conserved and are envisioned as key players in the function of NQO1^42,51,52^.

**Fig. 4:**
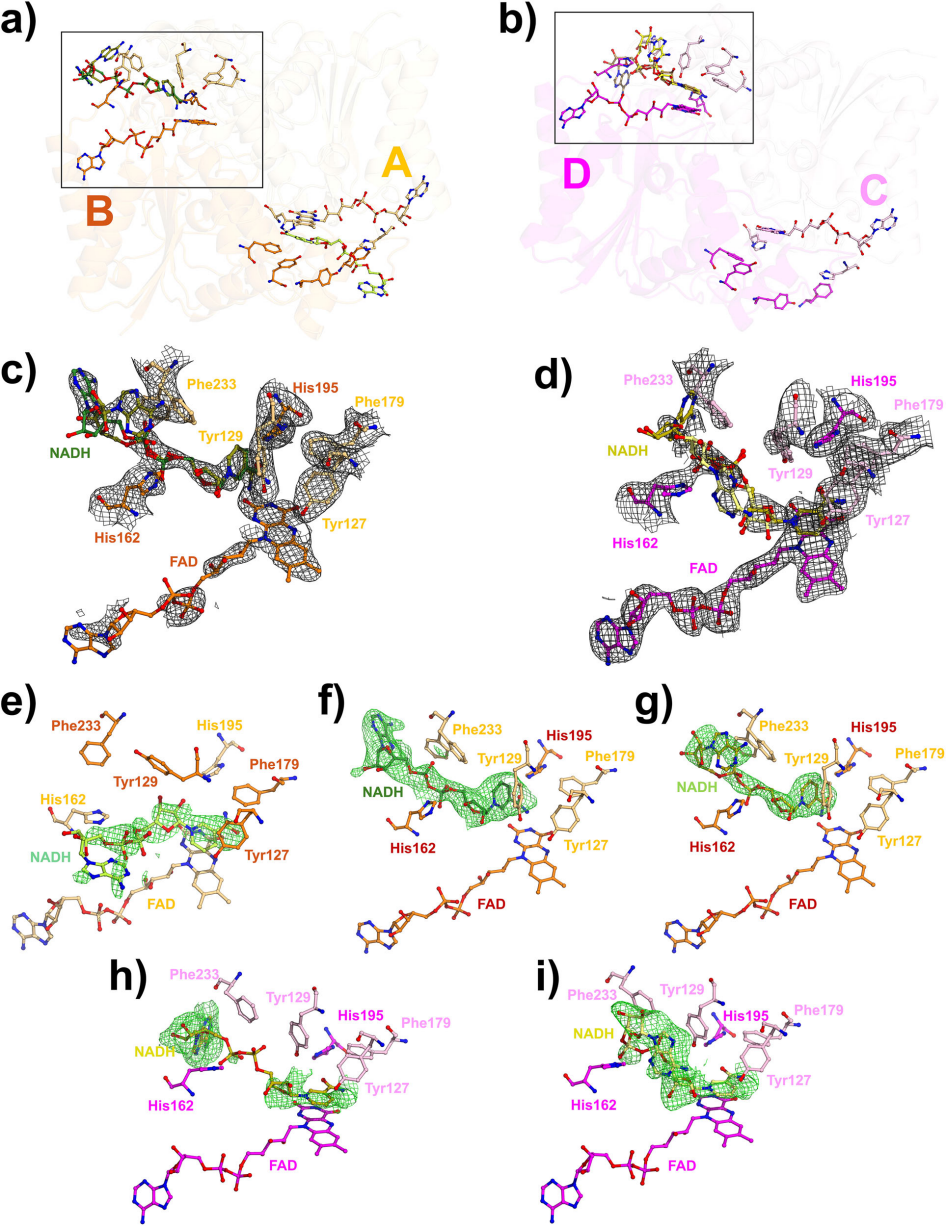
Time-resolved structure of NQO1 with NADH at 0.3 s. **a** Cartoon representation of homodimer 1, composed of chains A (light orange) and B (orange). **b** Cartoon representation of homodimer 2, composed of chains C (light pink) and D (magenta). In both homodimers, the NADH molecules bound to chains A, B, and D are shown as sticks and color-coded: yellow for chain A, dark green and olive for the two NADHs in chain B, and deep olive and pale yellow for the two NADHs in chain D. The FAD cofactor is also depicted as sticks. **c** Close-up view of the substrate binding site highlighted in panel (**a**) for chain B, showing the FAD, NADH, and substrate binding residues as sticks. The 2mFo-DFc electron density maps are contoured at 1 σ around the FAD and the substrate binding residues, and at 0.8 σ around the NADHs. **d** Close-up view of the substrate binding site highlighted in panel (b) for chain D, showing the FAD, NADH, and substrate binding residues as sticks. The 2mFo-DFc electron density maps are contoured at 1 σ around the FAD and the substrate binding residues, and at 0.8 σ around the NADHs. **e–g** Composite omit mFo-DFc electron density maps contoured at 3σ around the NADH molecules bound to the substrate binding site of chain A (**e**) and the two NADHs bound to chain B (**f** and **g**), which have been separated for clarity. **h–i** Composite omit mFo-DFc electron density maps contoured at 3σ around the two NADH molecules bound to the substrate binding site of chain D. The two NADH have been separated for clarity.

**Fig. 5:**
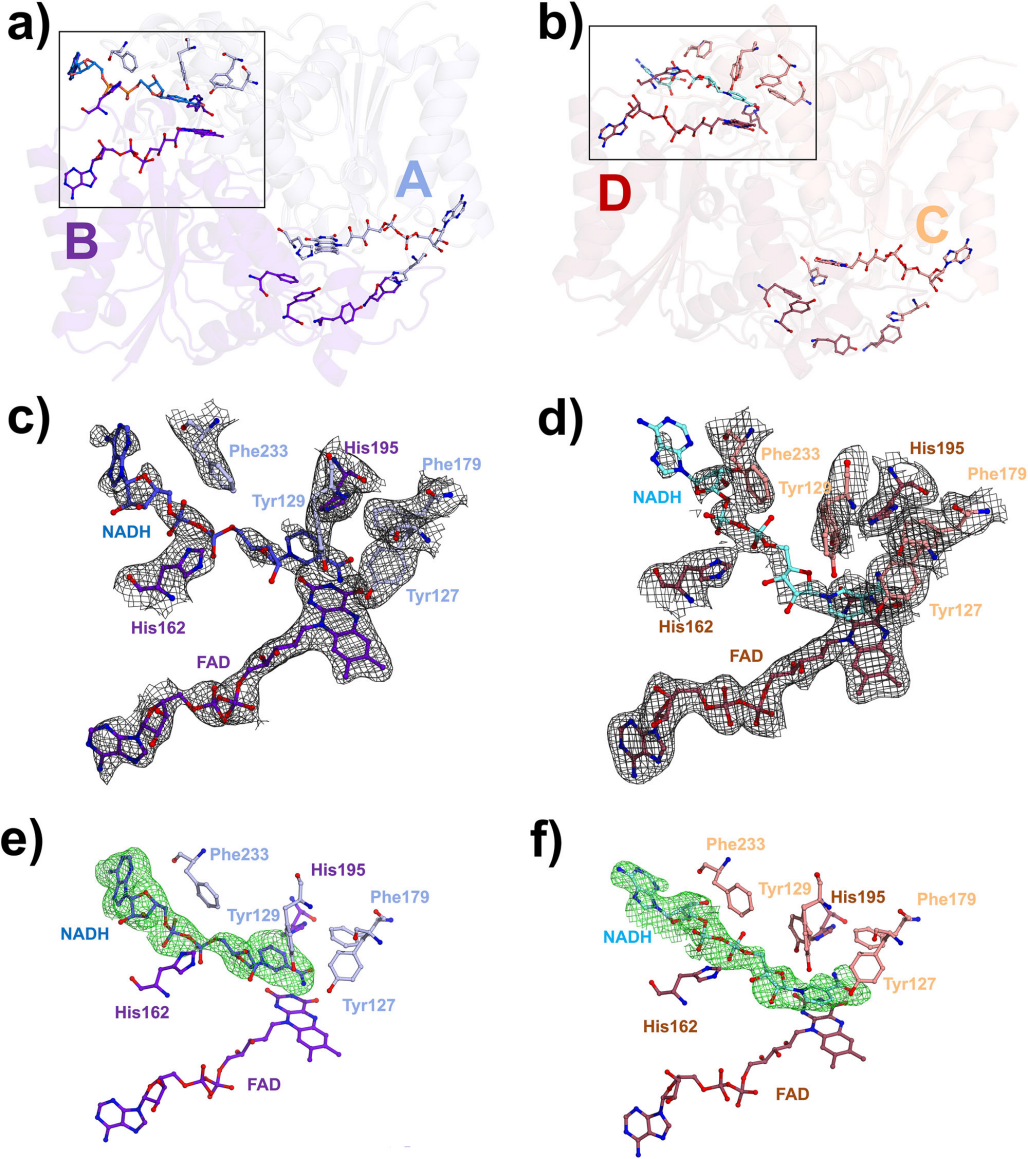
Time-resolved structure of NQO1 with NADH at 1.2 s. **a** Cartoon representation of homodimer 1, composed of chains A (light blue) and B (purple blue). **b** Cartoon representation of homodimer 2, composed of chains C (salmon) and D (raspberry). In both homodimers, the NADH molecules bound to chains B, and D are shown as sticks and color-coded in blue (chain B) and aquamarine (chain D). The FAD cofactor is also depicted as sticks. **c** Close-up view of the substrate binding site highlighted in panel (**a**), showing the FAD, NADH, and substrate binding residues as sticks. The 2mFo-DFc electron density maps are contoured at 1 σ around the FAD and the substrate binding residues, and at 0.8 σ around the NADH. **d** Close-up view of the substrate binding site highlighted in panel (**b**), showing the FAD, NADH, and substrate binding residues as sticks. The 2mFo-DFc electron density maps are contoured at 1 σ around the FAD and the substrate binding residues, and at 0.8 σ around the NADH. **e** Composite omit mFo-DFc electron density maps contoured at 3σ around the NADH molecule bound to the substrate binding site of chain B. **f** Composite omit mFo-DFc electron density maps contoured at 3σ around the NADH molecule bound to the substrate binding site of chain D.

In contrast to what has been reported by us for the structure of NQO1 with NADH at ${t}_{R}=$ 1 h, no significant difference in the unit cell dimensions between the free and NADH-mixed NQO1 samples was observed^42^. This could be due to the reduction in NQO1 plasticity observed upon NADH binding occuring over a longer timescale than the time points presented here. The resulting 2mFo-DFc experimental maps were of good quality, showing well-defined density for NADH binding site residues, the FAD cofactors, and solvent network (Figs. 4c-d; 5c–d; and Figs. SI-7, SI-8, and SI-9). Superposition of the free and NADH-bound NQO1 structures with earlier room-temperature structures (PDBs 8C9J and 8RFN for the free NQO1 and PDB 8RFM for the NQO1 in complex with NADH)^2,42^, yielded an average root-mean-square deviation (RMSD) of 0.294 Å for the Cα atoms, confirming that, overall, all NQO1 structures aligned very well with each other. When considering all protein atoms, the global RMSD increased to 0.590 Å, which suggests slightly higher structural differences when the whole protein molecule is considered, mainly due to mismatch from flexible loops as well as solvent-exposed regions. Composite OMIT mFo-DFc maps of the NADH molecules in the final model at 0.3 s and 1.2 s (Fig. 4e–i and Fig. 5e–f) suggest partial occupancy of NADH in certain sites, particularly within chains B and D, although the density is discontinuous and weaker than expected.

At 0.3 s time point, localized positive difference features were consistently observed near the coenzyme-binding pockets of chains A, B, and D, while no interpretable density was detected in chain C (Fig. 4), demonstrating rapid ligand diffusion and binding, despite the presence of a viscous mother liquor and the relatively large size of NADH. The pattern of these features appearing in three of the four active sites within the ASU suggests partial and heterogeneous engagement of NADH with NQO1. In chain A, contiguous though relatively weak density was observed along the adenosine and nicotinamide portions of the coenzyme, allowing a tentative placement of a single NADH molecule at 0.5 occupancy, contoured at 0.8 σ (Fig. SI-11). In chain B, the electron density was more complex, showing two overlapping conformations modeled at 0.5 occupancy each: one extended conformation with the adenosine moiety projecting toward the solvent channel, and another partially folded form in which the nicotinamide ring is oriented closer to the FAD isoalloxazine plane (Fig. 4c and SI-11). These alternative conformations likely represent distinct stages of coenzyme accommodation within the binding pocket, reflecting the dynamic balance between stacking interactions with Tyr127 and hydrophobic contacts with Phe106 and Phe233. Chain D displayed a similar heterogeneity, with two possible NADH conformations at roughly equal occupancy (Fig. 4d and SI-9). The electron density was continuous for parts of the adenine and ribose moieties but remained fragmented around the pyrophosphate linker, consistent with mobility of this region during early association. In contrast, the coenzyme binding site of chain C lacked discernible NADH density, even when maps were contoured at lower levels, implying that not all sites engage NADH simultaneously or with comparable occupancy. This observation reinforces the notion that NADH binding proceeds stochastically and that conformational sampling within each dimer can be asynchronous. The distinct electron-density patterns and conformations across chains A, B, and D thus highlight the intrinsic dynamics of NADH recognition by NQO1. Rather than a single, well-ordered binding event, the data suggest an ensemble of partially populated states consistent with transient and sequential engagement of the coenzyme. This interpretation is in line with previous biochemical and kinetic evidence showing that the two FAD cofactors within an NQO1 dimer are reduced at different rates, producing a functional asymmetry between protomers^53–56^. The observed site-to-site variation in NADH occupancy and orientation could therefore reflect differences in local microenvironments or subtle inter-subunit communication effects that modulate accessibility and binding affinity. Such a mechanism would enable the enzyme to regulate its redox cycle by alternating between active and “standby” protomers, thereby maintaining responsiveness to changing intracellular redox conditions.

At the 1.2 s time point, the electron-density landscape had evolved modestly, with ligand-like features remaining primarily in chains B and D (Fig. 5). Each of these sites contained a single NADH molecule modeled at partial occupancy (~ 0.5) and in a single predominant conformation. Although the overall density remained weak and discontinuous, it overlapped with the adenosine and nicotinamide positions identified at 0.3 s (Fig. 5 and SI-11), suggesting that a fraction of the coenzymes retained similar binding geometries. The resemblance between the maps at 0.3 s and 1.2 s, combined with the absence of clear new features, implies that NADH association stabilizes only slowly and with considerable conformational flexibility. This behavior is consistent with the high mobility of residues Tyr129 and Phe233, which flank the NADH pocket and can adopt alternative rotamers that either open or restrict access to the active site.

Notably, at both times after mixing, NADH-like features appeared preferentially in one active site per homodimer (chains B and D), consistent with the functional asymmetry of NQO1 reported in kinetic and spectroscopic studies, where the two FAD cofactors are reduced sequentially^53–56^. Slight shifts in residues lining the coenzyme pocket, including Tyr129, Phe233, and Arg201, accompany the appearance of these features, suggesting that subtle conformational adjustments may propagate across the dimer interface to regulate binding cooperativity. The persistence of weak, partially connected density at 1.2 s indicates that NADH remains dynamically associated rather than stably bound, reflecting a sampling of multiple low-occupancy states before achieving a catalytically competent geometry. This transient and asymmetric engagement is consistent with half-of-the-sites reactivity, allowing one protomer to remain poised for subsequent turnover while the other undergoes reduction. Importantly, we do not interpret the 0.3 s and 1.2 s structures as a sequential mechanistic series. Rather, they represent complementary structural snapshots of a highly dynamic and negatively cooperative enzyme, in which occupancy and NADH orientation vary intrinsically even under equilibrium conditions. Although these observations do not yet resolve distinct reaction intermediates, they provide early structural evidence that NQO1 leverages conformational flexibility and inter-subunit communication to coordinate redox activity, an insight that will guide future, higher-resolution time-resolved studies.

## Conclusion

In this study, we have demonstrated the successful application of segmented droplet generation as an effective method to facilitate mix-and-inject TR-SFX experiments at the SPB/SFX instrument at the EuXFEL. Unlike drop-on-demand methods, this approach uses a continuous stream of aqueous crystal-laden droplets separated by an oil phase, preserving the essential characteristics of liquid jet injection while ensuring compatibility with MHz repetition rates. Synchronization of droplets with the 10 Hz pulse trains of the EuXFEL was achieved for the 1.2 s reaction time point, enabling a time-resolved investigation of the NQO1 reaction with NADH. Structures of NQO1 with NADH bound were determined at a resolution of 2.5 Å, with sample consumption reduced by up to 97% compared to conventional continuous injection using GDVNs.

NQO1 structures obtained at the 0.3 s time point after mixing with NADH demonstrate the feasibility of using segmented droplet generation to probe early reaction events in enzyme crystals while dramatically reducing sample consumption by up to ~85%. Although droplet formation in this setup occurred at a 1.5 cm distance downstream from the y-intersection, the results validate this configuration as a practical route toward shorter, sub-100 millisecond reaction times, if this distance is minimized as in modular realizations of the droplet injector^2^. This modular and compact version^2^, where the distance from the droplet generation region to the nozzle orifice is in the order of 1 mm, will allow future experiments for studying reaction time points well below 100 ms. The upper limits in reaction time points may reach several seconds, only determined by the connecting capillary between droplet injector and 3D-printed nozzle, as outlined with the capillary coupled version in this manuscript. Future optimization efforts, such as relocating the convection-diffusion mixer closer to the droplet generation region, leveraging fast turbulent mixing within the droplets and fine tuning of droplet pinch off with adjustments in the oil phase viscosity, could further minimize reaction time spreads and eliminate the need for upstream mix-and-inject configurations. We also note that improvements of GDVN jet formation and stability in segmented flow applications is expected to increase hit rates under synchronized droplet injection.

Beyond the methodological advancements, this study paves the way to investigate how NQO1 interacts dynamically with NADH under near-physiological conditions. The present TR-SFX data reveal heterogeneous and partial coenzyme occupancy across the enzyme’s active sites, consistent with the inherent flexibility and functional asymmetry of NQO1 dimers. While a comprehensive mechanistic interpretation awaits further analyses, the results suggest that NADH engages the active sites through multiple transient conformations—an observation that resonates with the known NQO1 capacity for half-of-the-sites reactivity and adaptive redox behavior. Together, these findings establish segmented droplet injection as a viable and sample-efficient approach for studying enzyme catalysis in real time at XFELs, providing a methodological and conceptual framework for future investigations of a larger variety of enzymatic dynamics at XFELs with minimal material requirements ($\le \,$ mg per time point). Ongoing and complementary structural, spectroscopic, and computational analyses will extend this work toward a complete mechanistic understanding of the NQO1 redox cycle.

## Materials and methods

### Materials

Perfluorodecalin (PFD) and 1H,1H,2H,2H-perfluoro-1-octanol (perfluorooctanol, PFO) were purchased from Sigma-Aldrich (St. Louis, USA). SU-8 developer was obtained from Microchem (Round Rock, USA). The 3D printing resin, IP-S, was purchased from Nanoscribe GmbH (Eggenstein-Leopoldshafen, Germany). Deionized water (18 MΩ) was supplied from an LA755 Elga purification system (Elga Lab water, High Wycombe, USA), fluorphore ATTO 532 was purchased from ATTO-TEC (Siegen, Germany), and isopropyl alcohol (IPA) and ethanol were obtained from VWR International (Radnor, USA) or Decon Labs (King of Prussia, USA). Fused silica capillaries (360 µm outer diameter, 100 µm inner diameter) were purchased from Molex (Lisle, USA). Hardman extra-fast setting epoxy glue was purchased from All-Spec (Houston, USA).

*E. coli* BL21 (DE3) competent cells were purchased from Agilent technologies (USA). Yeast extract and tryptone were purchased from Condalab (Madrid, Spain). EDTA-free Protease Inhibitor Cocktail, Isopropyl β-D-1-thiogalactopyranoside (IPTG), ampicillin, sodium phosphate, sodium chloride, imidazole, flavin adenine dinucleotide (FAD), sodium acetate, K-HEPES, and Tris-HCl were purchased from Merck (Madrid, Spain). Polyethylene glycol (PEG) 3350 was purchased from Hampton Research (USA), and reduced nicotinamide adenine dinucleotide (NADH) was purchased from Merck (Madrid, Spain).

### Mixer device design and fabrication

Droplet generation devices that facilitate mixing with a substrate for TR studies were employed during the experiments. These devices were comprised of three components, as detailed in Fig. 1a. The first component integrated a droplet generator with a Y-shaped Mixer and also featured integrated electrodes as described previously^2,38^. The second component consisted of an optical fiber holder, and the third was a 3D printed GDVN. All components were connected with joining fused-silica capillaries and glued together using epoxy glue.

The components of the droplet generator and integrated mixers were designed and fabricated following established procedures^57^. Briefly, the devices were created using Fusion 360 (AutoDesk, San Francisco, USA) and subsequently 3D printed with a Photonic Professional GT 3D printer (Nanoscribe GmbH, Eggenstein-Leopoldshafen, Germany), employing IP-S photoresist. After printing, the devices underwent development in SU-8 developer followed by rinsing in IPA. Similar protocols were also employed for the GDVNs.

The droplet generator with an integrated mixer, named the DG-Y-Mixer, was comprised of three rectangular cross-section channels that converged in a series of Y-junctions, shown as a CAD rendering and a microscopy image, respectively, in Fig. 1a–b. The continuous channel delivered the oil phase that segmented the droplets, the center channel delivered the protein crystals suspended in their mother liquor, and the channel farthest from the oil channel delivered the substrate dissolved in the same mother liquor. The oil phase was a 10:1 v/v mixture of PFD to PFO.

Two different versions of the DG-Y-Mixer were used during the experiments. The DG250-Y-Mixer, depicted in Fig. 1a–c, contained aqueous channels that were 100 × 100 µm in cross-section that joined an oil channel with a cross-section of 150 × 150 µm. The second version, the DG300-Y-Mixer, depicted in Fig. SI-1c-d, contained channels of all the same cross-section of 150 × 150 µm.

### SEM imaging

SEM imaging was carried out at the Eyring Materials Center at Arizona State University, using the Zeiss Auriga FIB/SEM (Germany). Devices were printed with an open structure in the region of interest and then sputter-coated with gold. The SEM instrument was operated at 5.0 kV and the sample was tilted to inspect the wall between electrode and fluid channels.

### Fluidic operation and SPB/SFX configuration

Oil and crystal samples were driven from custom stainless-steel reservoirs^58^ by HPLC pumps (LC20AD, Shimadzu Co., Kyoto, Japan) or syringe pumps (neMESYS 1000, Cetoni, Korbussen, Germany). Bronkhorst liquid-flow sensors (mini CORI-FLOW™ ML120V00, Bronkhorst, Bethlehem, USA, and LIQUI- FLOW™ Mini, Bronkhorst, Bethlehem, USA) monitored flow rates in liquid lines before the reservoirs. PEEK tubing (Zeus, Orangeburg, USA, 250 μm ID, and 1/16-in OD), along with fittings and ferrules from IDEX Health & Science LLC (Oak Harbor, USA), connected the sensors, reservoirs, and tubing. Fused silica capillaries connected the reservoirs and the droplet generators.

For fluorescence experiments, DG-Y-Mixers were prepared on glass slides secured with epoxy where the observation region within the device remained unobstructed. Images and videos were acquired on an inverted microscope (IX71, Olympus, Tokyo, Japan) equipped with an Ocra-fusion BT digital camera (C15440, Hamamatsu Photonics, Shizuoka, Japan). The sample was excited using a 100 W mercury lamp (URFL-T, Olympus, Tokyo, Japan), 20× objective (LUCPlanFL N 20x, Olympus, Tokyo, Japan), and fluorescence filter sets for ATTO-532 (excitation filter FF02-510/20-25, dichroic FF562-Di02, and emission filter FF01-562/40-25, Semrock, Rochester, New York). ATTO 532 with a diffusion coefficient of $2.90\,{{{\rm{x}}}}{10}^{-6}{{{{\rm{cm}}}}}^{2}{{{{\rm{s}}}}}^{-1}$ was used for this study which is close to the one reported for NADH^59^. To generate droplets, oil and aqueous solutions containing 18% PEG with and without a fluorophore, matching the crystallography experiments, were driven from custom stainless-steel reservoirs by HPLC pumps (LC20AD, Shimadzu Co., Kyoto, Japan) or syringe pumps (neMESYS 1000, Cetoni, Korbussen, Germany) (Pump 11 Pico Plus Elite, Harvard Apparatus, Holliston, USA).

For beam times P4502 and P3083 at the EuXFEL SPB/SFX instrument, devices and fibers were mounted on a custom-made adapter at the end of a nozzle rod and lowered into the vacuum chamber through the instrument’s load-lock system^58^. Capillaries and insulated wires were fed through a steel tube affixed to the center of the adapter, while fibers were threaded through two additional side ports, as depicted in Fig. 1c. The stainless-steel reservoirs holding the protein crystal suspension were mounted on a rotating shaker device (PTR-360, Radnor, VWR). Helium pressure for the GDVN was regulated by a GP1 electronic pressure regulator (Equilibar, Fletcher, USA) and monitored by a Bronkhorst mass flow sensor (EL-FLOW Select F-111B, Bronkhorst, Bethlehem, USA). To display the long-term stability of droplet synchronization, the droplet detector signal in volts was represented in color in a line plot, and the lines stacked over time, resulting in a “waterfall” plot, where the time data collected corresponds to the time on the y-axis and the time from the start of a pulse train to the next (100 ms) is displayed on the x-axis. When locked-in, all droplets align at the same time with respect to the start of a pulse train. Waterfall plots are shown in Fig. 2 and Fig. SI-2.

### Feedback mechanism

During experiments conducted at the EuXFEL, the droplet monitoring and triggering feedback mechanism were fully integrated into Karabo, the in-house control system^60^. This triggering scheme resembles our previous approach with the Raspberry Pi^2,38^; however, with all controls integrated, the droplet trace, recorded by a SIS8300 digitizer (Struck Innovative Systeme GmbH, Hamburg, Germany), and additional diagnostic information were recorded and saved within the EuXFEL data stream for post-experimental processing^2,38^.

### Numerical modeling

Numerical modeling was used to assess the spread of the reaction time point with COMSOL Multiphysics Ver. 6.2 (COMSOL, Burlington MA, USA). A 2D model was employed to simulate the diffusion of the substrate molecule in the convective flow and forming droplet. The geometry of the DG250-Y-Mixer or DG300-Y-Mixer was created in AutoCAD (AutoDesk, San Francisco, CA, USA) at the center plane of each 3D device and imported into the corresponding COMSOL model.

The model consisted of three physics modules: the *Creeping Flow* module to establish the flow profile, the *Level Set* module for two-phase flow and droplet generation, and the *Transport of Diluted Species* module for modeling convection and diffusion. The geometry used for modeling droplet formation contained both *section A* and *section B*, with the dimensions outlined in Table SI-1. A physics-controlled mesh was used with the “extra fine” setting applied over the full length of the channel and the duration of the study was 1.75 s with 1 ms step size for the DG300-Y and 0.3 s with a 0.5 ms time step for the DG250-Y geometry. The model was built as previously reported^43^ but modified for the presented channel geometries and experimentally employed liquid phase parameters. The details of boundary conditions, relevant equations, and parameters are detailed in Table SI-1.

### Protein and crystal sample preparation

Protein expression and purification of human NQO1 were carried out as previously described^2^. For beam time P3083, microcrystals of the free NQO1 were obtained on-site in the XBI labs of the EuXFEL using the batch with agitation method as follows: in a 3 mL glass vial, 100 μL of the protein solution at 18 mg mL^-1^ were slowly added dropwise to 300 μL of the precipitant solution (0.1 M Tris pH 8.5, 0.2 M sodium acetate, 25% polyethylene glycol (PEG) 3350) while stirring at 200 rpm. Upon addition of the protein, the solution turned turbid immediately and needle-shaped crystals of an average length of 40 µm in their longest dimension grew at room temperature in about 1 h. Before loading the sample into the reservoir, the microcrystal samples were filtered through an inline filter (20 µm pore size) and then concentrated four times by centrifugation at 3000 rpm for 2 min to settle crystals. In the case of beam time P4502, a slightly different procedure was applied to produce the crystal sample. Smaller microcrystals of 10–20 µm in their longest dimension were grown on-site by adding a protein solution at a higher concentration (26.5 mg mL^−1^). Before loading the sample into the reservoir, the microcrystal samples were filtered through an in-line filter with a mesh cut-off size of 26 × 26 µm and further concentrated four times by letting them settle for about 4 h and subsequently removing the supernatant. The final crystal concentration for the experiments was 2 × 10^7^ crystals mL^−1^ in P3083 and 2 × 10^9^ crystals mL^−1^ in P4502. Absorbance measurements confirmed that all protein from the solution was incorporated into the crystals. For the mixing experiments, a highly concentrated solution of NADH at 300 mM was freshly prepared by weighing the coenzyme powder and mixing it with the corresponding crystallization buffer at 18% PEG 3350.

### Diffraction experiments

Experiments were conducted at the SPB/SFX instrument of the European XFEL during granted proposals P3083 and P4502^61^. During P3083, a pulse train containing 202 pulses spaced by 1.77 µs, was delivered at 10 Hz and 9.3 keV, with an ~3 µm spot size. The average pulse energy was 2.5 mJ with a duration of 40 fs separated by 1.77 µs, and diffraction data were collected on the AGIPD detector operating in fixed medium gain mode^62^. During experiment 4502, a pulse train containing 202 pulses spaced by 1.77 µs, was delivered at 10 Hz and 7 keV with a ~ 3.5 µm spot size. The average pulse energy was 1.8 mJ with a duration of 40 fs, and diffraction data were again collected on the Adaptive Gain Integrating Pixel Detector (AGIPD) detector operating in fixed medium gain mode^63^. During both experiments, live hit detection was monitored via OnDA^64^.

### X-ray diffraction data processing and structure determination

For structure determination, hits were initially identified by the Cheetah software after applying gain calibration^65^. A crystal hit was defined to satisfy the following criteria: Using Peakfinder 8, an ADC threshold of 150, a minimum SNR of 6, and a minimum of 1 pixel per peak to identify a minimum of 10 peaks in a resolution range of 0–500 (pixels) was applied. Additionally, to correlate hits with droplets for synchronization, a Python script was used to detect instances where the hits-per-train value, from Cheetah, was non-zero. This information was then overlaid with the droplet signal for each train, generating a waterfall plot. In this visualization, green triangles were used to indicate which droplets resulted in a Cheetah-identified hit. This procedure was applied in Fig. 2b.

Hits were subjected to indexing with CrystFEL v0.10.2. An additional round of peak finding with Peakfinder 8 using more robust parameters (ADC threshold of 250, a minimum SNR of 4, and a minimum of 2 pixels per peak needed to identify a minimum of 10 peaks in a resolution range of 10–800 pixels), and these peaks were subsequently supplied to indexing attempts using the algorithms Mosflm, XDS, DirAx and XGANDALF^66–71^. Intensities were integrated applying radii of 2, 4, and 6 and merged into pointgroup mmm using the CrystFEL program partialator, applying 1 iteration of the unity model.

A representative diffraction pattern for the complex NQO1-NADH is shown in Fig. SI-3. The final data collection and refinement statistics are listed in Table SI-2. During beam time P3083, two data sets, one for the free NQO1 and one for NQO1 mixed with NADH at a 0.3 s reaction time point were collected. For beam time P4502, two more data sets were collected, one for the free NQO1 and one for the NQO1 mixed with NADH at the 1.2 s time point. An overview of the data collected for both beam times presented in this manuscript is shown in Table SI-3.

All MTZ files for phasing and refinement were generated by the CTRUNCATE program from the CCP4 software package and a fraction of 5% reflections were included in the generated R_free_ set^72,73^. Initial phases of free NQO1 and NQO1 in complex with NADH were obtained by molecular replacement with MOLREP^74^. For the free NQO1 structures, we used our recently published SFX structure (PDB 8C9J) as the search model^2^. In the case of the dynamic structures, we used the free NQO1 structures from this study as the search model. The obtained models were refined using alternate cycles of automated refinement using non-crystallographic symmetry (NCS) with REFMAC5 and manual inspection was performed with COOT^75,76^. The final refined structures were validated using the Protein Data Bank (PDB) validation service prior to deposition. The atomic coordinates and structure factors have been deposited in the PDB with accession codes 9EZQ (Supplementary Data File 2) and 9EZS (Supplementary Data File 4) for the free NQO1 structures; 9EZR (Supplementary Data File 3) and 9ID0 (Supplementary Data File 6) for NQO1 in complex with NADH at 0.3 s, representing NADH in two distinct conformations; and 9EZT (Supplementary Data File 5) for NQO1 in complex with NADH at 1.2 s. It is important to note that, for all comparisons between the mixed datasets, the unmixed structure from experiment P3083 (PDB 9EZQ) was used as the sole reference state; the unmixed dataset from the second beamtime was not used for comparative analysis. Electron-density and composite OMIT mFo-DFc maps were calculated with the MAPS tool in the PHENIX software suite^77^. Structure figures presented in this manuscript were generated with PYMOL (version 2.4.0) (Schrödinger LLC).

### Reporting summary

Further information on research design is available in the Nature Portfolio Reporting Summary linked to this article.

## Supplementary information

Supplementary Material

Description of Additional Supplementary Files

supplementary data 1

supplementary data

supplementary data 3

supplementary data 4

supplementary data 5

supplementary data 6

Reporting Summary

## Data Availability

All data included in this manuscript or supplementary information will be available from the corresponding author upon reasonable request. The structure factors and the refined coordinates of the NQO1 structures in its free form and mixed with NADH have been deposited in the PDB under the accession codes 9EZQ, 9EZS, 9EZR, 9ID0 and 9EZT (see Supplementary Data Files 2-6).

## References

[1] Malla, T. N. et al. Heterogeneity in M. tuberculosis beta-lactamase inhibition by Sulbactam. *Nat. Commun.* **14**, 5507 (2023). 37679343 10.1038/s41467-023-41246-1 PMC10485065

[2] Doppler, D. et al. Modular droplet injector for sample conservation providing new structural insight for the conformational heterogeneity in the disease-associated NQO1 enzyme. *Lab Chip* **23**, 3016–3033 (2023). 37294576 10.1039/d3lc00176h PMC10503405

[3] Jernigan, R. J. et al. Room-temperature structural studies of SARS-CoV-2 protein NendoU with an X-ray free-electron laser. *Structure* **31**, 138–151 e135 (2023). 36630960 10.1016/j.str.2022.12.009 PMC9830665

[4] Wilamowski, M. et al. Time-resolved beta-lactam cleavage by L1 metallo-beta-lactamase. *Nat. Commun.* **13**, 7379 (2022). 36450742 10.1038/s41467-022-35029-3 PMC9712583

[5] Milano, S. K. et al. New insights into the molecular mechanisms of glutaminase C inhibitors in cancer cells using serial room temperature crystallography. *J. Biol. Chem.* **298**, 101535 (2022). 34954143 10.1016/j.jbc.2021.101535 PMC8784640

[6] Martin-Garcia, J. M. Protein dynamics and time resolved protein crystallography at synchrotron radiation sources: past, present and future. *Crystals* **11**10.3390/cryst11050521 (2021).

[7] Pearson, A. R. & Mehrabi, P. Serial synchrotron crystallography for time-resolved structural biology. *Curr. Opin. Struct. Biol.* **65**, 168–174 (2020). 32846363 10.1016/j.sbi.2020.06.019

[8] Rousseau, D. L., Ishigami, I. & Yeh, S. R. Structural and functional mechanisms of cytochrome c oxidase. *J. Inorg. Biochem* **262**, 112730 (2025). 39276716 10.1016/j.jinorgbio.2024.112730 PMC11896598

[9] Mous, S., Poitevin, F., Hunter, M. S., Asthagiri, D. N. & Beck, T. L. Structural biology in the age of X-ray free-electron lasers and exascale computing. *Curr. Opin. Struct. Biol.* **86**, 102808 (2024). 38547555 10.1016/j.sbi.2024.102808 PMC12854780

[10] Botha, S. & Fromme, P. Review of serial femtosecond crystallography including the COVID-19 pandemic impact and future outlook. *Structure* **31**, 1306–1319 (2023). 37898125 10.1016/j.str.2023.10.005 PMC10842180

[11] Schlichting, I. Serial femtosecond crystallography: the first five years. *IUCrJ* **2**, 246–255 (2015). 25866661 10.1107/S205225251402702X PMC4392417

[12] Barends, T. R. M., Stauch, B., Cherezov, V. & Schlichting, I. Serial femtosecond crystallography. *Nat. Rev. Methods Primers* **2**10.1038/s43586-022-00141-7 (2022). 10.1038/s43586-022-00141-7 PMC9833121 36643971

[13] Shi, Y. A glimpse of structural biology through X-ray crystallography. *Cell* **159**, 995–1014 (2014). 25416941 10.1016/j.cell.2014.10.051

[14] Martin-Garcia, J. M. et al. Serial millisecond crystallography of membrane and soluble protein microcrystals using synchrotron radiation. *IUCrJ* **4**, 439–454 (2017). 28875031 10.1107/S205225251700570X PMC5571807

[15] Weber, P. C. in *Methods in Enzymology* Vol. 276, 13–22 (Academic Press, 1997). 10.1016/S0076-6879(97)76048-8 27799088

[16] Drenth, J. & Haas, C. Protein crystals and their stability. *J. Cryst. Growth* **122**, 107–109 (1992).

[17] Kupitz, C. et al. Structural enzymology using X-ray free electron lasers. *Struct. Dyn.* **4**, 044003 (2017). 28083542 10.1063/1.4972069 PMC5178802

[18] Hejazian, M., Balaur, E. & Abbey, B. Recent advances and future perspectives on microfluidic mix-and-jet sample delivery devices. *Micromachines (Basel)* **12**10.3390/mi12050531 (2021). 10.3390/mi12050531 PMC8151207 34067131

[19] Park, J. & Nam, K. H. Recent chemical mixing devices for time-resolved serial femtosecond crystallography. *TrAC Trends Anal. Chem.* **172**10.1016/j.trac.2024.117554 (2024).

[20] Olmos, J. L. Jr. et al. Enzyme intermediates captured “on the fly” by mix-and-inject serial crystallography. *BMC Biol.* **16**, 59 (2018). 29848358 10.1186/s12915-018-0524-5 PMC5977757

[21] Pandey, S. et al. Observation of substrate diffusion and ligand binding in enzyme crystals using high-repetition-rate mix-and-inject serial crystallography. *IUCrJ* **8**, 878–895 (2021). 34804542 10.1107/S2052252521008125 PMC8562667

[22] Stagno, J. R. et al. Structures of riboswitch RNA reaction states by mix-and-inject XFEL serial crystallography. *Nature* **541**, 242–246 (2017). 27841871 10.1038/nature20599 PMC5502819

[23] Ishigami, I. et al. Snapshot of an oxygen intermediate in the catalytic reaction of cytochrome c oxidase. *Proc. Natl. Acad. Sci. USA* **116**, 3572–3577 (2019). 30808749 10.1073/pnas.1814526116 PMC6397517

[24] Smith, N. et al. Changes in an enzyme ensemble during catalysis observed by high resolution XFEL crystallography. *bioRxiv*10.1101/2023.08.15.553460 (2023). 10.1126/sciadv.adk7201 PMC10971408 38536910

[25] Cheng, R. Towards an optimal sample delivery method for serial crystallography at XFEL. *Crystals* **10**10.3390/cryst10030215 (2020).

[26] Cohen, A. E. et al. Goniometer-based femtosecond crystallography with X-ray free electron lasers. *Proc. Natl. Acad. Sci. USA* **111**, 17122–17127 (2014). 25362050 10.1073/pnas.1418733111 PMC4260607

[27] Martiel, I., Muller-Werkmeister, H. M. & Cohen, A. E. Strategies for sample delivery for femtosecond crystallography. *Acta Crystallogr. D. Struct. Biol.* **75**, 160–177 (2019). 30821705 10.1107/S2059798318017953 PMC6400256

[28] Echelmeier, A., Sonker, M. & Ros, A. Microfluidic sample delivery for serial crystallography using XFELs. *Anal. Bioanal. Chem.* **411**, 6535–6547 (2019). 31250066 10.1007/s00216-019-01977-x

[29] Conrad, C. E. et al. A novel inert crystal delivery medium for serial femtosecond crystallography. *IUCrJ* **2**, 421–430 (2015). 26177184 10.1107/S2052252515009811 PMC4491314

[30] Kovacsova, G. et al. Viscous hydrophilic injection matrices for serial crystallography. *IUCrJ* **4**, 400–410 (2017). 28875027 10.1107/S2052252517005140 PMC5571803

[31] Botha, S. et al. Room-temperature serial crystallography at synchrotron X-ray sources using slowly flowing free-standing high-viscosity microstreams. *Acta Crystallogr. D Biol. Crystallogr.* **71**, 387–397 (2015). 25664750 10.1107/S1399004714026327

[32] Martin-Garcia, J. M. et al. High-viscosity injector-based pink-beam serial crystallography of microcrystals at a synchrotron radiation source. *IUCrJ* **6**, 412–425 (2019). 31098022 10.1107/S205225251900263X PMC6503920

[33] Martin-Garcia, J. M. et al. Serial macromolecular crystallography at ALBA Synchrotron Light Source. Erratum. *J. Synchrotron. Radiat.* **29**, 1130 (2022). 35787581 10.1107/S1600577522005185 PMC9255580

[34] Weierstall, U. et al. Lipidic cubic phase injector facilitates membrane protein serial femtosecond crystallography. *Nat. Commun.* **5**, 3309 (2014). 24525480 10.1038/ncomms4309 PMC4061911

[35] Mafune, F. et al. Microcrystal delivery by pulsed liquid droplet for serial femtosecond crystallography. *Acta Crystallogr. D Struct. Biol.* **72**, 520–523 (2016). 27050131 10.1107/S2059798316001480

[36] Roessler, C. G. et al. Acoustic injectors for drop-on-demand serial femtosecond crystallography. *Structure* **24**, 631–640 (2016). 26996959 10.1016/j.str.2016.02.007 PMC4920001

[37] Stubbs, J. et al. Droplet microfluidics for time-resolved serial crystallography. *IUCrJ* **11**, 237–248 (2024). 38446456 10.1107/S2052252524001799 PMC10916287

[38] Sonker, M. et al. Electrically stimulated droplet injector for reduced sample consumption in serial crystallography. *Biophys. Rep.* **2**, 100081 (2022). 10.1016/j.bpr.2022.100081 PMC9680787 36425668

[39] Echelmeier, A. et al. Segmented flow generator for serial crystallography at the European X-ray free electron laser. *Nat. Commun.* **11**, 4511 (2020). 32908128 10.1038/s41467-020-18156-7 PMC7481229

[40] Mills, G., Bean, R. & Mancuso, A. P. First experiments in structural biology at the European X-ray free-electron laser. *Appl. Sci.* **10**10.3390/app10103642 (2020).

[41] DESY Dt. Elektr.-Synchr., H. *XFEL: The European X-Ray Free-electron Laser. Technical Design Report* (2006).

[42] Grieco, A. et al. Structural dynamics and functional cooperativity of human NQO1 by ambient temperature serial crystallography and simulations. *Protein Sci.* **33**, e4957 (2024). 38501509 10.1002/pro.4957 PMC10949395

[43] Doppler, D. et al. Co-flow injection for serial crystallography at X-ray free-electron lasers. *J. Appl. Crystallogr.* **55**, 1–13 (2022). 35153640 10.1107/S1600576721011079 PMC8805165

[44] Vakili, M. et al. 3D printed devices and infrastructure for liquid sample delivery at the European XFEL. *J. Synchrotron. Radiat.* **29**, 331–346 (2022). 35254295 10.1107/S1600577521013370 PMC8900844

[45] Knoska, J. et al. Ultracompact 3D microfluidics for time-resolved structural biology. *Nat. Commun.* **11**, 657 (2020). 32005876 10.1038/s41467-020-14434-6 PMC6994545

[46] Nazari, R. et al. 3D printing of gas-dynamic virtual nozzles and optical characterization of high-speed microjets. *Opt. Express* **28**, 21749–21765 (2020). 32752448 10.1364/OE.390131 PMC7470680

[47] Siritanaratkul, B. et al. Transfer of photosynthetic NADP(+)/NADPH recycling activity to a porous metal oxide for highly specific, electrochemically-driven organic synthesis. *Chem. Sci.* **8**, 4579–4586 (2017). 30155220 10.1039/c7sc00850c PMC6100256

[48] Atkins, P. W. *Physical Chemistry* 5th edn (Oxford University Press, 1994).

[49] Schmidt, M. Mix and inject: reaction initiation by diffusion for time-resolved macromolecular crystallography. *Adv. Condens. Matter Phys.* **2013**, 1–10 (2013).

[50] Bianchet, M., Faig, M. & Amzel, L. M. Structure and mechanism of NAD[P]H:Quinone Acceptor Oxidoreductases (NQO). *Methods Enzymol.* **382**, 144–174 (2004). 10.1016/S0076-6879(04)82009-3 15047101

[51] Pandey, P. et al. Potential modulation of human NAD[P]H-Quinone Oxidoreductase 1 (NQO1) by EGCG and its metabolites-A systematic computational study. *Chem. Res. Toxicol.* **33**, 2749–2764 (2020). 32975120 10.1021/acs.chemrestox.9b00450

[52] Asher, G., Tsvetkov, P., Kahana, C. & Shaul, Y. A mechanism of ubiquitin-independent proteasomal degradation of the tumor suppressors p53 and p73. *Genes Dev.* **19**, 316–321 (2005). 15687255 10.1101/gad.319905 PMC546509

[53] Pey, A. L., Megarity, C. F. & Timson, D. J. FAD binding overcomes defects in activity and stability displayed by cancer-associated variants of human NQO1. *Biochim Biophys. Acta* **1842**, 2163–2173 (2014). 25179580 10.1016/j.bbadis.2014.08.011

[54] Claveria-Gimeno, R., Velazquez-Campoy, A. & Pey, A. L. Thermodynamics of cooperative binding of FAD to human NQO1: Implications to understanding cofactor-dependent function and stability of the flavoproteome. *Arch. Biochem. Biophys.* **636**, 17–27 (2017). 29100982 10.1016/j.abb.2017.10.020

[55] Pacheco-Garcia, J. L. et al. Counterintuitive structural and functional effects due to naturally occurring mutations targeting the active site of the disease-associated NQO1 enzyme. *FEBS J.* **290**, 1855–1873 (2023). 36378023 10.1111/febs.16677

[56] Anoz-Carbonell, E., Timson, D. J., Pey, A. L. & Medina, M. The catalytic cycle of the antioxidant and cancer-associated human NQO1 enzyme: hydride transfer, conformational dynamics and functional cooperativity. *Antioxidants (Basel)* **9**10.3390/antiox9090772 (2020). 10.3390/antiox9090772 PMC7554937 32825392

[57] Echelmeier, A. et al. 3D printed droplet generation devices for serial femtosecond crystallography enabled by surface coating. *J. Appl. Crystallogr.* **52**, 997–1008 (2019). 31636518 10.1107/S1600576719010343 PMC6782075

[58] Schulz, J. et al. A versatile liquid-jet setup for the European XFEL. *J. Synchrotron Radiat.* **26**, 339–345 (2019). 30855241 10.1107/S1600577519000894 PMC6412181

[59] Maddala, B. G. et al. Evidence for nanostructures of at least 20 nm in a phosphonium ionic liquid at room temperature using fluorescence correlation spectroscopy. *J. Phys. Chem. B* **128**, 11197–11207 (2024). 39495867 10.1021/acs.jpcb.4c04950

[60] Hauf, S. et al. The Karabo distributed control system. *J. Synchrotron Radiat.* **26**, 1448–1461 (2019). 31490132 10.1107/S1600577519006696

[61] Mancuso, A. P. et al. The single particles, clusters and biomolecules and serial femtosecond crystallography instrument of the European XFEL: initial installation. *J. Synchrotron. Radiat.* **26**, 660–676 (2019). 31074429 10.1107/S1600577519003308 PMC6510195

[62] Allahgholi, A. et al. The adaptive gain integrating pixel detector at the European XFEL. *J. Synchrotron Radiat.* **26**, 74–82 (2019). 30655470 10.1107/S1600577518016077 PMC6337892

[63] Schwandt, J., Fretwurst, E., Klanner, R. & Zhang, J. Design of the AGIPD sensor for the European XFEL. *J. Instrum.* **8**, C01015–C01015 (2013).

[64] Mariani, V. et al. OnDA: online data analysis and feedback for serial X-ray imaging. *J. Appl. Crystallogr.* **49**, 1073–1080 (2016). 27275150 10.1107/S1600576716007469 PMC4886993

[65] Barty, A. et al. Cheetah: software for high-throughput reduction and analysis of serial femtosecond X-ray diffraction data. *J. Appl. Crystallogr.* **47**, 1118–1131 (2014). 24904246 10.1107/S1600576714007626 PMC4038800

[66] White, T. A. Processing serial crystallography data with CrystFEL: a step-by-step guide. *Acta Crystallogr. D Struct. Biol.* **75**, 219–233 (2019). 30821710 10.1107/S205979831801238X PMC6400257

[67] White, T. A. et al. CrystFEL: a software suite for snapshot serial crystallography. *J. Appl. Crystallogr.* **45**, 335–341 (2012).

[68] Powell, H. R., Johnson, O. & Leslie, A. G. Autoindexing diffraction images with iMosflm. *Acta Crystallogr. D Biol. Crystallogr.* **69**, 1195–1203 (2013). 23793145 10.1107/S0907444912048524 PMC3689522

[69] Kabsch, W. Xds. *Acta Crystallogr. D Biol. Crystallogr.* **66**, 125–132 (2010). 20124692 10.1107/S0907444909047337 PMC2815665

[70] Duisenberg, A. J. M. Indexing in single-crystal diffractometry with an obstinate list of reflections. *J. Appl. Crystallogr.* **25**, 92–96 (1992).

[71] Gevorkov, Y. et al. XGANDALF—extended gradient descent algorithm for lattice finding. *Acta Crystallogr. A Found. Adv.* **75**, 694–704 (2019). 31475914 10.1107/S2053273319010593 PMC6718201

[72] Evans, P. R. An introduction to data reduction: space-group determination, scaling and intensity statistics. *Acta Crystallogr. D. Biol. Crystallogr.* **67**, 282–292 (2011). 21460446 10.1107/S090744491003982X PMC3069743

[73] Winn, M. D. et al. Overview of the CCP4 suite and current developments. *Acta Crystallogr. D. Biol. Crystallogr* **67**, 235–242 (2011). 21460441 10.1107/S0907444910045749 PMC3069738

[74] Vagin, A. & Teplyakov, A. MOLREP: an automated program for molecular replacement. *J. Appl. Crystallogr.* **30**, 1022–1025 (1997).

[75] Murshudov, G. N. et al. REFMAC5 for the refinement of macromolecular crystal structures. *Acta Crystallogr. D Biol. Crystallogr.* **67**, 355–367 (2011). 21460454 10.1107/S0907444911001314 PMC3069751

[76] Emsley, P., Lohkamp, B., Scott, W. G. & Cowtan, K. Features and development of Coot. *Acta Crystallogr. D Biol. Crystallogr.* **66**, 486–501 (2010). 20383002 10.1107/S0907444910007493 PMC2852313

[77] Adams, P. D. et al. PHENIX: a comprehensive Python-based system for macromolecular structure solution. *Acta Crystallogr. D Biol. Crystallogr.* **66**, 213–221 (2010). 20124702 10.1107/S0907444909052925 PMC2815670

