# Supplementary material for: Minimized sample consumption for time-resolved serial crystallography applied to the redox cycle of human NQO1
Source: Commun Chem. 2026 Jan 29;9:107. doi: 10.1038/s42004-026-01908-9 (PMC12957379; doi:10.1038/s42004-026-01908-9)
Supplement: Supplementary file 1 — Supplementary Material [file 42004_2026_1908_MOESM1_ESM.pdf]

Supplementary Information

Minimized Sample Consumption for Time-Resolved Serial Crystallography Applied to the Redox Cycle of Human NQO1

Diandra Doppler[1,2], Alice Grieco[3], Domin Koh[1,2], Abhik Manna[1,2], Adil Ansari[2,4], Roberto Alvarez[2,4], Konstantinos Karpos[2,4], Hung Le[1,2], Mukul Sonker[1,2], Gihan K. Ketawala[1,2], Samira Mahmud[1,2], Isabel Quereda-Moraleda[3], Sayantee Sen[1,2], Angel L. Pey[5], Romain Letrun[8], Katerina Dörner[8], Jayanath C. P. Koliyadu[8], Raphael de Wijn[8], Johan Bielecki[8], Huijong Han[8], Chan Kim[8], Faisal H. M. Koua[8], Adam Round[8], Abhisakh Sarma[8], Tokushi Sato[8], Christina Schmidt[8], Mohammad Vakili[8,9], Dmitrii Zabelskii[8], Richard Bean[8], Adrian P. Mancuso[8,10], Joachim Schulz[8], Raimund Fromme[1,2], Milagros Medina[11], Thomas D. Grant[12], Petra Fromme[1,2], Richard A. Kirian[2,4], Sabine Botha[2,4], Jose Manuel Martin-Garcia[3]*, Alexandra Ros[1,2]*

1. School of Molecular Sciences, Arizona State University, Tempe, AZ, 85287-1604, USA
2. Center for Applied Structural Discovery, The Biodesign Institute, Arizona State University, Tempe, AZ, 85287-7401, USA
3. Department of Crystallography and Structural Biology, Institute of Physical Chemistry Blas Cabrera, Spanish National Research Council (CSIC), Serrano 119, 28006, Madrid, Spain
4. Department of Physics, Arizona State University, Tempe, AZ, 85287-1504, USA
5. Departamento de Química Física, Unidad de Excelencia en Química Aplicada a Biomedicina y Medioambiente e Instituto de Biotecnología, Universidad de Granada, Av. Fuentenueva s/n, 18071, Granada, Spain
8. European XFEL GmbH, Holzkoppel 4, 22869 Schenefeld, Germany
9. Center for Free-Electron Laser Science CFEL, Deutsches Elektronen-Synchrotron DESY, Notkestr. 85, 22607 Hamburg, Germany
10. Department of Chemistry and Physics, La Trobe Institute for Molecular Science, La Trobe University, Melbourne, Victoria 3086, Australia and Present address: Diamond Light Source Ltd, Harwell Science and Innovation Campus, Diamond House, Didcot, OX11 0DE, UK
11. Department of Biochemistry and Molecular and Cellular Biology, Faculty of Sciences and, Institute for Biocomputation and Physics of Complex Systems (BIFI), University of Zaragoza, 50009 Zaragoza, Spain
12. Department of Structural Biology, Jacobs School of Medicine and Biomedical Sciences, SUNY University at Buffalo, 955 Main St, Buffalo, NY, 14203, USA

Droplet Generator Improvements:

Since our main goal is to achieve a 10 Hz droplet injection that matches the X-ray pulse pattern of the EuXFEL, factors influencing the droplet generation were investigated compared to our previous work.[1, 2] First, SEM imaging was conducted to examine the critical dimensions of the droplet generator, more specifically the wall thickness and shape of the barrier separating the electrode channel from the fluidic channel where droplet generation occurs. **Figure SI-1a** revealed a deformation of the 5 µm thick wall that could potentially influence the effectiveness of the electrical trigger in the droplet generation region. Therefore, in subsequent versions of the droplet generation devices, the wall thickness was doubled to 10 µm. SEM imaging confirmed that the improvement in barrier thickness to 10 µm led to the elimination of the deformation (**Figure SI-1b**).

Additionally, further experimental characterization of the droplet generator geometry indicated that a 100 µm wide aqueous channel was suboptimal for generating 10 Hz droplets. Consequently, the sample and substrate delivery channels were widened to 150 µm matching the dimensions of the oil channel. The newly designed droplet generation devices were named DG300-Y-Mixers as illustrated in **Figure SI-1c-d**. Modifying the channel geometry in the droplet generation region alters the capillary number, thereby influencing the fluid interactions within the transient droplet generation regime between dripping and squeezing.[3, 4] In addition, the taper in *section B* of the DG250-Y-Mixers was eliminated to avoid droplet break up at the junction between the 3D printed part and the capillary. Other elements of *sections A* and *C* in the DG300-Y-Mixers remained unchanged compared to the DG250-Y-Mixers.

Moreover, contact angle studies of the employed resin after printing, as summarized in **Figure SI-1e-f,** revealed that the coating strategy of the walls of the droplet injector could be further improved. Coating the resin surfaces overnight with NOVEC 1720 (3M, St. Paul, USA) followed by thermal curing at 65°C, increased the longevity of the surface treatment when the surface is in contact with the oil phase. Additionally, these surfaces remained stable for several weeks after the treatment and curing when stored in air. **Figure SI-1e** illustrates the decay of the contact angle on a 3D printed surface over a period of up to 6 h of submersion in oil emulating the conditions of use for droplet-generating devices when transporting protein crystal-containing droplets in oil. When the surface treatment was not thermally cured, the observed decay in contact angle was more pronounced and exhibited a steeper decline compared to the surfaces that were thermally cured. This difference suggests that the longevity of the surface treatment is prolonged due to thermal curing and within a droplet generator, this extension in the lifetime of the surface treatment increases sustained functionality throughout an operational shift. The new surface treatment procedure was then implemented and applied to the DG300-Y-Mixers.

Additionally, because the preparation of an SFX beam time requires the fabrication of the mixer devices a few weeks in advance, performing a longevity study of the surface treatment was crucial (**Figure SI-1f**). The sustained contact angle on the thermally cured surfaces confirmed that the devices remained hydrophobic up to two weeks after initial surface treatment, which is advantageous for experiment preparation.

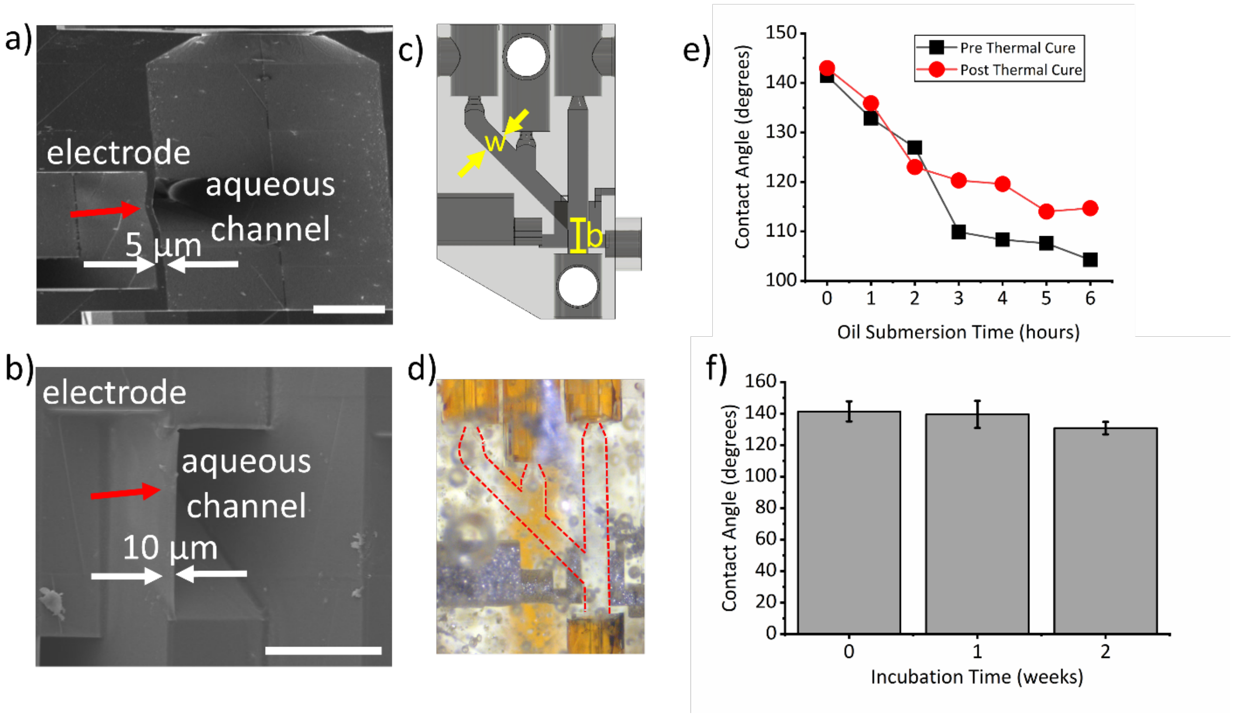

Figure SI-1: Droplet Generator Diagnostic and Improvement. a) *SEM image of the internal structure of the DG300 where a deformation in the 5 µm wall is indicated in red between the aqueous and the electrode channels.* ***b)*** *SEM image of the internal structure of the updated DG300 where the same channel, now 10 µm thick, is indicated in red, but not deformed. Aspect ratios in each SEM image differ and the scale bar represents 200 µm.* ***c)*** *CAD image and* ***d)*** *microscopy image of the DG300-Y-Mixer implemented at the EuXFEL. Contact angle characterization following the previously established procedures for the boundary created between oil, water, and 3D-printed substrate.*[5] ***e)*** *Contact angle decay dependent on the use of thermally cured and not thermally cured surface treatment.* ***f)*** *Contact angle decay after thermal treatment when incubated in air at room temperature.*

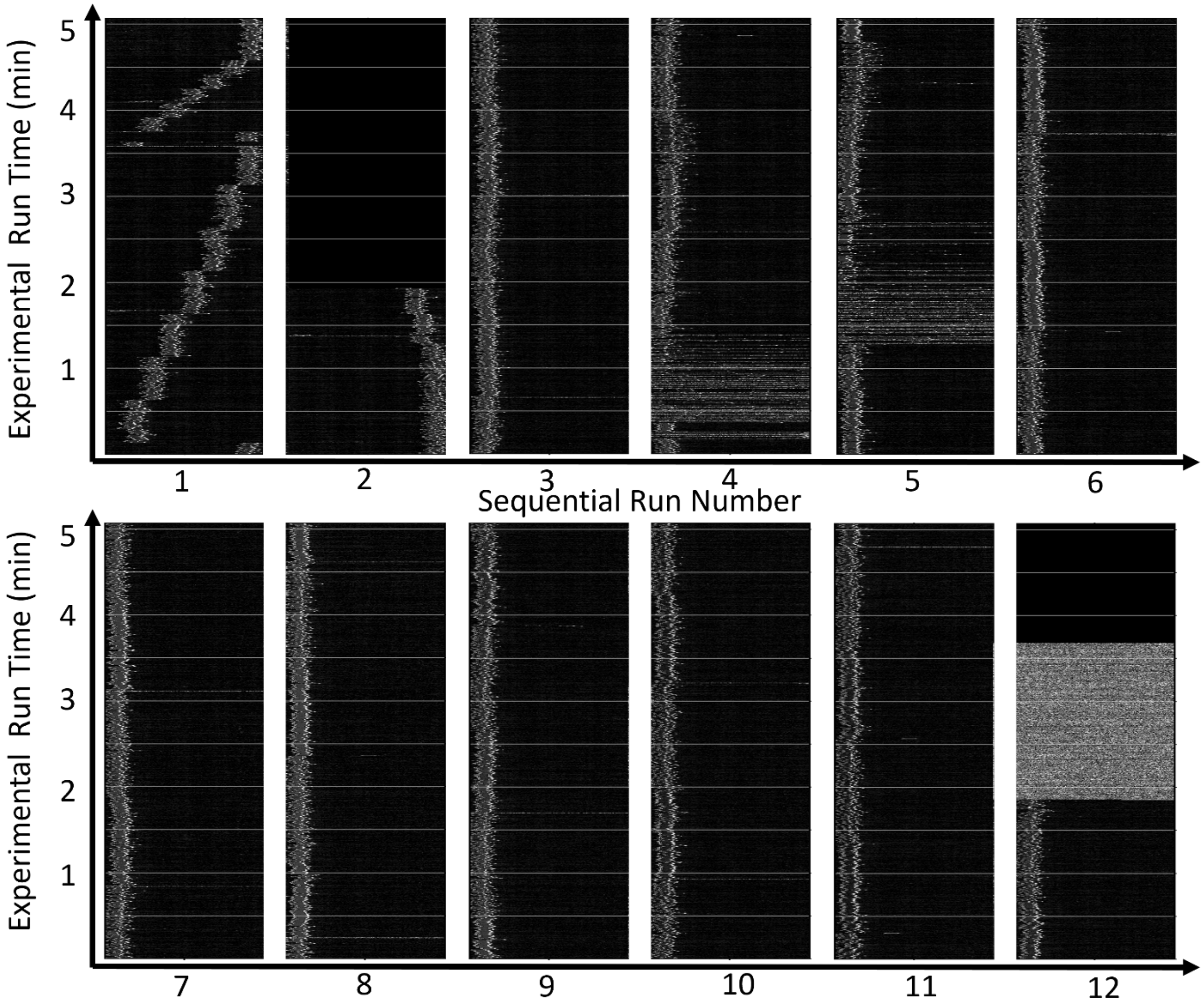

Figure SI-2: Representative Waterfall When Starting at an Experiment. *Waterfall plot collected during P4502 during droplet delivery of mixed NQO1 crystals over 12 runs equating to one hour of continuous data collection. The droplets are initiated and locked-in within the first minute. Subsequently, the droplets are manually scanned along the period of the XFEL in the following 4 min until an optimal delay is obtained. Finally, the droplets remained locked-in at the same delay for 9.5 runs (50 mins) before the droplets were intentionally stopped by turning off the liquid flow and disturbing the droplets around the 2-minute mark of the final run. Due to the low visibility of the droplets in the raw images, contrast (-40%) and brightness (+40%) were adjusted to enhance droplet features. Details on how the waterfall plots are constructed are found in the main manuscript. On each panel, the x-axis originates at 0 ms and extends to 100 ms.*

Supplementary Information for Numerical Modeling:

Table SI-1. Boundary conditions, relevant equations, and modified parameters used for simulations (unless detailed, the parameters remain unchanged from our previous study).[5]

Physics	Boundary Conditions and Modified Parameters
Creeping Flow	Surface Domain:<br>$0 = -\nabla p + \mu \nabla^2 \boldsymbol{u}$<br>$\rho \nabla \cdot (\boldsymbol{u}) = \boldsymbol{0}$<br>**Wall:**<br>$u = 0$ (No slip condition)<br>2D geometry:<br>- Oil channel dimensions: 150×700µm (for DG300-Y-Mixer) and 150×740µm with a taper 114µm from the end of the AQ channel going from 150µm to 100µm (for DG250-Y-Mixer)<br>- The aqueous channel connects to the oil channel at a 45° angle<br>- AQ channel dimensions: 150×730 µm (DG300-Y-Mixer), 100×708 µm (DG250-Y-Mixer)<br>- Crystal stream entry to AQ channel parallel to oil channel and 346 or 380 µm (DG300-Y-Mixer or DG250-Y-Mixer) downstream of the oil channel junction.<br>Inlet: Fully Developed Flow (Flow rate in $m^3 s^{-1}$)<br>Boundary condition = laminar inflow<br>Outlet: $p_0 = 0$
Level Set	Surface Domain (Phase initialization):<br>$\frac{\partial \phi}{\partial t} + \boldsymbol{u} \cdot \boldsymbol{\nabla} \phi = \gamma \nabla \cdot \left(\varepsilon \nabla \phi - \phi(1-\phi) \frac{\partial \phi}{\lvert \nabla \phi \rvert} \right)$<br><br>Initial Value and Inlet for the oil phase ϕ = 0<br>Initial Value and Inlet for the aqueous phase ϕ = 1<br>γ: 0.0146 m s^{-1}<br>η_{oil}: 6.56 cP<br>$\eta_{aqueous}$: 5.39cP<br>ρ_{oil}: 1.8 g m^{-3}<br>$\rho_{aqueous}$: 1 g m^{-3}<br>Surface Tension Coefficient: 14 m N m^{-1}<br>Wetted wall: θ = 2.36 [Rad] for DG 300-Y-Mixer and 2.53 [Rad] for DG250-Y-Mixer.
Transport of Diluted Species	Surface Domain<br>$\nabla \cdot (J_i + \boldsymbol{u} c_i) = R_i$ of species i<br>$J_i = -D_i \nabla c_i$<br>$c_{substrate}$ = 300 mol m^{-3} (0.05 mol m^{-3} for experimental comparison)<br>$D_{substrate}$ = 1.2x10^{-10} m^2 s^{-1} (6.7x10^{-10} m^2 s^{-1} for comparison study)
Nomenclature	ϕ = level set function<br>$\boldsymbol{u}$ = Velocity vector of fluid<br>u = fluid velocity [m/s]<br>p = pressure [Pa]<br>t = time [s]

	μ = dynamic fluidic viscosity [Pa s]<br>c_i = Concentration of species i [mol m^{-3}]<br>c_{sub} = Concentration of substrate [mol m^{-3}]<br>J_i = Relative mass flux vector of species i<br>D_i = Diffusion coefficient of species i<br>D_{sub} = Diffusion coefficient of substrate [m^2 s]<br>γ = Reinitialization Parameter [m s]<br>η_{oil} = Viscosity of oil [cP]<br>$\eta_{aqueous}$ = Viscosity of aqueous sample (crystal + substrate) [cP]<br>$\rho_{aqueous}$ = Density of aqueous sample [g m^{-3}]<br>θ = Contact angle [Rad]

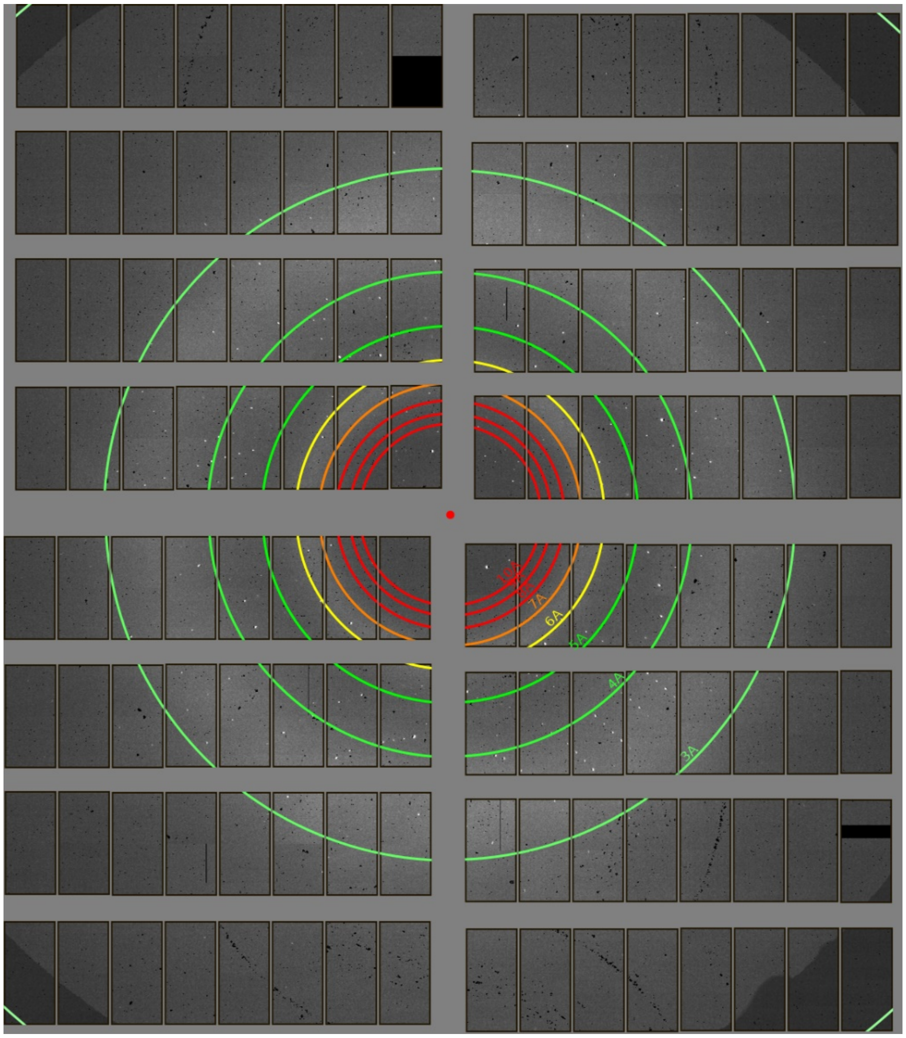

Figure SI-3: A representative diffraction pattern obtained for the 1.2 s time point. The white spots are the recorded Bragg peaks, and bad detector areas and pixels that have been masked out for analysis are shown in black

Table SI-2: Data Collection and Refinement Statistics (values for the outer shell in parentheses)

	Free NQO1 (P3083)	Free NQO1 (P4502)	NQO1-NADH (0.3 s)	NQO1-NADH (1.2 s)
Data collection statistics				
Data collection time (min)	66.5	191.5	38	85.5
Wavelength (Å)	1.3332	1.7712	1.3332	1.7712
Detector	AGIPD 1 Mpx	AGIPD 1 Mpx	AGIPD 1 Mpx	AGIPD 1 Mpx
Space group	$P2_12_12_1$	$P2_12_12_1$	$P2_12_12_1$	$P2_12_12_1$
a, b, c (Å)	61.6, 107.6, 198.6	61.5, 107.8, 198.1	61.6, 107.7, 198.6	61.5, 107.8, 198.1
α, β, γ (°)	90, 90, 90	90, 90, 90	90, 90, 90	90, 90, 90
Resolution range (Å)	27.0-2.5 (2.56-2.50)	26.7-2.30 (2.38-2.30)	25.6-2.5 (2.57-2.51)	26.4-2.5 (2.59-2.50)
Completeness (%)	100 (100)	100 (100)	100 (100)	100 (100)
CC* (%)	98.55 (89.13)	98.59 (71.56)	98.72 (82.0)	97.86 (54.64)
$CC_{1/2}$ (%)	94.39 (65.88)	94.55 (34.41)	95.06 (50.64)	91.86 (17.5)
Multiplicity	489 (347)	237 (142)	287 (204)	115 (84)
R_{split}	20.4 (69.5)	22.5 (126.8)	22.0 (127.1)	28.5 (189.8)
Avg. I/σ (I)	4.3 (1.2)	3.5 (0.6)	3.7 (0.7)	2.8 (0.5)
Refinement Statistics				
Resolution range (Å)	27.0-2.5 (2.56-2.50)	26.7-2-50 (2.56-2.50)	25.6-2.5 (2.57-2.51)	26.4-2.5 (2.56-2.50)
No. of reflections, working set	44,040	44,142	42,064	43,444
No. of reflections, test set	2,350	2,339	2,176	2,326
R_{work}/R_{free} (%)	19.4 / 23.7	17.7 / 22.9	19.7 / 25.5	19.9 / 24.4
No. of non-H atoms				
Protein	8606	8764	8725	8691
Water	278	590	396	436
FAD/NADH	212	212	212 / 212	212
NADH	0	0	132 / 132	88
Others	0	0	0	0
R.m.s. deviations				
Bond length (Å)	0.006	0.006	0.005	0.005

Bond angles (°)	0.001	0.001	0.001	0.001
Average *B* factors (Å^2)	47.00	39.0	57.1	52.5
Ramachandran plot				
Favored (%)	97	98	98	96
Allowed (%)	3	2	2	4
Outliers (%)	0	0	0	0
PDB code	9EZQ	9EZS	9EZR / 9ID0	9EZT

Table SI-3: Data Acquisition During P3083 and P4502

Beam Time	Dataset	Frames	Hits	Indexed Patterns	Lattices
**P3083**	Free NQO1	8,411,372	44,508	35,329	38,226
	NQO1-NADH (0.3 s)	4,422,184	24,631	18,794	19,815
**P4502**	Free NQO1	14,656,518	40,168	28,877	34,367
	NQO1-NADH (1.2 s)	9,490,617	15,268	10,992	12,903

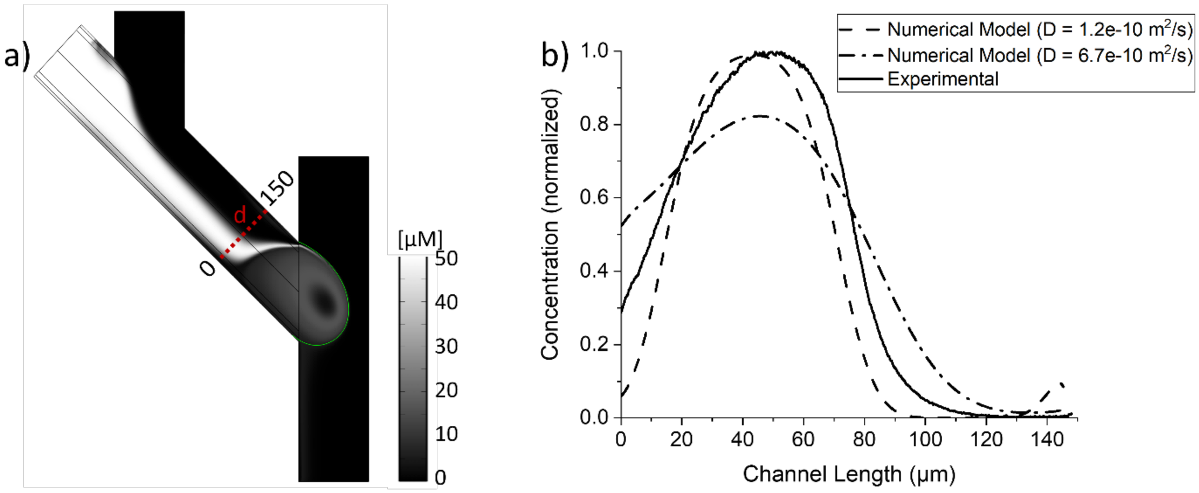

Figure SI-4: Simulation and Experimental Comparison of Substrate Diffusion in the DG300-Y-Mixer. a) *Numerical result of the DG300-Y-Mixer assuming a starting substrate concentration of 50 µM matching the experimental conditions,* where mixing of substrate with the crystals suspension is initiated *highlighting line d (red, dashed), located 187 µm upstream of the start of section B, where diffusion of substrate occurs without influence of flow drift due to droplet generation.* ***b)*** *Comparison of normalized substrate concentration along line d for a diffusion coefficient* of $6.7 * 10^{-6}\ cm^2 s^{-1}$ *as reported by Siritanaratkul et al.*[6]*, the adjusted value of* $1.2 *$

$10^{-6}\ cm^2 s^{-1}$ considering the viscosity of the 18% PEG buffer, and experimental data. For details on the experimental data, see main manuscript.

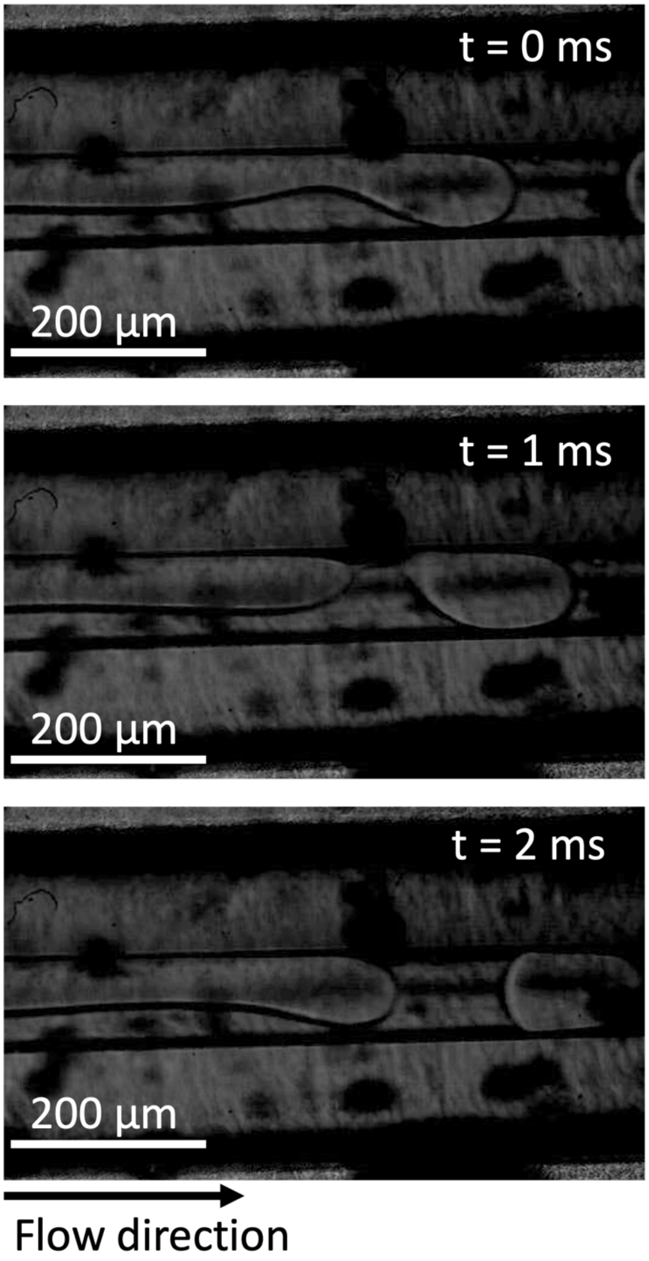

Figure SI-5: Experimental and Simulated Analysis of Substrate Mixing and Equimolarity in the DG250-Y-Mixer. *Microscopy images of droplet pinch off within the silica capillary approximately 2000 µm downstream of start of section C in the DG250-Y-Mixer.*

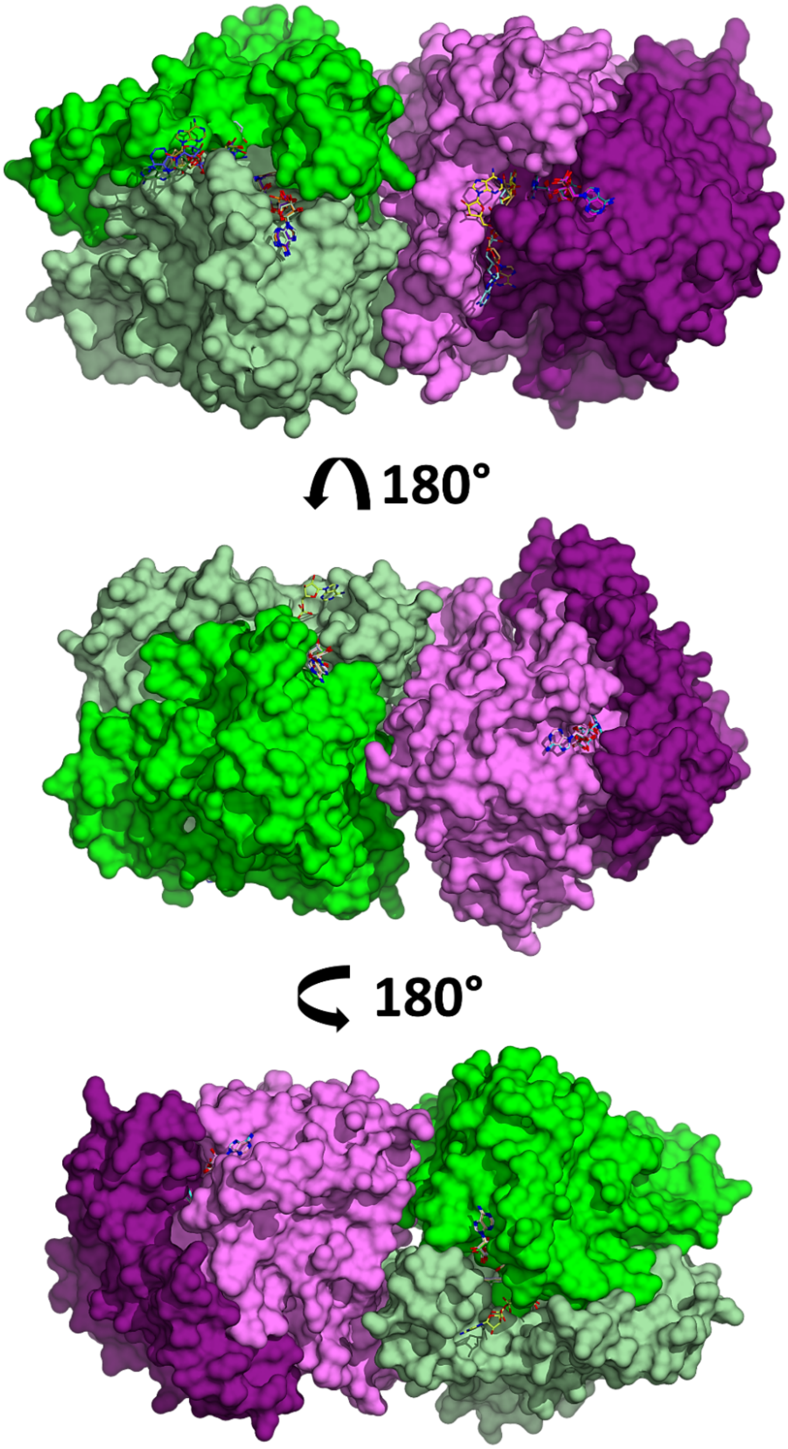

Figure SI-6. Homodimeric assembly of NQO1 in the asymmetric unit. *Surface representation of the two homodimers (one consisting of chains A and B, and the other of chains C and D) present in the asymmetric unit across all structures. The two homodimers in the free NQO1 structure are shown as a reference, with chain A in green, chain B in light green, chain C in violet, and chain D in purple. All FAD and NADH molecules are superimposed onto this structure and are shown as sticks using the same color scheme as in Figures 4 and 5.*

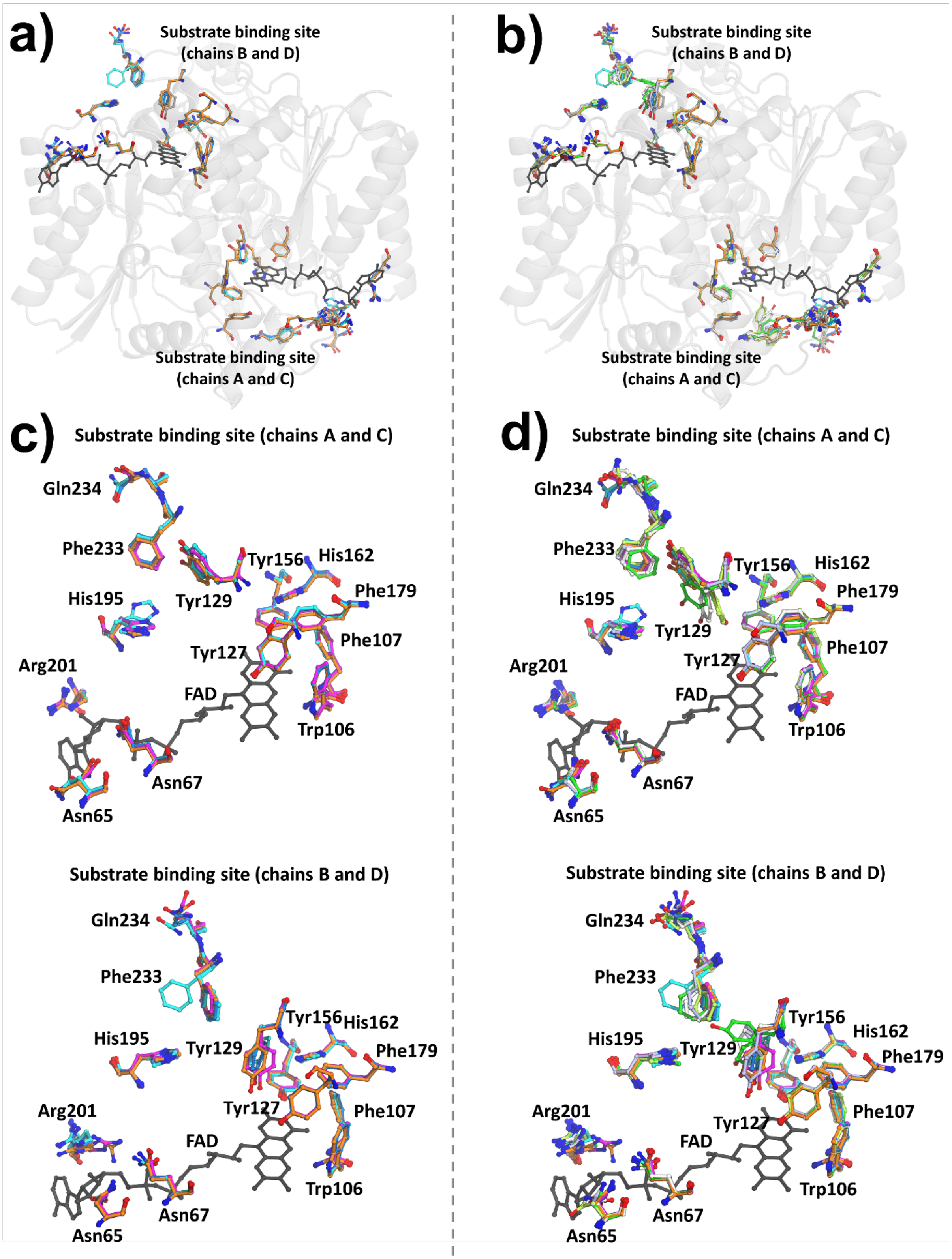

Figure SI-7. Structural comparison of the free NQO1 structures. a) *Superposition of the two homodimers of the free NQO1 structures reported in this study (P3083 (homodimer 1 (magenta)*

and homodimer 2 (blue) and P4502 (homodimer 1 (orange) and homodimer 2 (cyan)). For clarity, only one NQO1 homodimer is shown as a cartoon representation in dark grey and the residues and the FAD in the substrate binding sites of all homodimers are shown as sticks. ***b)*** *Superposition of the two homodimers of the free NQO1 structures reported in this study shown as in a), and those from other free NQO1 structures reported using serial crystallography (homodimer 1 (green) and homodimer 2 (white) for PDB 8C9J (Doppler et al. 2023); homodimer 1 (light blue) and homodimer 2 (limon) for PDB 8RFN (Grieco et al. 2024). For clarity, only one NQO1 homodimer is shown as a cartoon representation in dark grey and the residues and the FAD in the catalytic sites of all homodimers are shown as sticks.* ***c)*** *A closer view of the structural differences observed in the two substrate binding sites shown in a).* ***d)*** *A closer view of the structural differences observed in the two substrate binding sites shown in b).*

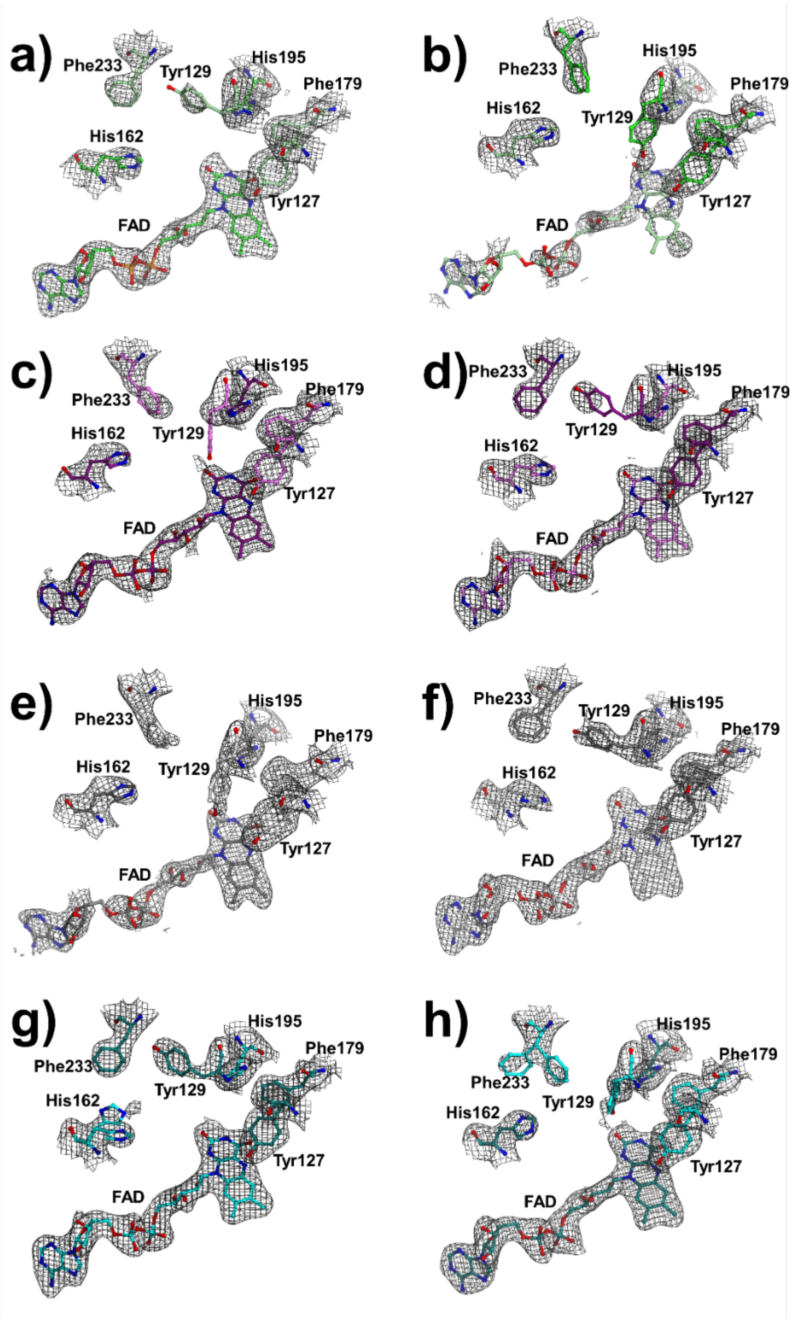

Figure SI-8. Electron density maps 2mFo-DFc for the substrate binding sites of free NQO1 from P3083 and P4502. ***a)*** *Catalytic site of homodimer 1 (chain A) of the free-NQO1 from P3083.* ***b)*** *Substrate binding site of homodimer 1 (chain B) of the free-NQO1 from P3083.* ***c)*** *Substrate binding site of homodimer 2 (chain C) of the free-NQO1 from P3083.* ***d)*** *Substrate binding site of homodimer 2 (chain D) of the free-NQO1 from P3083.* ***e)*** *Substrate binding site of homodimer 1 (chain A) of the free-NQO1 from P4502.* ***f)*** *Substrate binding site of homodimer 1 (chain B) of the free-NQO1 from P4502.* ***g)*** *Substrate binding site of homodimer 2 (chain C) of the free-NQO1 from P4502.* ***h)*** *Substrate binding site of homodimer 2 (chain D) of the free-NQO1 from P4502. All electron density maps have been contoured at 1 σ. All the residues and the FADs are shown in stick representation and using the same color code as in Figures 4 and 5.*

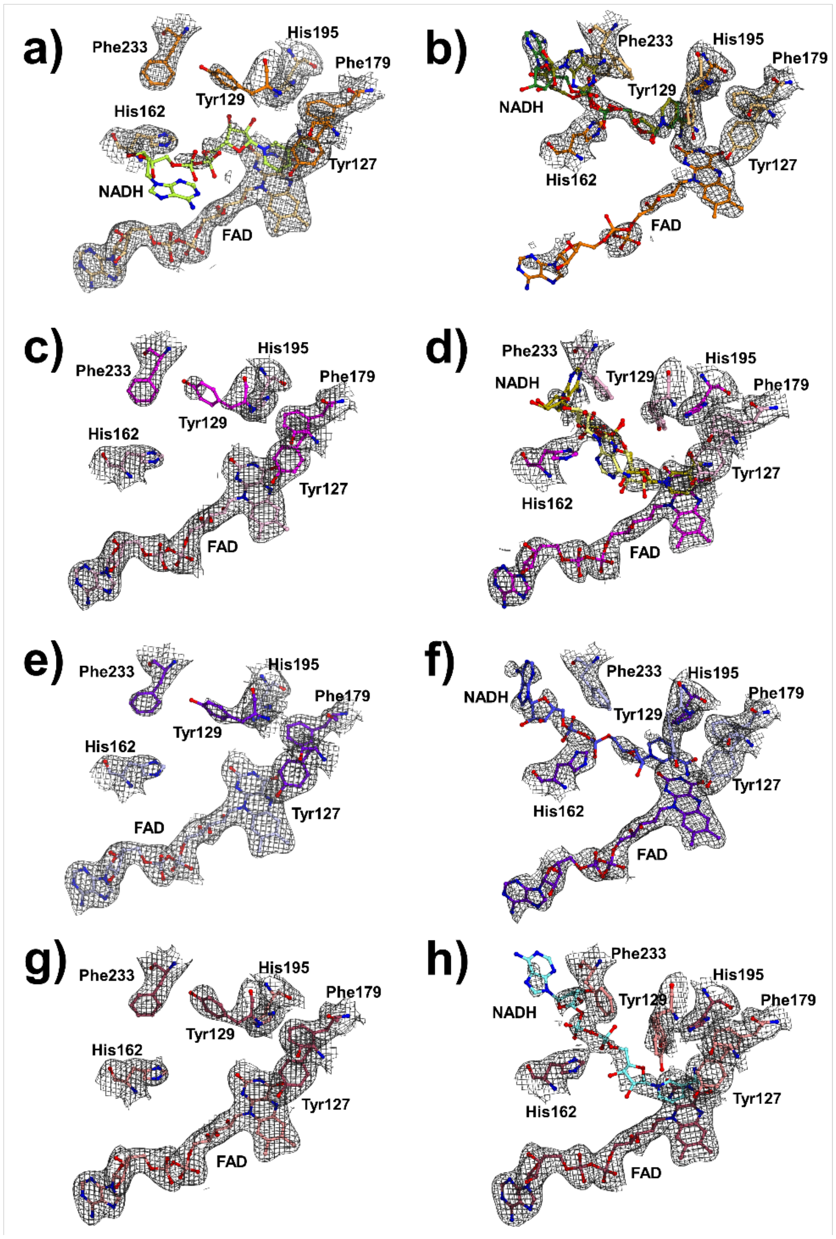

Figure SI-9. Electron density maps 2mFo-DFc for the substrate binding sites of NQO1-NADH at 0.3 and 1.2 s. a) *substrate binding site of homodimer 1 (chain A) of the NQO1-NADH at 0.3 s.* ***b)*** *substrate binding site of homodimer 1 (chain B) of the NQO1-NADH at 0.3 s.* ***c)*** *substrate binding site of homodimer 2 (chain C) of the NQO1-NADH at 0.3 s.* ***d)*** *substrate binding site of homodimer 2 (chain D) of the NQO1-NADH at 0.3 s.* ***e)*** *substrate binding site of homodimer 1 (chain A) of the NQO1-NADH at 1.2 s.* ***f)*** *substrate binding site of homodimer 1 (chain B) of the NQO1-NADH at 1.2 s.* ***g)*** *substrate binding site of homodimer 2 (chain C) of the NQO1-NADH at 1.2 s.* ***h)*** *substrate binding site of homodimer 2 (chain D) of the NQO1-NADH at 1.2 s. All electron density maps around the FADs and the substrate binding site residues have been contoured at 1 σ and the electron density maps around the NADHs have been contoured at 0.8 σ. All the residues, the FAD and the NADH are shown in stick representation and using the same color code as in Figures 4 and 5.*

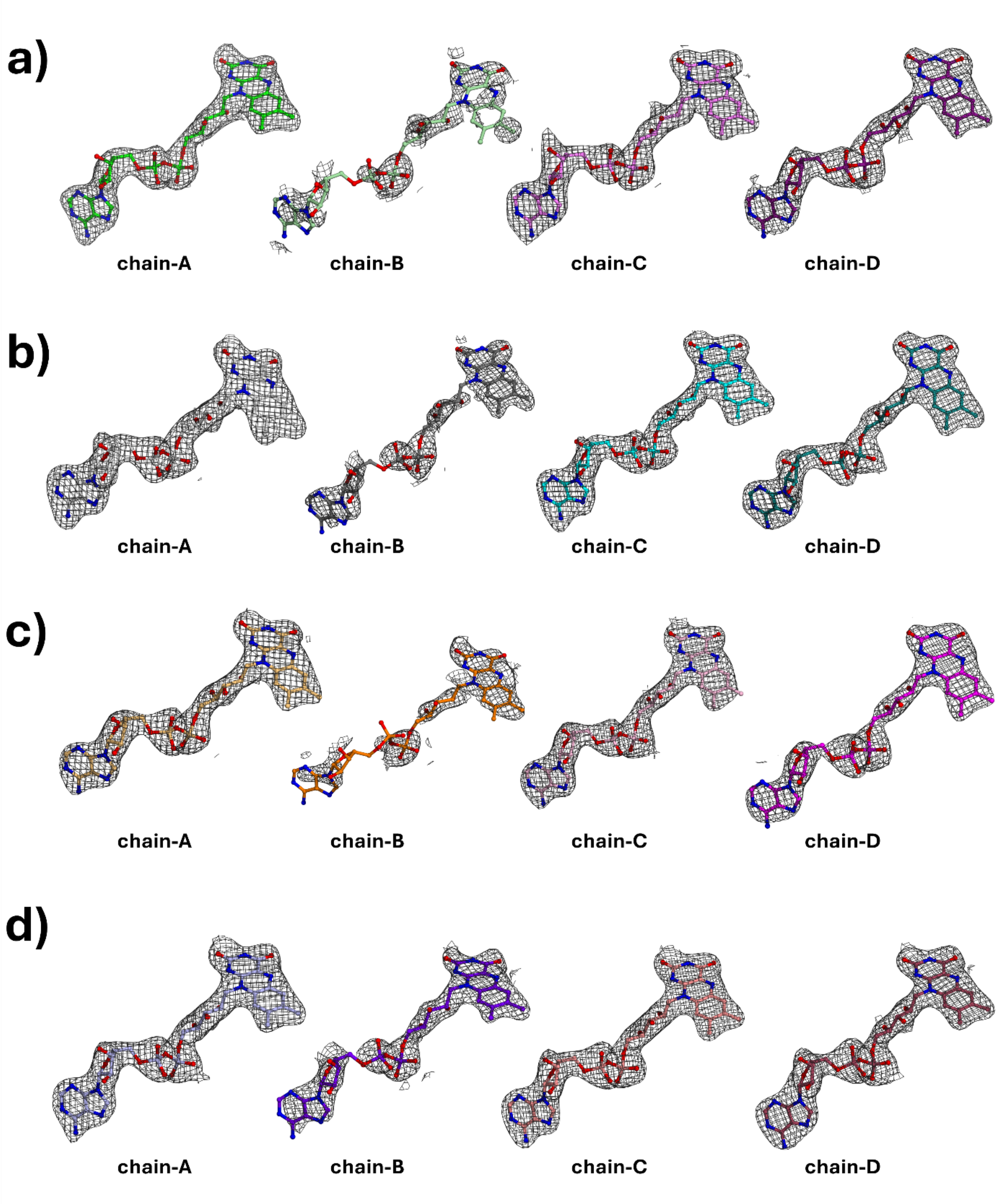

Figure SI-10. Electron density maps 2mFo-DFc of FADs. *a) Electron density maps of all FADs of the free NQO1 from P3083. b) Electron density maps of all FADs of the free NQO1 from P4502. c) Electron density maps of all FADs of the NQO1-NADH at 0.3s. d) Electron density maps of all FADs of the NQO1-NADH at 1.2 s. All electron density maps have been contoured at 1 σ. All FADs are shown in stick representation and using the same color code as in Figures 4 and 5.*

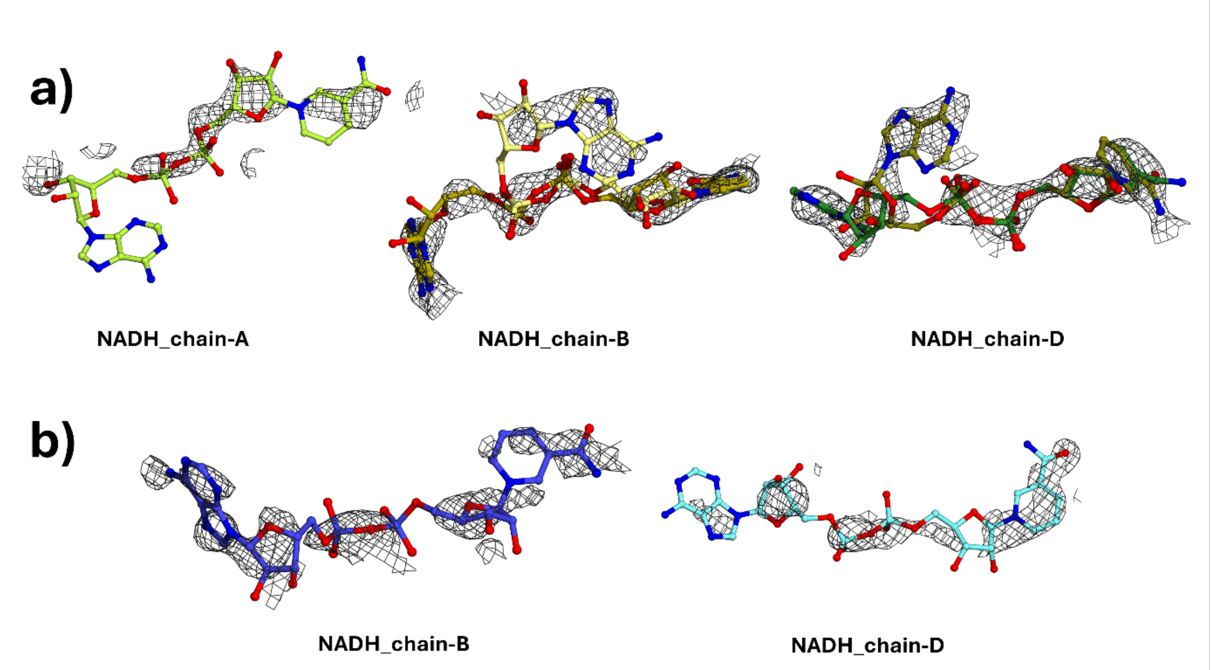

Figure SI-11. Electron density maps 2mFo-DFc of NADHs. *a) Electron density maps of the NADHs found in homodimer 1 (chains A and B) and homodimer 2 (chains D) of the NQO1-NADH at 0.3 s. b) Electron density maps of the NADHs found in homodimer 1 (chain B) and homodimer 2 (chain D) of the NQO1-NADH at 1.2 s. All electron density maps have been contoured at 0.8 σ. The NADHs are shown in stick representation and using the same color code as in Figures 4 and 5.*

References

(1) Doppler, D.; Sonker, M.; Egatz-Gomez, A.; Grieco, A.; Zaare, S.; Jernigan, R.; Meza-Aguilar, J. D.; Rabbani, M. T.; Manna, A.; Alvarez, R. C.; et al. Modular droplet injector for sample conservation providing new structural insight for the conformational heterogeneity in the disease-associated NQO1 enzyme. *Lab Chip* **2023**, *23* (13), 3016-3033. DOI: 10.1039/d3lc00176h.
(2) Sonker, M.; Doppler, D.; Egatz-Gomez, A.; Zaare, S.; Rabbani, M. T.; Manna, A.; Cruz Villarreal, J.; Nelson, G.; Ketawala, G. K.; Karpos, K.; et al. Electrically stimulated droplet injector for reduced sample consumption in serial crystallography. *Biophys Rep* **2022**, *2* (4), 100081. DOI: 10.1016/j.bpr.2022.100081.
(3) Echelmeier, A.; Cruz Villarreal, J.; Messerschmidt, M.; Kim, D.; Coe, J. D.; Thifault, D.; Botha, S.; Egatz-Gomez, A.; Gandhi, S.; Brehm, G.; et al. Segmented flow generator for serial crystallography at the European X-ray free electron laser. *Nat Commun* **2020**, *11* (1), 4511. DOI: 10.1038/s41467-020-18156-7.
(4) Xu, J. H.; Li, S. W.; Tan, J.; Luo, G. S. Correlations of droplet formation in T-junction microfluidic devices: from squeezing to dripping. *Microfluidics and Nanofluidics* **2008**, *5* (6), 711-717. DOI: 10.1007/s10404-008-0306-4.
(5) Doppler, D.; Rabbani, M. T.; Letrun, R.; Cruz Villarreal, J.; Kim, D. H.; Gandhi, S.; Egatz-Gomez, A.; Sonker, M.; Chen, J.; Koua, F. H. M.; et al. Co-flow injection for serial crystallography at X-ray free-electron lasers. *J Appl Crystallogr* **2022**, *55* (Pt 1), 1-13. DOI: 10.1107/S1600576721011079.
(6) Siritanaratkul, B.; Megarity, C. F.; Roberts, T. G.; Samuels, T. O. M.; Winkler, M.; Warner, J. H.; Happe, T.; Armstrong, F. A. Transfer of photosynthetic NADP(+)/NADPH recycling activity to a porous metal oxide for highly specific, electrochemically-driven organic synthesis. *Chem Sci* **2017**, *8* (6), 4579-4586. DOI: 10.1039/c7sc00850c.
