# Supplementary material for: Minimized sample consumption for time-resolved serial crystallography applied to the redox cycle of human NQO1
Source: Commun Chem. 2026 Jan 29;9:107. doi: 10.1038/s42004-026-01908-9 (PMC12957379; doi:10.1038/s42004-026-01908-9)
Supplement: Supplementary file 2 — Description of Additional Supplementary Files [file 42004_2026_1908_MOESM2_ESM.pdf]

Description of Additional Supplementary Files:

File name: Supplementary Data 1

Description: Contains Source Data for Figure 3 d and h

File name: Supplementary Data 2

Description: pdb file for structure *9EZQ*

File name: Supplementary Data 3

Description: pdb file for structure *9EZR*

File name: Supplementary Data 4

Description: pdb file for structure *9EZS*

File name: Supplementary Data 5

Description: pdb file for structure *9EZT*

File name: Supplementary Data 6

Description: pdb file for structure *9ID0*
