# Supplementary material for: Minimized sample consumption for time-resolved serial crystallography applied to the redox cycle of human NQO1
Source: Commun Chem. 2026 Jan 29;9:107. doi: 10.1038/s42004-026-01908-9 (PMC12957379; doi:10.1038/s42004-026-01908-9)
Supplement: Supplementary file 7 — supplementary data 5 [file 42004_2026_1908_MOESM7_ESM.pdf]

Full wwPDB X-ray Structure Validation Report (i)

Apr 8, 2025 – 11:19 am BST

PDB ID	:	9EZT / pdb_00009ezt
Title	:	XFEL structure of hNQO1 mixed with NADH for 1.2 s
Deposited on	:	2024-04-14
Resolution	:	2.50 Å(reported)

This wwPDB validation report is for manuscript review

For Manuscript Review

This is a Full wwPDB X-ray Structure Validation Report.

This report is produced by the wwPDB biocuration pipeline after annotation of the structure.

We welcome your comments at *validation@mail.wwpdb.org*
A user guide is available at
https://www.wwpdb.org/validation/2017/XrayValidationReportHelp
with specific help available everywhere you see the (i) symbol.

The types of validation reports are described at
http://www.wwpdb.org/validation/2017/FAQs#types.

The following versions of software and data (see references (i)) were used in the production of this report:

MolProbity	:	4.02b-467
Mogul	:	1.8.4, CSD as541be (2020)
Xtriage (Phenix)	:	1.13
EDS	:	3.0
buster-report	:	1.1.7 (2018)
Percentile statistics	:	20231227.v01 (using entries in the PDB archive December 27th 2023)
CCP4	:	9.0.003 (Gargrove)
Density-Fitness	:	1.0.11
Ideal geometry (proteins)	:	Engh & Huber (2001)
Ideal geometry (DNA, RNA)	:	Parkinson et al. (1996)

1 Overall quality at a glance (i)

The following experimental techniques were used to determine the structure:
X-RAY DIFFRACTION

The reported resolution of this entry is 2.50 Å.

Percentile scores (ranging between 0-100) for global validation metrics of the entry are shown in the following graphic. The table shows the number of entries on which the scores are based.

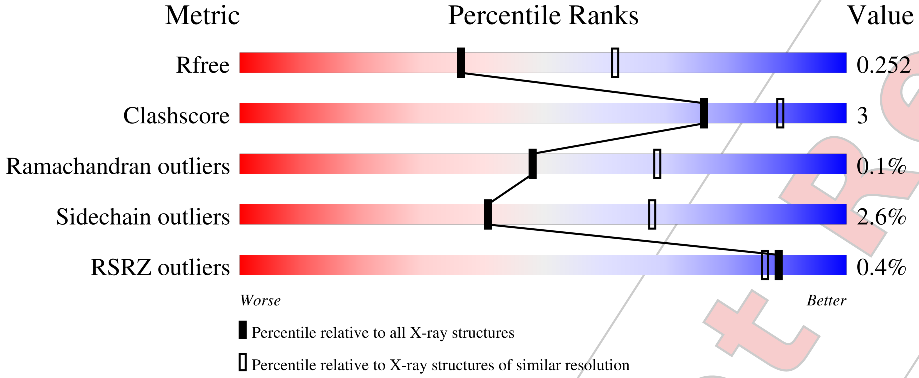

Metric	Whole archive (#Entries)	Similar resolution (#Entries, resolution range(Å))
R_{free}	164625	5504 (2.50-2.50)
Clashscore	180529	6282 (2.50-2.50)
Ramachandran outliers	177936	6191 (2.50-2.50)
Sidechain outliers	177891	6193 (2.50-2.50)
RSRZ outliers	164620	5504 (2.50-2.50)

The table below summarises the geometric issues observed across the polymeric chains and their fit to the electron density. The red, orange, yellow and green segments of the lower bar indicate the fraction of residues that contain outliers for >=3, 2, 1 and 0 types of geometric quality criteria respectively. A grey segment represents the fraction of residues that are not modelled. The numeric value for each fraction is indicated below the corresponding segment, with a dot representing fractions <=5% The upper red bar (where present) indicates the fraction of residues that have poor fit to the electron density. The numeric value is given above the bar.

Mol	Chain	Length	Quality of chain
1	A	274	94% 5% •
1	B	274	91% 8% •
1	D	274	92% 6% •
2	C	279	% 94% 5% •

Validation Pipeline (wwPDB-VP) : 2.42

For Manuscript Review

2 Entry composition (i)

There are 5 unique types of molecules in this entry. The entry contains 9550 atoms, of which 0 are hydrogens and 0 are deuteriums.

In the tables below, the ZeroOcc column contains the number of atoms modelled with zero occupancy, the AltConf column contains the number of residues with at least one atom in alternate conformation and the Trace column contains the number of residues modelled with at most 2 atoms.

- Molecule 1 is a protein called NAD(P)H dehydrogenase [quinone] 1.

Mol	Chain	Residues	Atoms					ZeroOcc	AltConf	Trace
1	A	274	Total 2226	C 1443	N 377	O 399	S 7	0	5	0
1	B	271	Total 2177	C 1414	N 366	O 390	S 7	0	2	0
1	D	273	Total 2192	C 1426	N 368	O 391	S 7	0	2	0

- Molecule 2 is a protein called NAD(P)H dehydrogenase [quinone] 1.

Mol	Chain	Residues	Atoms					ZeroOcc	AltConf	Trace
2	C	279	Total 2219	C 1443	N 372	O 396	S 8	0	1	0

There are 5 discrepancies between the modelled and reference sequences:

Chain	Residue	Modelled	Actual	Comment	Reference
C	-4	ALA	-	expression tag	UNP P15559
C	-3	ALA	-	expression tag	UNP P15559
C	-2	ALA	-	expression tag	UNP P15559
C	-1	ALA	-	expression tag	UNP P15559
C	0	ALA	-	expression tag	UNP P15559

- Molecule 3 is FLAVIN-ADENINE DINUCLEOTIDE (CCD ID: FAD) (formula: $C_{27}H_{33}N_9O_{15}P_2$) (labeled as "Ligand of Interest" by depositor).

For Manuscript Review

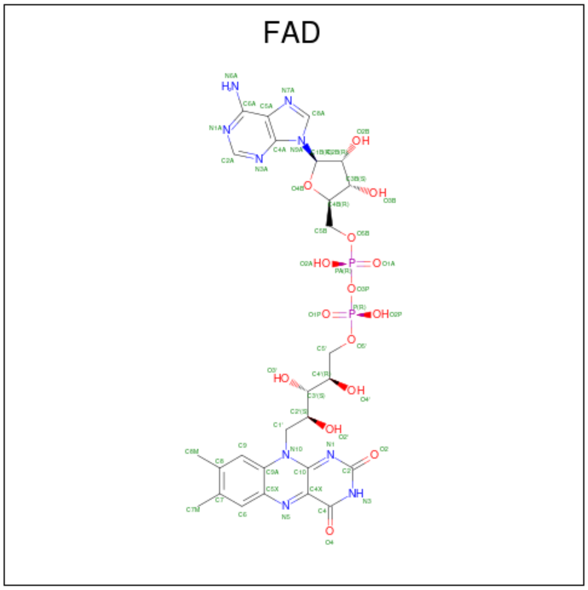

Mol	Chain	Residues	Atoms	ZeroOcc	AltConf
3	A	1	Total C N O P<br>53 27 9 15 2	0	0
3	B	1	Total C N O P<br>53 27 9 15 2	0	0
3	C	1	Total C N O P<br>53 27 9 15 2	0	0
3	D	1	Total C N O P<br>53 27 9 15 2	0	0

- Molecule 4 is NICOTINAMIDE-ADENINE-DINUCLEOTIDE (CCD ID: NAD) (formula: $C_{21}H_{27}N_7O_{14}P_2$).

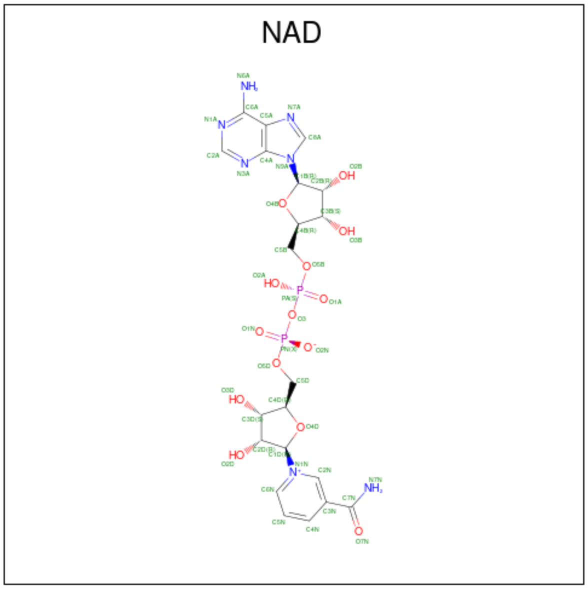

For Manuscript Review

Mol	Chain	Residues	Atoms					ZeroOcc	AltConf
4	B	1	Total 44	C 21	N 7	O 14	P 2	0	0
4	C	1	Total 44	C 21	N 7	O 14	P 2	0	0

- Molecule 5 is water.

Mol	Chain	Residues	Atoms		ZeroOcc	AltConf
5	A	109	Total 109	O 109	0	0
5	B	120	Total 120	O 120	0	0
5	C	107	Total 107	O 107	0	0
5	D	100	Total 100	O 100	0	0

For Manuscript Review

3 Residue-property plots ⓘ

These plots are drawn for all protein, RNA, DNA and oligosaccharide chains in the entry. The first graphic for a chain summarises the proportions of the various outlier classes displayed in the second graphic. The second graphic shows the sequence view annotated by issues in geometry and electron density. Residues are color-coded according to the number of geometric quality criteria for which they contain at least one outlier: green = 0, yellow = 1, orange = 2 and red = 3 or more. A red dot above a residue indicates a poor fit to the electron density (RSRZ > 2). Stretches of 2 or more consecutive residues without any outlier are shown as a green connector. Residues present in the sample, but not in the model, are shown in grey.

- Molecule 1: NAD(P)H dehydrogenase [quinone] 1

Chain A:

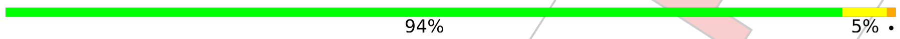

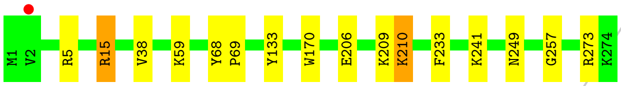

- Molecule 1: NAD(P)H dehydrogenase [quinone] 1

Chain B:

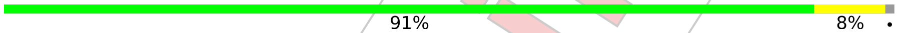

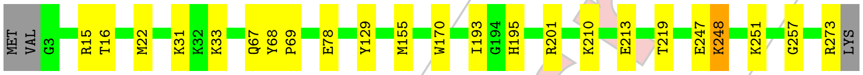

- Molecule 1: NAD(P)H dehydrogenase [quinone] 1

Chain D:

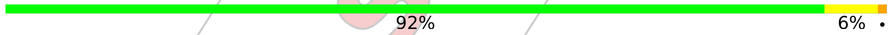

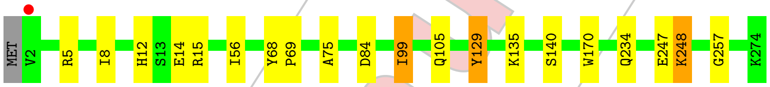

- Molecule 2: NAD(P)H dehydrogenase [quinone] 1

Chain C:

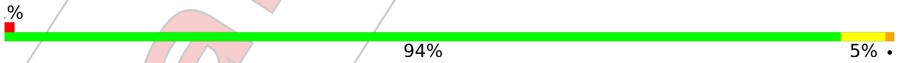

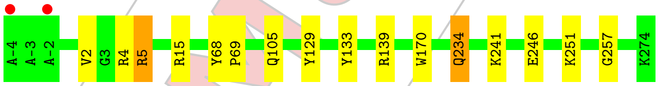

For Manuscript Review

4 Data and refinement statistics (i)

Property	Value	Source
Space group	P 21 21 21	Depositor
Cell constants<br>a, b, c, α, β, γ	61.50Å 107.80Å 198.10Å<br>90.00° 90.00° 90.00°	Depositor
Resolution (Å)	26.47 – 2.50<br>26.47 – 2.50	Depositor<br>EDS
% Data completeness<br>(in resolution range)	98.3 (26.47-2.50)<br>98.3 (26.47-2.50)	Depositor<br>EDS
R_{merge}	(Not available)	Depositor
R_{sym}	(Not available)	Depositor
$< I/\sigma(I) >$ [1]	1.00 (at 2.50Å)	Xtriage
Refinement program	REFMAC 5.8.0430	Depositor
R, R_{free}	0.192 , 0.250<br>0.199 , 0.252	Depositor<br>DCC
R_{free} test set	2351 reflections (5.08%)	wwPDB-VP
Wilson B-factor (Å^2)	53.0	Xtriage
Anisotropy	0.030	Xtriage
Bulk solvent k_{sol}(e/Å^3), B_{sol}(Å^2)	0.32 , 41.3	EDS
L-test for twinning[2]	$< \|L\| > = 0.47$, $< L^2 > = 0.30$	Xtriage
Estimated twinning fraction	No twinning to report.	Xtriage
F_o,F_c correlation	0.94	EDS
Total number of atoms	9550	wwPDB-VP
Average B, all atoms (Å^2)	54.0	wwPDB-VP

Xtriage's analysis on translational NCS is as follows: *The largest off-origin peak in the Patterson function is 3.04% of the height of the origin peak. No significant pseudotranslation is detected.*

[1] Intensities estimated from amplitudes.

[2] Theoretical values of $< |L| >$, $< L^2 >$ for acentric reflections are 0.5, 0.333 respectively for untwinned datasets, and 0.375, 0.2 for perfectly twinned datasets.

For Manuscript Review

5 Model quality (i)

5.1 Standard geometry (i)

Bond lengths and bond angles in the following residue types are not validated in this section: NAD, FAD

The Z score for a bond length (or angle) is the number of standard deviations the observed value is removed from the expected value. A bond length (or angle) with $|Z| > 5$ is considered an outlier worth inspection. RMSZ is the root-mean-square of all Z scores of the bond lengths (or angles).

Mol	Chain	Bond lengths		Bond angles	
		RMSZ	$\#\|Z\|>5$	RMSZ	$\#\|Z\|>5$
1	A	0.36	0/2285	0.71	2/3086 (0.1%)
1	B	0.36	0/2236	0.68	1/3021 (0.0%)
1	D	0.35	0/2251	0.68	1/3040 (0.0%)
2	C	0.36	0/2278	0.70	1/3078 (0.0%)
All	All	0.36	0/9050	0.69	5/12225 (0.0%)

Chiral center outliers are detected by calculating the chiral volume of a chiral center and verifying if the center is modelled as a planar moiety or with the opposite hand.A planarity outlier is detected by checking planarity of atoms in a peptide group, atoms in a mainchain group or atoms of a sidechain that are expected to be planar.

Mol	Chain	#Chirality outliers	#Planarity outliers
1	D	0	1
2	C	0	1
All	All	0	2

There are no bond length outliers.

All (5) bond angle outliers are listed below:

Mol	Chain	Res	Type	Atoms	Z	Observed(^{o})	Ideal(^{o})
1	A	15	ARG	CG-CD-NE	5.95	124.29	111.80
2	C	133	TYR	CB-CA-C	5.28	120.95	110.40
1	D	129	TYR	N-CA-CB	-5.27	101.11	110.60
1	A	133	TYR	CB-CA-C	5.24	120.87	110.40
1	B	22	MET	CG-SD-CE	5.03	108.25	100.20

There are no chirality outliers.

All (2) planarity outliers are listed below:

For Manuscript Review

Mol	Chain	Res	Type	Group
2	C	4	ARG	Sidechain
1	D	5	ARG	Sidechain

5.2 Too-close contacts (i)

In the following table, the Non-H and H(model) columns list the number of non-hydrogen atoms and hydrogen atoms in the chain respectively. The H(added) column lists the number of hydrogen atoms added and optimized by MolProbity. The Clashes column lists the number of clashes within the asymmetric unit, whereas Symm-Clashes lists symmetry-related clashes.

Mol	Chain	Non-H	H(model)	H(added)	Clashes	Symm-Clashes
1	A	2226	0	2214	9	0
1	B	2177	0	2165	17	0
1	D	2192	0	2184	13	0
2	C	2219	0	2221	9	0
3	A	53	0	31	0	0
3	B	53	0	31	3	0
3	C	53	0	31	1	0
3	D	53	0	31	2	0
4	B	44	0	26	7	0
4	C	44	0	26	3	0
5	A	109	0	0	4	1
5	B	120	0	0	7	1
5	C	107	0	0	1	0
5	D	100	0	0	4	0
All	All	9550	0	8960	51	1

The all-atom clashscore is defined as the number of clashes found per 1000 atoms (including hydrogen atoms). The all-atom clashscore for this structure is 3.

All (51) close contacts within the same asymmetric unit are listed below, sorted by their clash magnitude.

Atom-1	Atom-2	Interatomic distance (Å)	Clash overlap (Å)
4:B:302:NAD:O3D	5:B:401:HOH:O	1.60	1.18
4:B:302:NAD:N7A	5:B:402:HOH:O	1.91	1.00
1:B:273:ARG:NH1	5:B:404:HOH:O	2.11	0.82
1:A:5[B]:ARG:HH21	1:A:5[B]:ARG:HB3	1.46	0.81
1:A:206[A]:GLU:OE2	5:A:401:HOH:O	2.02	0.77
1:D:12:HIS:HE1	3:D:301:FAD:O2P	1.72	0.72
1:B:213:GLU:OE2	5:B:403:HOH:O	2.08	0.70
1:A:210:LYS:HE3	5:A:438:HOH:O	1.99	0.62

Continued on next page...

For Manuscript Review

Continued from previous page...

Atom-1	Atom-2	Interatomic distance (Å)	Clash overlap (Å)
2:C:234:GLN:HG2	5:C:474:HOH:O	1.99	0.61
1:B:213:GLU:CG	5:B:403:HOH:O	2.50	0.58
2:C:129[B]:TYR:OH	4:C:302:NAD:N1N	2.38	0.57
2:C:5:ARG:HH11	2:C:5:ARG:HB3	1.72	0.55
1:D:234[A]:GLN:NE2	5:D:405:HOH:O	2.39	0.55
1:B:193:ILE:O	1:B:201:ARG:NH2	2.41	0.54
1:A:209:LYS:HD3	5:A:401:HOH:O	2.07	0.54
1:B:213:GLU:HG2	5:B:403:HOH:O	2.06	0.54
4:C:302:NAD:H2B	4:C:302:NAD:N3A	2.26	0.50
1:A:59:LYS:NZ	5:A:406:HOH:O	2.44	0.50
1:D:135:LYS:HD3	5:D:469:HOH:O	2.11	0.50
1:A:233:PHE:HZ	4:B:302:NAD:H52N	1.76	0.49
1:D:8:ILE:HG12	1:D:99:ILE:HG23	1.95	0.49
1:B:247:GLU:O	1:B:248:LYS:C	2.53	0.47
1:B:201:ARG:CZ	3:B:301:FAD:H4B	2.44	0.46
1:A:5[B]:ARG:HH22	1:A:38:VAL:HG23	1.80	0.46
1:B:195:HIS:HD1	4:B:302:NAD:PA	2.39	0.46
1:D:8:ILE:HG12	1:D:99:ILE:CG2	2.46	0.46
1:B:67:GLN:HB3	5:B:475:HOH:O	2.16	0.46
1:B:155:MET:CE	4:B:302:NAD:H5N	2.46	0.46
2:C:170:TRP:CZ2	2:C:257:GLY:HA3	2.51	0.45
1:A:170:TRP:CZ2	1:A:257:GLY:HA3	2.51	0.45
1:B:195:HIS:HE1	4:B:302:NAD:H51N	1.82	0.45
1:D:84:ASP:OD2	5:D:401:HOH:O	2.21	0.45
1:D:170:TRP:CZ2	1:D:257:GLY:HA3	2.50	0.45
1:D:56:ILE:HD12	1:D:75:ALA:HB2	1.98	0.45
1:B:170:TRP:CZ2	1:B:257:GLY:HA3	2.52	0.45
1:B:201:ARG:NH2	3:B:301:FAD:C5B	2.80	0.45
2:C:129[B]:TYR:OH	4:C:302:NAD:C2N	2.66	0.44
2:C:5:ARG:HB3	2:C:5:ARG:NH1	2.32	0.44
1:B:68:TYR:N	1:B:69:PRO:CD	2.81	0.44
1:D:135:LYS:HG3	5:D:425:HOH:O	2.18	0.43
1:A:68:TYR:N	1:A:69:PRO:CD	2.81	0.43
1:B:16:THR:O	3:B:301:FAD:H2B	2.19	0.43
1:D:68:TYR:N	1:D:69:PRO:CD	2.81	0.43
2:C:68:TYR:N	2:C:69:PRO:CD	2.83	0.42
1:B:210:LYS:HD2	1:B:210:LYS:HA	1.87	0.42
1:B:195:HIS:CE1	4:B:302:NAD:H51N	2.55	0.41
1:D:12:HIS:HD2	1:D:14:GLU:H	1.69	0.41
2:C:246:GLU:OE1	2:C:246:GLU:HA	2.21	0.41
1:D:247:GLU:O	1:D:248:LYS:C	2.60	0.40

Continued on next page...

For Manuscript Review

Continued from previous page...

Atom-1	Atom-2	Interatomic distance (Å)	Clash overlap (Å)
2:C:105:GLN:HA	3:C:301:FAD:C5X	2.52	0.40
1:D:105:GLN:HA	3:D:301:FAD:C5X	2.51	0.40

All (1) symmetry-related close contacts are listed below. The label for Atom-2 includes the symmetry operator and encoded unit-cell translations to be applied.

Atom-1	Atom-2	Interatomic distance (Å)	Clash overlap (Å)
5:A:506:HOH:O	5:B:517:HOH:O[3_445]	1.33	0.87

5.3 Torsion angles (i)

5.3.1 Protein backbone (i)

In the following table, the Percentiles column shows the percent Ramachandran outliers of the chain as a percentile score with respect to all X-ray entries followed by that with respect to entries of similar resolution.

The Analysed column shows the number of residues for which the backbone conformation was analysed, and the total number of residues.

Mol	Chain	Analysed	Favoured	Allowed	Outliers	Percentiles	
1	A	277/274 (101%)	266 (96%)	11 (4%)	0	100	100
1	B	271/274 (99%)	259 (96%)	11 (4%)	1 (0%)	30	49
1	D	273/274 (100%)	263 (96%)	10 (4%)	0	100	100
2	C	278/279 (100%)	268 (96%)	10 (4%)	0	100	100
All	All	1099/1101 (100%)	1056 (96%)	42 (4%)	1 (0%)	48	69

All (1) Ramachandran outliers are listed below:

Mol	Chain	Res	Type
1	B	129	TYR

5.3.2 Protein sidechains (i)

In the following table, the Percentiles column shows the percent sidechain outliers of the chain as a percentile score with respect to all X-ray entries followed by that with respect to entries of similar resolution.

For Manuscript Review

The Analysed column shows the number of residues for which the sidechain conformation was analysed, and the total number of residues.

Mol	Chain	Analysed	Rotameric	Outliers	Percentiles	
1	A	232/228 (102%)	227 (98%)	5 (2%)	47	73
1	B	227/228 (100%)	220 (97%)	7 (3%)	35	62
1	D	228/228 (100%)	223 (98%)	5 (2%)	47	73
2	C	229/228 (100%)	222 (97%)	7 (3%)	35	62
All	All	916/912 (100%)	892 (97%)	24 (3%)	41	68

All (24) residues with a non-rotameric sidechain are listed below:

Mol	Chain	Res	Type
1	A	15	ARG
1	A	210	LYS
1	A	241	LYS
1	A	249	ASN
1	A	273	ARG
1	B	15	ARG
1	B	31	LYS
1	B	33	LYS
1	B	78	GLU
1	B	219	THR
1	B	248	LYS
1	B	251	LYS
2	C	2	VAL
2	C	5	ARG
2	C	15	ARG
2	C	139	ARG
2	C	234	GLN
2	C	241	LYS
2	C	251	LYS
1	D	15	ARG
1	D	99	ILE
1	D	129	TYR
1	D	140	SER
1	D	248	LYS

Sometimes sidechains can be flipped to improve hydrogen bonding and reduce clashes. All (4) such sidechains are listed below:

Mol	Chain	Res	Type
1	A	46	ASN

Continued on next page...

For Manuscript Review

Continued from previous page...

Mol	Chain	Res	Type
1	A	67	GLN
1	A	105	GLN
1	D	12	HIS

5.3.3 RNA (i)

There are no RNA molecules in this entry.

5.4 Non-standard residues in protein, DNA, RNA chains (i)

There are no non-standard protein/DNA/RNA residues in this entry.

5.5 Carbohydrates (i)

There are no oligosaccharides in this entry.

5.6 Ligand geometry (i)

6 ligands are modelled in this entry.

In the following table, the Counts columns list the number of bonds (or angles) for which Mogul statistics could be retrieved, the number of bonds (or angles) that are observed in the model and the number of bonds (or angles) that are defined in the Chemical Component Dictionary. The Link column lists molecule types, if any, to which the group is linked. The Z score for a bond length (or angle) is the number of standard deviations the observed value is removed from the expected value. A bond length (or angle) with $|Z| > 2$ is considered an outlier worth inspection. RMSZ is the root-mean-square of all Z scores of the bond lengths (or angles).

Mol	Type	Chain	Res	Link	Bond lengths			Bond angles		
					Counts	RMSZ	$\#\|Z\| > 2$	Counts	RMSZ	$\#\|Z\| > 2$
3	FAD	D	301	-	53,58,58	0.63	0	68,89,89	0.66	0
3	FAD	B	301	-	53,58,58	0.67	0	68,89,89	0.73	2 (2%)
3	FAD	C	301	-	53,58,58	0.64	0	68,89,89	0.88	4 (5%)
4	NAD	B	302	-	42,48,48	0.73	1 (2%)	50,73,73	0.86	3 (6%)
4	NAD	C	302	-	42,48,48	0.78	1 (2%)	50,73,73	0.85	2 (4%)
3	FAD	A	301	-	53,58,58	0.65	0	68,89,89	0.87	3 (4%)

In the following table, the Chirals column lists the number of chiral outliers, the number of chiral centers analysed, the number of these observed in the model and the number defined in the Chemical Component Dictionary. Similar counts are reported in the Torsion and Rings columns.

For Manuscript Review

'-' means no outliers of that kind were identified.

Mol	Type	Chain	Res	Link	Chirals	Torsions	Rings
3	FAD	D	301	-	-	1/30/50/50	0/6/6/6
3	FAD	B	301	-	-	1/30/50/50	0/6/6/6
3	FAD	C	301	-	-	1/30/50/50	0/6/6/6
4	NAD	B	302	-	-	8/26/62/62	0/5/5/5
4	NAD	C	302	-	-	9/26/62/62	0/5/5/5
3	FAD	A	301	-	-	2/30/50/50	0/6/6/6

All (2) bond length outliers are listed below:

Mol	Chain	Res	Type	Atoms	Z	Observed(Å)	Ideal(Å)
4	C	302	NAD	C2N-N1N	3.21	1.38	1.35
4	B	302	NAD	C2N-N1N	2.68	1.38	1.35

All (14) bond angle outliers are listed below:

Mol	Chain	Res	Type	Atoms	Z	Observed(^{o})	Ideal(^{o})
3	A	301	FAD	C5A-C6A-N6A	2.50	124.15	120.35
3	C	301	FAD	O2'-C2'-C1'	2.39	115.57	109.80
4	B	302	NAD	C5A-C6A-N6A	2.30	123.84	120.35
3	A	301	FAD	C5'-C4'-C3'	-2.27	107.82	112.20
4	C	302	NAD	C6N-N1N-C2N	-2.20	119.97	121.97
3	C	301	FAD	O4B-C1B-C2B	-2.20	103.71	106.93
3	B	301	FAD	C5A-C6A-N6A	2.19	123.68	120.35
3	C	301	FAD	O2P-P-O1P	2.18	123.03	112.24
4	C	302	NAD	C5A-C6A-N6A	2.16	123.64	120.35
3	C	301	FAD	C5A-C6A-N6A	2.16	123.64	120.35
3	B	301	FAD	O5'-C5'-C4'	2.14	115.08	109.36
4	B	302	NAD	O4D-C4D-C3D	-2.13	100.89	105.11
4	B	302	NAD	C6N-N1N-C2N	-2.12	120.04	121.97
3	A	301	FAD	C1B-N9A-C4A	-2.03	123.07	126.64

There are no chirality outliers.

All (22) torsion outliers are listed below:

Mol	Chain	Res	Type	Atoms
4	B	302	NAD	C5B-O5B-PA-O1A
4	B	302	NAD	C3D-C4D-C5D-O5D
4	C	302	NAD	C5B-O5B-PA-O1A
4	C	302	NAD	C5D-O5D-PN-O3
4	C	302	NAD	C2D-C1D-N1N-C2N

Continued on next page...

For Manuscript Review

Continued from previous page...

Mol	Chain	Res	Type	Atoms
4	C	302	NAD	C2D-C1D-N1N-C6N
4	B	302	NAD	O4B-C4B-C5B-O5B
4	B	302	NAD	O4D-C4D-C5D-O5D
4	C	302	NAD	O4B-C4B-C5B-O5B
4	C	302	NAD	C3B-C4B-C5B-O5B
4	B	302	NAD	C3B-C4B-C5B-O5B
4	B	302	NAD	C4D-C5D-O5D-PN
4	B	302	NAD	C5B-O5B-PA-O3
4	B	302	NAD	C5B-O5B-PA-O2A
4	C	302	NAD	C5D-O5D-PN-O2N
3	B	301	FAD	O4B-C4B-C5B-O5B
4	C	302	NAD	C4B-C5B-O5B-PA
3	A	301	FAD	P-O3P-PA-O1A
3	D	301	FAD	C4'-C5'-O5'-P
3	C	301	FAD	C4'-C5'-O5'-P
3	A	301	FAD	P-O3P-PA-O2A
4	C	302	NAD	O4D-C4D-C5D-O5D

There are no ring outliers.

5 monomers are involved in 16 short contacts:

Mol	Chain	Res	Type	Clashes	Symm-Clashes
3	D	301	FAD	2	0
3	B	301	FAD	3	0
3	C	301	FAD	1	0
4	B	302	NAD	7	0
4	C	302	NAD	3	0

The following is a two-dimensional graphical depiction of Mogul quality analysis of bond lengths, bond angles, torsion angles, and ring geometry for all instances of the Ligand of Interest. In addition, ligands with molecular weight > 250 and outliers as shown on the validation Tables will also be included. For torsion angles, if less then 5% of the Mogul distribution of torsion angles is within 10 degrees of the torsion angle in question, then that torsion angle is considered an outlier. Any bond that is central to one or more torsion angles identified as an outlier by Mogul will be highlighted in the graph. For rings, the root-mean-square deviation (RMSD) between the ring in question and similar rings identified by Mogul is calculated over all ring torsion angles. If the average RMSD is greater than 60 degrees and the minimal RMSD between the ring in question and any Mogul-identified rings is also greater than 60 degrees, then that ring is considered an outlier. The outliers are highlighted in purple. The color gray indicates Mogul did not find sufficient equivalents in the CSD to analyse the geometry.

For Manuscript Review

Ligand FAD D 301

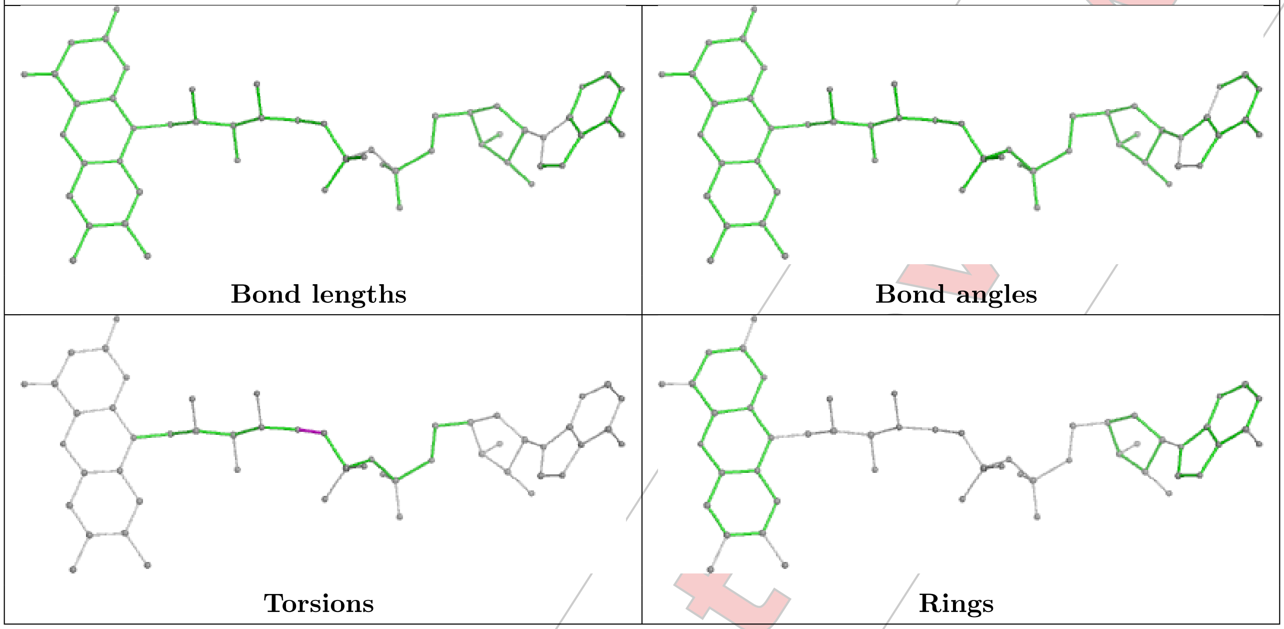

Ligand FAD B 301

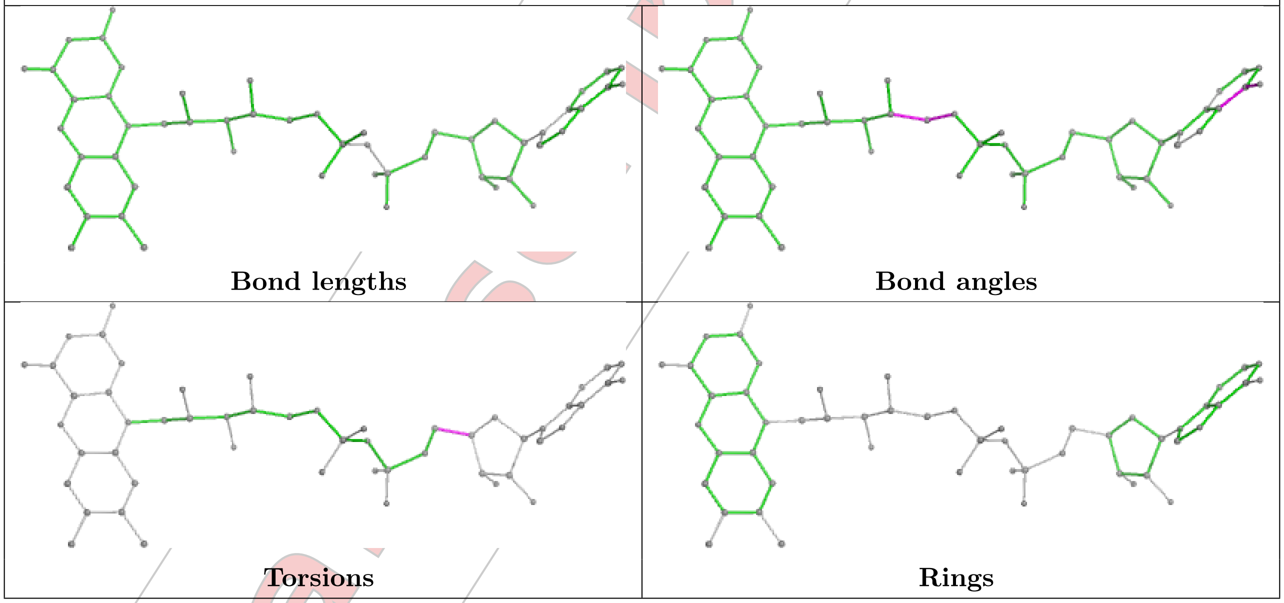

For Manuscript Review

Ligand FAD C 301

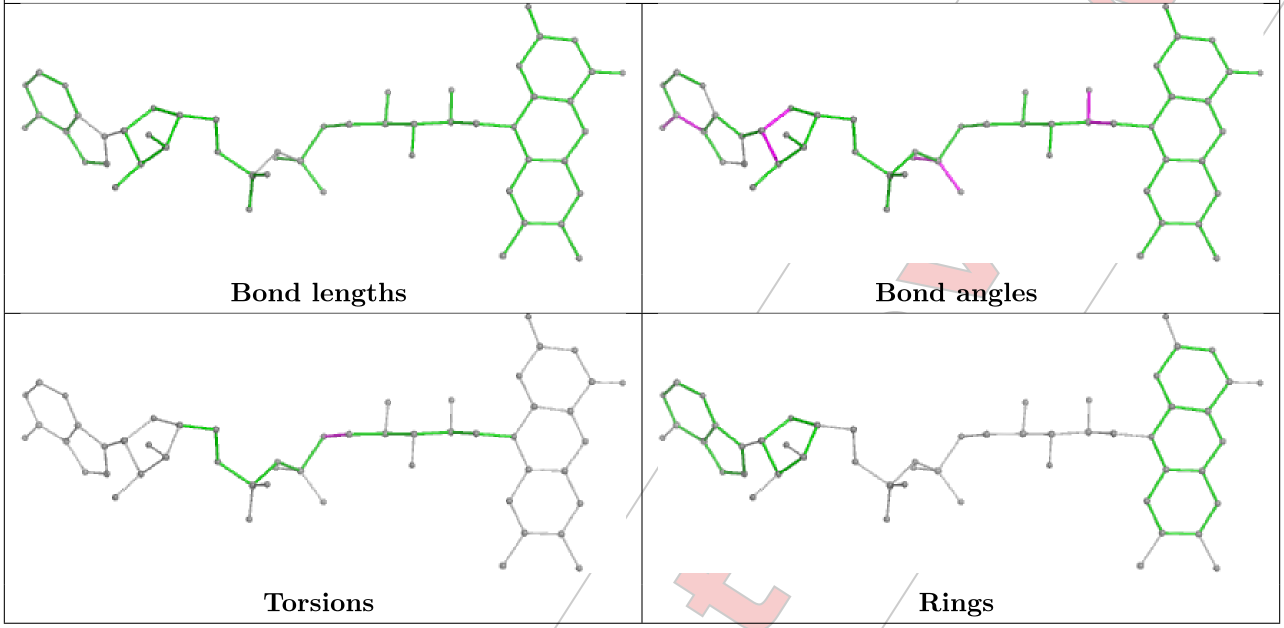

Ligand NAD B 302

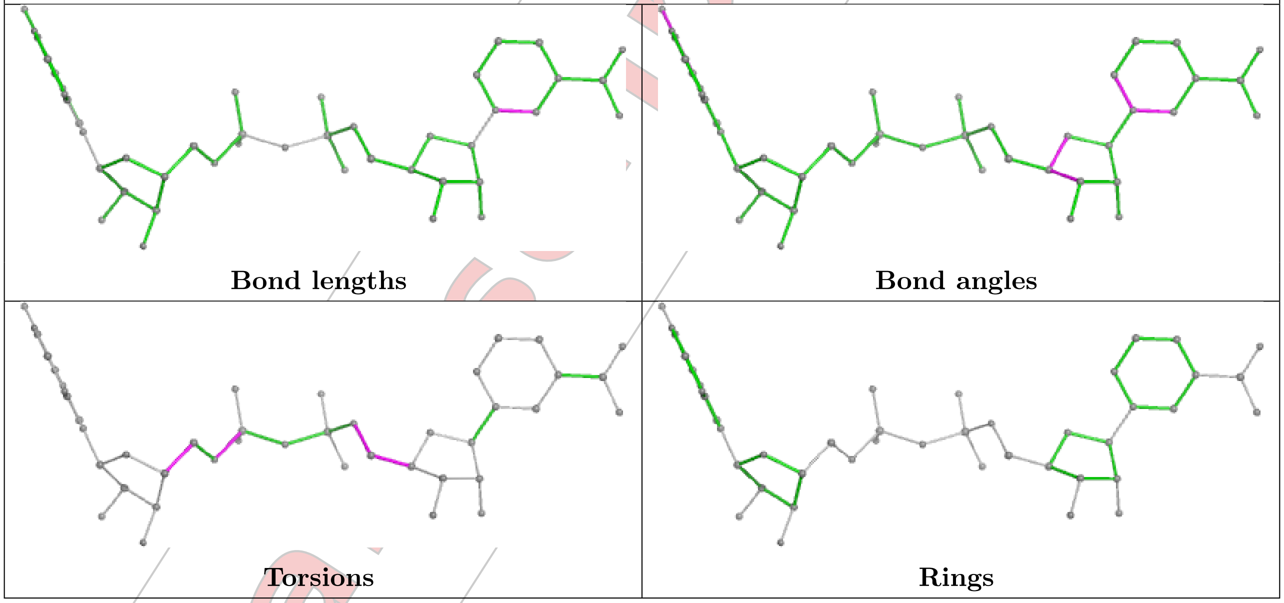

For Manuscript Review

Ligand NAD C 302

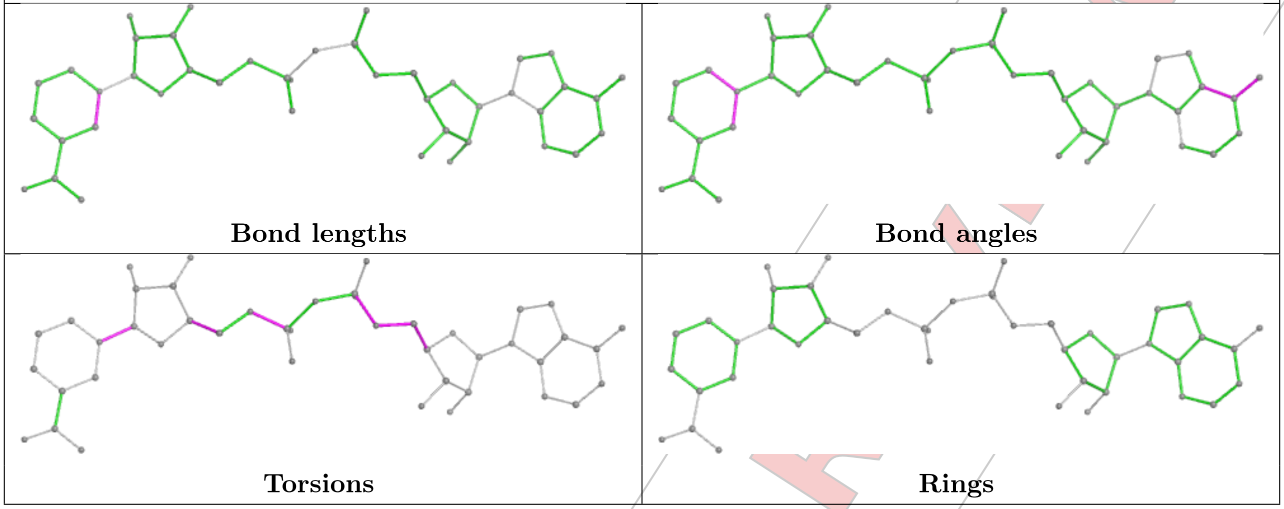

Ligand FAD A 301

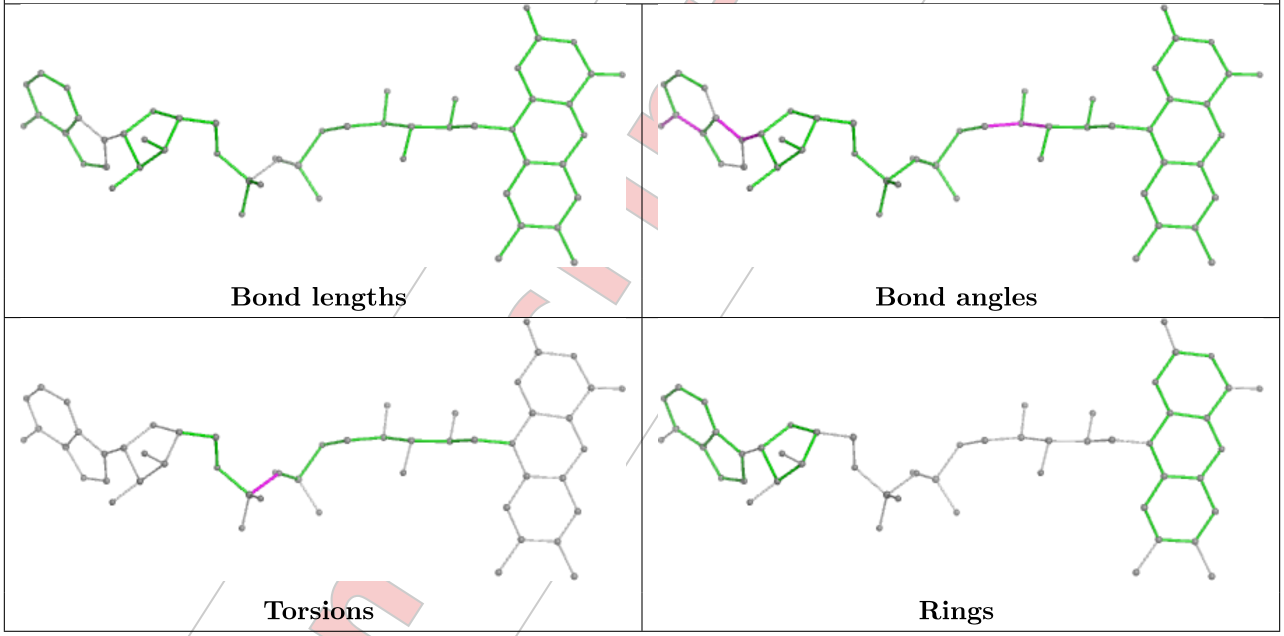

5.7 Other polymers (i)

There are no such residues in this entry.

5.8 Polymer linkage issues (i)

There are no chain breaks in this entry.

For Manuscript Review

6 Fit of model and data (i)

6.1 Protein, DNA and RNA chains (i)

In the following table, the column labelled '#RSRZ> 2' contains the number (and percentage) of RSRZ outliers, followed by percent RSRZ outliers for the chain as percentile scores relative to all X-ray entries and entries of similar resolution. The OWAB column contains the minimum, median, 95th percentile and maximum values of the occupancy-weighted average B-factor per residue. The column labelled 'Q< 0.9' lists the number of (and percentage) of residues with an average occupancy less than 0.9.

Mol	Chain	Analysed	<RSRZ>	#RSRZ>2			OWAB(Å^2)	Q<0.9
1	A	274/274 (100%)	-0.48	1 (0%)	89	86	25, 48, 96, 132	5 (1%)
1	B	271/274 (98%)	-0.48	0	100	100	26, 49, 86, 126	2 (0%)
1	D	273/274 (99%)	-0.34	1 (0%)	89	86	22, 56, 91, 129	2 (0%)
2	C	279/279 (100%)	-0.55	2 (0%)	84	81	25, 46, 88, 162	1 (0%)
All	All	1097/1101 (99%)	-0.46	4 (0%)	89	86	22, 50, 91, 162	10 (0%)

All (4) RSRZ outliers are listed below:

Mol	Chain	Res	Type	RSRZ
2	C	-4	ALA	2.3
1	A	2	VAL	2.1
1	D	2	VAL	2.1
2	C	-2	ALA	2.0

6.2 Non-standard residues in protein, DNA, RNA chains (i)

There are no non-standard protein/DNA/RNA residues in this entry.

6.3 Carbohydrates (i)

There are no monosaccharides in this entry.

6.4 Ligands (i)

In the following table, the Atoms column lists the number of modelled atoms in the group and the number defined in the chemical component dictionary. The B-factors column lists the minimum, median, 95th percentile and maximum values of B factors of atoms in the group. The column labelled 'Q< 0.9' lists the number of atoms with occupancy less than 0.9.

For Manuscript Review

Mol	Type	Chain	Res	Atoms	RSCC	RSR	B-factors($Å^2$)	Q<0.9
4	NAD	B	302	44/44	0.76	0.21	51,79,112,138	44
4	NAD	C	302	44/44	0.80	0.23	62,84,110,128	44
3	FAD	B	301	53/53	0.91	0.11	52,72,113,128	0
3	FAD	D	301	53/53	0.96	0.06	35,44,60,64	0
3	FAD	C	301	53/53	0.97	0.05	33,39,53,55	0
3	FAD	A	301	53/53	0.97	0.05	27,37,49,53	0

The following is a graphical depiction of the model fit to experimental electron density of all instances of the Ligand of Interest. In addition, ligands with molecular weight > 250 and outliers as shown on the geometry validation Tables will also be included. Each fit is shown from different orientation to approximate a three-dimensional view.

Electron density around NAD B 302:

2mF$_o$-DF$_c$ (at 0.7 rmsd) in gray
mF$_o$-DF$_c$ (at 3 rmsd) in purple (negative)
and green (positive)

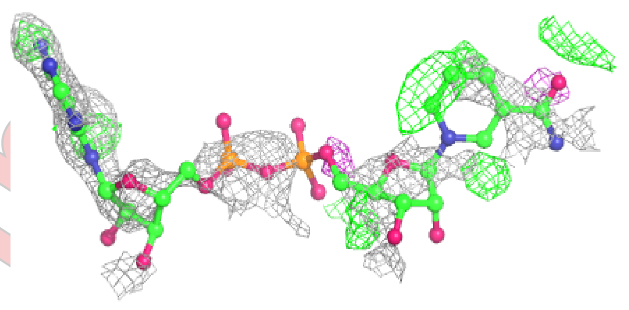

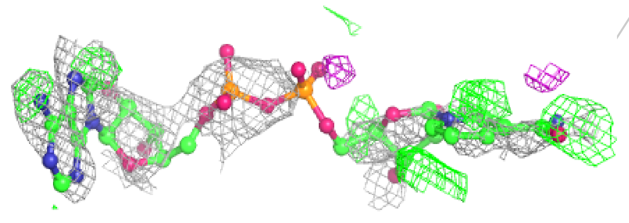

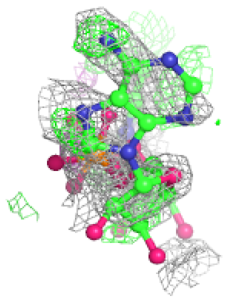

For Manuscript Review

Electron density around NAD C 302:

$2mF_o$-DF_c (at 0.7 rmsd) in gray
mF_o-DF_c (at 3 rmsd) in purple (negative)
and green (positive)

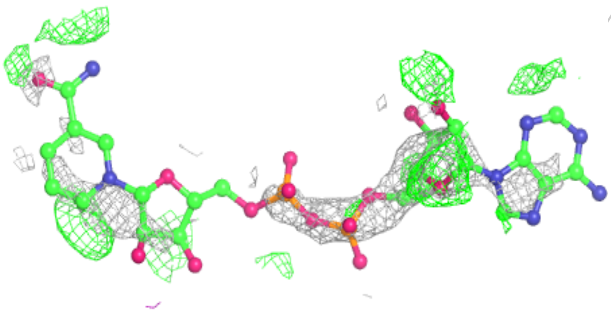

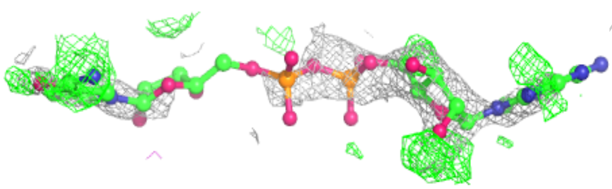

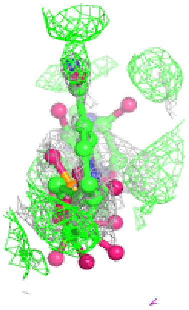

Electron density around FAD B 301:

$2mF_o$-DF_c (at 0.7 rmsd) in gray
mF_o-DF_c (at 3 rmsd) in purple (negative)
and green (positive)

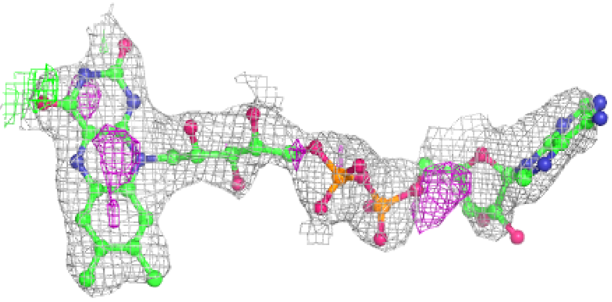

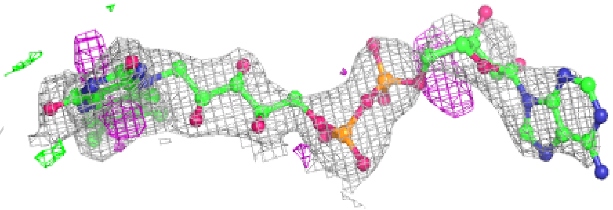

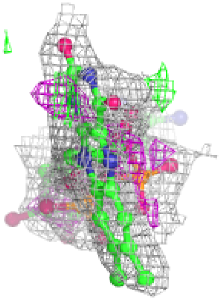

Electron density around FAD D 301:

$2mF_o$-DF_c (at 0.7 rmsd) in gray
mF_o-DF_c (at 3 rmsd) in purple (negative)
and green (positive)

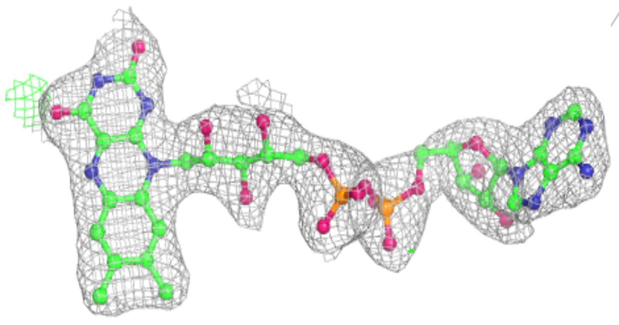

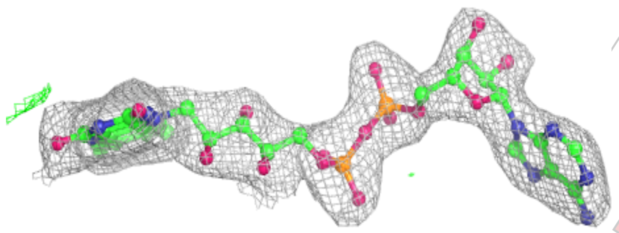

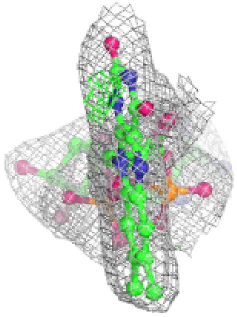

Electron density around FAD C 301:

$2mF_o$-DF_c (at 0.7 rmsd) in gray
mF_o-DF_c (at 3 rmsd) in purple (negative)
and green (positive)

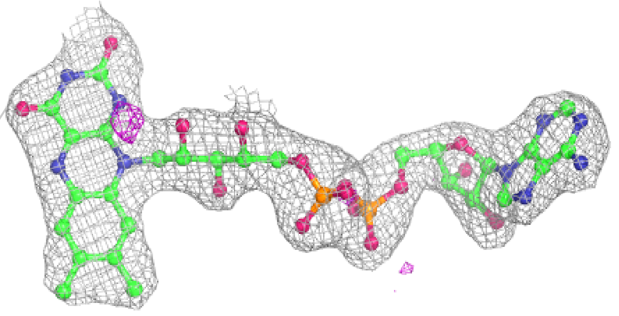

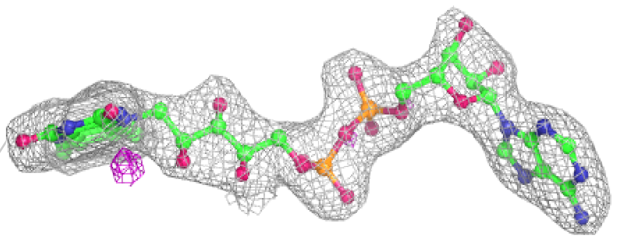

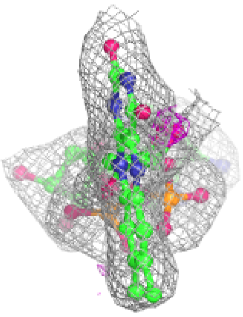

For Manuscript Review

Electron density around FAD A 301:

2mF$_o$-DF$_c$ (at 0.7 rmsd) in gray
mF$_o$-DF$_c$ (at 3 rmsd) in purple (negative)
and green (positive)

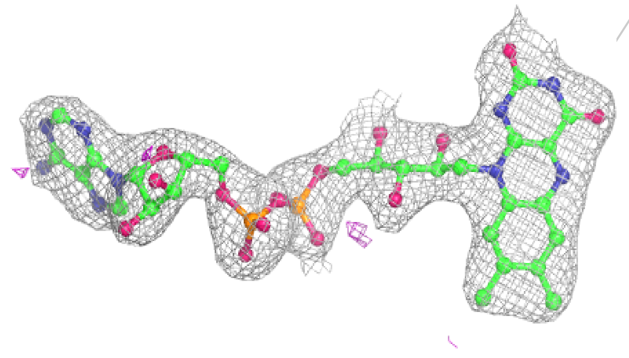

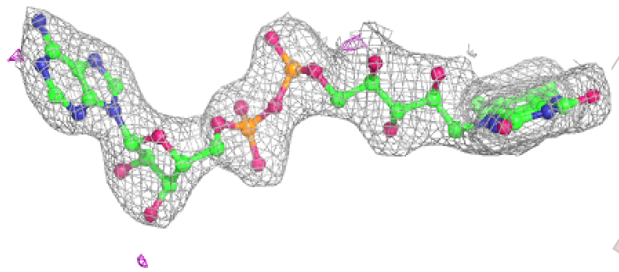

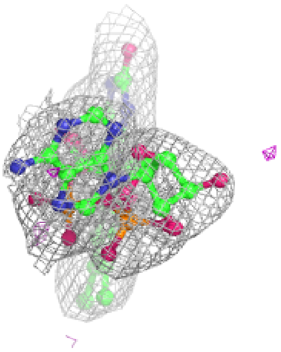

6.5 Other polymers (i)

There are no such residues in this entry.

For Manuscript Review
