# Supplementary material for: Minimized sample consumption for time-resolved serial crystallography applied to the redox cycle of human NQO1
Source: Commun Chem. 2026 Jan 29;9:107. doi: 10.1038/s42004-026-01908-9 (PMC12957379; doi:10.1038/s42004-026-01908-9)
Supplement: Supplementary file 9 — Reporting Summary [file 42004_2026_1908_MOESM9_ESM.pdf]

nature portfolio

Corresponding author(s): Alexandra Ros and Jose M. Martin Garcia

Last updated by author(s): 12/22/2025

Reporting Summary

Nature Portfolio wishes to improve the reproducibility of the work that we publish. This form provides structure for consistency and transparency in reporting. For further information on Nature Portfolio policies, see our Editorial Policies and the Editorial Policy Checklist.

Statistics

For all statistical analyses, confirm that the following items are present in the figure legend, table legend, main text, or Methods section.

n/a	Confirmed	
☒	☐	The exact sample size (*n*) for each experimental group/condition, given as a discrete number and unit of measurement
☒	☐	A statement on whether measurements were taken from distinct samples or whether the same sample was measured repeatedly
☒	☐	The statistical test(s) used AND whether they are one- or two-sided<br>*Only common tests should be described solely by name; describe more complex techniques in the Methods section.*
☒	☐	A description of all covariates tested
☒	☐	A description of any assumptions or corrections, such as tests of normality and adjustment for multiple comparisons
☒	☐	A full description of the statistical parameters including central tendency (e.g. means) or other basic estimates (e.g. regression coefficient) AND variation (e.g. standard deviation) or associated estimates of uncertainty (e.g. confidence intervals)
☒	☐	For null hypothesis testing, the test statistic (e.g. *F*, *t*, *r*) with confidence intervals, effect sizes, degrees of freedom and *P* value noted<br>*Give P values as exact values whenever suitable.*
☒	☐	For Bayesian analysis, information on the choice of priors and Markov chain Monte Carlo settings
☒	☐	For hierarchical and complex designs, identification of the appropriate level for tests and full reporting of outcomes
☒	☐	Estimates of effect sizes (e.g. Cohen's *d*, Pearson's *r*), indicating how they were calculated

Our web collection on statistics for biologists contains articles on many of the points above.

Software and code

Policy information about availability of computer code

Data collection	Room temperature data collection was performed using the AGIPD detector and with the EuXFEL developed code, employed at the SPB/SFX instrument. OnDA (Online Data Analysis) Monitor OM was used for live monitoring of the data collection process. Cheetah was used as hit-finder for the XFEL data. Both software are open source and available online.
Data analysis	Data processing was carried out with CrytFEL version 0.10.2, CCP4 version 8.0, COOT version 0.9.8.93. Numerical modeling with finite element software Comsol was used to estimate substrate / protein crystal mixing times. Fluorescence experimnts were conducted to monitor the mixing process.

For manuscripts utilizing custom algorithms or software that are central to the research but not yet described in published literature, software must be made available to editors and reviewers. We strongly encourage code deposition in a community repository (e.g. GitHub). See the Nature Portfolio guidelines for submitting code & software for further information.

Data

Policy information about availability of data

All manuscripts must include a data availability statement. This statement should provide the following information, where applicable:

- Accession codes, unique identifiers, or web links for publicly available datasets
- A description of any restrictions on data availability
- For clinical datasets or third party data, please ensure that the statement adheres to our policy

All data are included in the paper or supplementary information will be available from the corresponding author upon request. The structure factors and the refined coordinates of the NQO1 structures in its free form and mixed with NADH have been deposited in the PDB under the accession codes 9EZQ, 9EZS, 9EZR, and 9EZT.

Research involving human participants, their data, or biological material

Policy information about studies with human participants or human data. See also policy information about sex, gender (identity/presentation), and sexual orientation and race, ethnicity and racism.

Reporting on sex and gender	n/a
Reporting on race, ethnicity, or other socially relevant groupings	n/a
Population characteristics	n/a
Recruitment	n/a
Ethics oversight	n/a

Note that full information on the approval of the study protocol must also be provided in the manuscript.

Field-specific reporting

Please select the one below that is the best fit for your research. If you are not sure, read the appropriate sections before making your selection.

☒ Life sciences ☐ Behavioural & social sciences ☐ Ecological, evolutionary & environmental sciences

For a reference copy of the document with all sections, see nature.com/documents/nr-reporting-summary-flat.pdf

Life sciences study design

All studies must disclose on these points even when the disclosure is negative.

Sample size	Sample size in our manuscript relates to the consumption of NQO1 crystals, which was described in detail in the manuscript. We have only studied one protein, NQO1 in this study. Regarding data, sample size is not precalculated in experiment collected in serial mode. Instead, for each data set, data is collected unil the signal is noticeable stronger than the noise, so that the sample size varies for each data set. All data statistics and the quality of the data are reported accordingly in the manuscript.
Data exclusions	Due to the nature of serial crystallography, a large amount of images are recorded per experiment. The images without Bragg reflections do not contribute to crystallographic analysis and thus excluded from data analysis. Also, there has been one more time point recorded during our beam time, that we chose not to include since the data set was not complete.
Replication	The protein crystallography experiment as presented in this manuscript, could not be repeated due to the lack of beam time at the EuXFEL. However, we have repeteadly obtained the NQO1 structure from an XFEL beam time, including one at the Linac Coherent Light Source at the SLAC Linear Accelerator Laboratory, published in D. Doppler, et al. Lab on a Chip (2023), 23, 3016-3022, DOI: 10.1039/D3LC00176H.
Randomization	The orientation of the microcrystals in serial crystallogrpahy is random by nature. Each diffraction pattern is collected from randomly oriented crystals that are injected in serial mode. Hundreds of thousands of diffraction patterns are collected for each data set.
Blinding	Binding is not applicable in this experiment as the conidtions of data collection depend on the pre-determined time points. Each time point is a separate data set.

Reporting for specific materials, systems and methods

We require information from authors about some types of materials, experimental systems and methods used in many studies. Here, indicate whether each material, system or method listed is relevant to your study. If you are not sure if a list item applies to your research, read the appropriate section before selecting a response.

Materials & experimental systems

n/a	Involved in the study
☒	☐ Antibodies
☒	☐ Eukaryotic cell lines
☒	☐ Palaeontology and archaeology
☒	☐ Animals and other organisms
☒	☐ Clinical data
☒	☐ Dual use research of concern
☒	☐ Plants

Methods

n/a	Involved in the study
☒	☐ ChIP-seq
☒	☐ Flow cytometry
☒	☐ MRI-based neuroimaging

Plants

Seed stocks	n/a
Novel plant genotypes	n/a
Authentication	n/a
